# Supplementary material for: Photochemical conversion of CO to C1 and C2 products mediated by porphyrin rhodium(II) metallo-radical complexes
Source: Nat Commun. 2024 Sep 4;15:7724. doi: 10.1038/s41467-024-50253-9 (PMC11374781; doi:10.1038/s41467-024-50253-9)
Supplement: Supplementary file 1 — Supplementary Information [file 41467_2024_50253_MOESM1_ESM.pdf]

Supplementary information for

Photochemical conversion of CO to C1 and C2 products mediated by porphyrin rhodium(II) metallo-radical complexes

Hongsen Li[1†], Boao Han[1†], Rongyi Wang[1], Wentao Li[2], Wentao Zhang[2], Xuefeng Fu[2], Huayi Fang[3], Fuqiu Ma[4], Zikuan Wang[5*], Jiajing Zhang[1*]

[1]School of Pharmacy, Binzhou Medical University, Yantai 264003, China.
[2]Beijing National Laboratory for Molecular Sciences, State Key Lab of Rare Earth Materials Chemistry and Applications, College of Chemistry and Molecular Engineering, Peking University, Beijing 100871, China.
[3]School of Materials Science and Engineering, Tianjin Key Lab for Rare Earth Materials and Applications, Nankai University, Tianjin 300350, China.
[4]Yantai Research Institute of Harbin Engineering University, Yantai 264000, China.
[5]Max-Planck-Institut für Kohlenforschung, Kaiser-Wilhelm-Platz 1, Mülheim an der Ruhr 45470, Germany.
† H. Li and B. Han contributed equally to this work.
*Corresponding author. Email: (jiajing_z@bzmc.edu.cn (J.Z.); zwang@kofo.mpg.de (Z.W.))

Table of Content

Supplementary Methods......2

General considerations......2

Synthesis and characterization of (TPP)Rh$CH_2OSiR_1R_2R_3$......4

Release products and characterization of $XCH_2OSiR^1R^2R^3$(X = TEMPO, H, Br, D)......27

Synthesis and characterization of (TPP)Rh$COCH_2OSiR^1R^2R^3$......39

Product release and characterization of $XCOCH_2OSiR^1R^2R^3$ (X = ^{n}PrNH, TEMPO, Br)61

Supplementary Discussion......67

Supplementary References......74

Supplementary Methods

General considerations

C_6D_6, CD_3OD, CH_3OD, D_2O and $CDCl_3$ were purchased from Cambridge Isotope Laboratory Inc.; $[Rh(CO)_2Cl]_2$ from Strem Chemical Inc.; and all other chemicals were purchased from Sigma-Aldrich, Alfa Aesar, Tokyo Chemical Industry (TCI) or J&K Scientific Ltd. and used as received. ^1H NMR spectra were recorded on a Bruker AVII-400 spectrometer at room temperature. The chemical shifts were referenced to solvent residual signals. GC-MS results were obtained by the Agilent 7980A/5975C GC/MSD system. ESI-MS results were obtained by a Bruker Apex IV FTMS. The FTIR microspectroscopy of complexes were recorded on a NICOLET iN10 MX spectrometer in the range of 4000-600 cm^{-1}. The irradiation experiments were conducted using a 500 W high-pressure mercury lamp (CHF-XM 35-500W, Beijing Trusttech Co., Ltd.,) equipped with a 420−780 nm filter, and the glass vessel was positioned about 10.0 cm away from the light source.

All calculations were conducted with a development version of ORCA.[1] Geometry optimizations and single point energy calculations, including excited state calculations, were performed using the PBE0[2] functional, with the spin free X2C relativistic Hamiltonian[3] and the x2c-TZVPall[4] basis set. The RIJCOSX approximation[5] was used together with the x2c/J[4] Coulomb fitting set. Dispersion effects were account for by means of Grimme's DFT-D3 correction with Becke-Johnson (BJ) damping.[6] Harmonic frequencies were calculated for converged structures to verify the nature of the stationary points. Gibbs free energies based on the standard state of ideal gas (298 K, 1 atm) were corrected to the standard state of solution (298 K, 1 mol/L) by adding 1.89 kcal/mol,[7] except for CO where the gas-phase Gibbs free energies were used. The CPCM solvation model[8] was employed in all calculations, with benzene as solvent. Similar levels of theory have been successfully applied by one of us (Z.W.) in the study of the photophysics of porphyrin copper(II) complexes, giving excitation energies, quantum yields and rate constants that agreed quantitatively with experiment and high-level computational results.[9]

All excited state energy, geometry optimization and frequency calculations (except the T_1 state) were conducted with the Tamm-Dancoff approximation (TDA), under the aforementioned level of theory. For the T_1 state, all calculations except potential energy curves were done at the unrestricted Kohn-Sham (UKS) level; the treatment of triplet potential energy curves will be detailed later. Absorption spectra were computed at the full TDDFT level instead of the TDA level, to exploit the better transition dipole moments at the full TDDFT level compared to the TDA level.

In the dissociation regions of the Rh-C(alkyl) bonds, the closed-shell singlet solution of the ground state becomes unstable, in which case TDA calculations were performed upon broken symmetry singlet ground state wavefunctions. This may lead to some quantitative errors in the excited states in the dissociation region, particularly the lowest triplet ^{3}(σ(Rh-C(alkyl)) → σ*(Rh-C(alkyl))) state; in particular, the TDA energy of the ^{3}(σ(Rh-C(alkyl))→σ*(Rh-C(alkyl))) state does not reproduce the correct dissociation limit. Therefore, beyond the Coulson-Fischer point (i.e. the Rh-C bond length where the ground state becomes a broken-shell singlet; shown as vertical grey dashed lines in **Supplementary Figures 94-96**), we used UKS instead of TDA for the ^{3}(σ(Rh-C(alkyl))→σ*(Rh-C(alkyl))) state. Close to the ground state equilibrium structure, however, the ^{3}(σ(Rh-C(alkyl))→ σ*(Rh-C(alkyl))) excitation mixes extensively with other states, and it proved difficult to converge to the ^{3}(σ(Rh-C(alkyl))→σ*(Rh-C(alkyl))) state robustly using the UKS approach; thus, for Rh-C bond lengths smaller than the Coulson-Fischer point, we used TDA for all excited state potential energy curves. Whenever possible, excited states at different Rh-C bond lengths are connected based on state compositions instead of energy rankings. Due to extensive excited state mixings, in some cases the correspondence of excited states at neighboring geometries may be ambiguous, and a more or less arbitrary choice has to be made.

Orbital diagrams were visualized with the in-house MATLAB code “BMV”, available for free upon request.

Synthesis and characterization of (TPP)RhCH$_2$OSiR$_1$R$_2$R$_3$

36 mg of (TPP)RhI was dissolved in a 25 mL Schlenk flask with 2.0 mL of toluene, 20 µL of silane and 3 mg of B(C$_6$F$_5$)$_3$. After three freeze-pump-thaw cycles, 3 atm of CO was added. The solution was stirred at room temperature for 12 h, and then evaporated to dryness under reduced pressure, followed by column chromatography on silica gel with PE:Et$_2$O = 10:1 as eluent. An orange fraction was collected, then purified again by column chromatography on silica gel with PE:DCM = 3:1. The second red-orange component was collected and evaporated to dryness and identified as (TPP)RhCH$_2$OSiR^{1}R^{2}R^3 (**6**). When using HSiEt$_3$ instead of DSiEt$_3$ (99 % D), we obtained (TPP)RhCD$_2$OSiEt$_3$ (99 % D). When using ^{13}CO (> 99.9%) instead CO, we obtained (TPP)Rh^{13}CH$_2$OSiEt$_3$ (> 99% ^{13}C).

Supplementary Table 1 Substrate scope of silanes and product release of (TPP)RhCH$_2$OSiR^{1}R^{2}R^3.

Entry	Substrate	Product	Yield	Released Product	Yield
1	HSiEt$_3$ **5a**	(TPP)RhCH$_2$OSiEt$_3$ **6a**	> 99%	CH$_3$OSiEt$_3$ **7a**	99%^{i}, 99%^{ii}
2	HSiMe$_2$Et **5b**	(TPP)RhCH$_2$OSiMe$_2$Et **6b**	> 99%	CH$_3$OSiMe$_2$Et **7b**	99%^{i}, 99%^{ii}
3	HSi^{n}Pr$_3$ **5c**	(TPP)RhCH$_2$OSi^{n}Pr$_3$ **6c**	> 99%	CH$_3$OSi^{n}Pr$_3$ **7c**	95%^{i}, 99%^{ii}
4	HSiMe$_2$Ph **5d**	(TPP)RhCH$_2$OSiMe$_2$Ph **6d**	> 99%	CH$_3$OSiMe$_2$Ph **7d**	99%^{i}, 99%^{ii}
5	HSiMePh$_2$ **5e**	(TPP)RhCH$_2$OSiMePh$_2$ **6e**	> 99%	CH$_3$OSiMePh$_2$ **7e**	98%^{i}, 99%^{ii}

^{i}NMR yield with silane as hydrogen atom donor. ^{ii}NMR yield with (TPP)RhH as hydrogen atom donor.

(TPP)RhCH$_2$OSiEt$_3$ (6a): ^{1}H NMR (400 MHz, C$_6$D$_6$) δ (ppm): 8.90 (s, 8 H, pyrrole), 8.30 (m, 4 H, *o*-phenyl), 8.23 (m, 4 H, *o'*-phenyl), 7.50 (m, 12 H, *m*-phenyl, *p*-phenyl), -0.21 (t, 9 H, $^3J_{H\text{-}H}$= 8.0 Hz, Si-CH$_2$C$\underline{H}_3$), -1.19 (q, 6 H, $^3J_{H\text{-}H}$ = 8.0 Hz, Si-C$\underline{H}_2$CH$_3$), -1.72 (d, 2 H, $^2J_{Rh\text{-}H}$ = 3.4 Hz, Si-O-C$\underline{H}_2$-Rh); ^{13}C NMR (APT, 101 MHz, C$_6$D$_6$) δ (ppm): 143.54 (s, porphyrin carbon), 143.18 (s, porphyrin carbon), 134.64 (s, porphyrin carbon), 134.30 (s, porphyrin carbon), 131.86 (s, porphyrin carbon), 127.00 (s, porphyrin carbon), 122.68 (s, porphyrin carbon), 52.79 (d, $^1J_{Rh\text{-}C}$ =

28.2 Hz, Si-O-$\underline{C}H_2$-Rh), 6.05 (s, Si-$CH_2\underline{C}H_3$), 2.85 (s, Si-$\underline{C}H_2CH_3$). UV-Vis: λ_{abs} (nm) (toluene) 422, 533, 570. HR-ESI-MS m/z calcd for $C_{51}H_{45}N_4ORhSi$, $[M]^+$ 860.24121; found 860.24087. Single crystals suitable for XRD studies were obtained by slow diffusion from a concentrated THF solution to deionized water.

(TPP)RhCH$_2$OSiMe$_2$Et (6b): ^{1}H NMR (400 MHz, C_6D_6) δ (ppm): 8.89 (s, 8 H, pyrrole), 8.25 (m, 8 H, *o*-phenyl), 7.50 (m, 12 H, *m*-phenyl, *p*-phenyl), -0.20 (t, 3 H, $^3J_{H\text{-}H}$ = 8.0 Hz, Si-$CH_2C\underline{H_3}$), -1.20 (q, 2 H, $^3J_{H\text{-}H}$ = 8.0 Hz, Si-$C\underline{H_2}CH_3$), -1.69 (s, 6 H, Si-$C\underline{H_3}$), -1.74 (d, 2 H, $^2J_{Rh\text{-}H}$ = 3.5 Hz, Si-O-$C\underline{H_2}$-Rh); ^{13}C NMR (APT, 101 MHz, C_6D_6) δ (ppm): 143.16 (s, porphyrin carbon), 142.77 (s, porphyrin carbon), 134.21 (s, porphyrin carbon), 134.00 (s, porphyrin carbon), 131.50 (s, porphyrin carbon), 126.61 (s, porphyrin carbon), 122.36 (s, porphyrin carbon), 51.76 (d, $^1J_{Rh\text{-}C}$ = 28.0 Hz, Si-O-$\underline{C}H_2$-Rh), 6.12 (s, Si-$\underline{C}H_2CH_3$), 5.58 (s, Si-$CH_2\underline{C}H_3$), -5.30 (s, Si-$C\underline{H_3}$). UV-Vis: λ_{abs} (nm) (toluene) 414, 521, 571. HR-ESI-MS m/z calcd for $C_{49}H_{41}N_4ORhSi$, $[M]^+$ 832.21047; found 832.21051. Single crystals suitable for XRD studies were obtained by slow diffusion from a concentrated THF solution to deionized water.

(TPP)RhCH$_2$OSi^{n}Pr$_3$ (6c): ^{1}H NMR (400 MHz, C_6D_6) δ (ppm): 8.89 (s, 8 H, pyrrole), 8.32 (m, 4 H, *o*-phenyl), 8.23 (m, 4 H, *o'*-phenyl), 7.51 (m, 12 H, *m*-phenyl, *p*-phenyl), 0.42 (t, 9 H, $^3J_{H\text{-}H}$ = 8.0 Hz, Si-$CH_2CH_2C\underline{H_3}$), 0.03 (m, 6 H, Si-$CH_2C\underline{H_2}CH_3$), -1.18 (m, 6 H, Si-$C\underline{H_2}CH_2CH_3$) , -1.76(d, 2 H, $^2J_{H\text{-}H}$ = 3.5 Hz, Si-O-$C\underline{H_2}$-Rh); ^{13}C NMR (APT, 101 MHz, C_6D_6) δ (ppm): 143.52 (s, porphyrin carbon), 143.17 (s, porphyrin carbon), 134.58 (s, porphyrin carbon), 134.35 (s, porphyrin carbon), 131.85 (s, porphyrin carbon), 126.97 (d, porphyrin carbon, $^2J_{Rh\text{-}H}$ = 7.7 Hz), 122.65 (s, porphyrin carbon), 52.84 (d, $^1J_{Rh\text{-}C}$ = 28.2 Hz, Si-O-$\underline{C}H_2$-Rh), 18.10 (s, Si-$CH_2CH_2\underline{C}H_3$), 16.04 (s, Si-$CH_2\underline{C}H_2CH_3$), 14.73 (s, Si-$\underline{C}H_2CH_2CH_3$); HR-ESI-MS m/z calcd for $C_{54}H_{51}N_4ORhSi$, $[M]^+$ 902.28872; found 902.28673.

(TPP)RhCH$_2$OSiMe$_2$Ph (6d): ^{1}H NMR (400 MHz, C_6D_6) δ (ppm): 8.88 (s, 8 H, pyrrole), 8.23 (m, 4 H, *o*-phenyl), 8.10 (m, 4 H, *o'*-phenyl), 7.50 (m, 12 H, *m*-phenyl, *p*-phenyl), 6.85 (m, 1 H, Si-phenyl), 6.72 (m, 2 H, Si-phenyl), 5.97 (m, 2 H, Si-phenyl), -1.50 (s, 6 H, Si-$C\underline{H_3}$), -1.65 (d, 2 H, ^2JRh-H = 3.5 Hz, Si-O-$C\underline{H_2}$-Rh); ^{13}C NMR (APT, 101 MHz, C_6D_6) δ (ppm): 143.52 (s, porphyrin carbon), 143.06 (s, porphyrin carbon), 134.46 (s, porphyrin carbon), 132.71 (s,

porphyrin carbon), 131.91 (s, porphyrin carbon),126.96 (d, porphyrin carbon, $^{2}J_{C\text{-}C}$ = 6.4 Hz), 122.79 (s, porphyrin carbon), 51.63 (d, $^{1}J_{Rh\text{-}C}$ = 28.6 Hz, Si-O-$\underline{C}H_2$-Rh), 3.98 (s, Si-$\underline{C}H_3$), other peaks are not obvious because of low concentration. UV-Vis: λ_{abs} (nm) (toluene) 414, 521, 549. HR-ESI-MS m/z calcd for $C_{53}H_{41}N_4ORhSi$, $[M]^+$ 880.21047; found 880.21042. Single crystals suitable for XRD studies were obtained by slow diffusion from a concentrated THF solution to deionized water.

(TPP)RhCH$_2$OSiMePh$_2$ (6e): ^{1}H NMR (400 MHz, C_6D_6) δ (ppm): 8.88 (s, 8 H, pyrrole), 8.24 (m, 4 H, *o*-phenyl), 7.94 (m, 4 H, *o'*-phenyl), 7.50 (m, 8 H, *m*-phenyl), 7.42 (m, 4 H, *p*-phenyl), 6.85 (m, 2 H, Si-phenyl), 6.72 (m, 4 H, Si-phenyl), 5.98 (m, 4 H, Si-phenyl), -1.36 (s, 3 H, Si-C$\underline{H}_3$), -1.56 (d, 2 H, $^{2}J_{Rh\text{-}H}$ = 3.5 Hz, Si-O-C$\underline{H}_2$-Rh); ^{13}C NMR (APT, 101 MHz, C_6D_6) δ (ppm): 143.50 (s, porphyrin carbon), 143.03 (s, porphyrin carbon), 134.52 (s, porphyrin carbon), 134.33 (s, porphyrin carbon), 133.52 (s, Si-phenyl), 131.92 (s, porphyrin carbon), 129.25 (s, Si-phenyl), 126.96 (s, Si-phenyl), 126.87 (s, Si-phenyl),126.91 (d, porphyrin carbon, $^{2}J_{C\text{-}C}$ = 8.8 Hz), 122.83 (s, porphyrin carbon), 51.48 (d, $^{1}J_{Rh\text{-}H}$ = 29.2 Hz, Si-O-$\underline{C}H_2$-Rh), 6.29 (s, Si-$\underline{C}H_3$); HR-ESI-MS m/z calcd for $C_{58}H_{43}N_4ORhSi$, $[M]^+$ 942.22612; found 942.22706.

(TPP)RhCD$_2$OSiEt$_3$ (6f): ^{1}H NMR (400 MHz, C_6D_6) δ (ppm): 8.90 (s, 8 H, pyrrole), 8.30 (m, 4 H, *o*-phenyl), 8.23 (m, 4 H, *o'*-phenyl), 7.50 (m, 12 H, *m*-phenyl, *p*-phenyl), -0.21 (t, 9 H, $^{3}J_{H\text{-}H}$= 8.0 Hz, Si-CH$_2$C$\underline{H}_3$), -1.19 (q, 6 H, $^{3}J_{H\text{-}H}$ = 8.0 Hz, Si-C$\underline{H}_2$CH$_3$).

(TPP)Rh^{13}CH$_2$OSiEt$_3$ (6g): ^{1}H NMR (400 MHz, C_6D_6) δ (ppm): 8.90 (s, 8 H, pyrrole), 8.30 (m, 4 H, *o*-phenyl), 8.23 (m, 4 H, *o'*-phenyl), 7.50 (m, 12 H, *m*-phenyl, *p*-phenyl), -0.21 (t, 9 H, $^{3}J_{H\text{-}H}$= 8.0 Hz, Si-CH$_2$C$\underline{H}_3$), -1.19 (q, 6 H, $^{3}J_{H\text{-}H}$ = 8.0 Hz, Si-C$\underline{H}_2$CH$_3$), -1.73 (dd, 2 H, $^{1}J_{13C\text{-}H}$ = 160 Hz, $^{2}J_{Rh\text{-}H}$ = 3.4 Hz, Si-O-^{13}C$\underline{H}_2$-Rh); ^{13}C NMR (APT, 101 MHz, C_6D_6) δ (ppm): 143.54 (s, porphyrin carbon), 143.18 (s, porphyrin carbon), 134.64 (s, porphyrin carbon), 134.30 (s, porphyrin carbon), 131.86 (s, porphyrin carbon), 127.00 (s, porphyrin carbon), 122.68 (s, porphyrin carbon), 52.41 (d, $^{1}J_{Rh\text{-}C}$ = 28.2 Hz, Si-O-$\underline{C}H_2$-Rh), 5.67 (s, Si-CH$_2\underline{C}H_3$), 2.47 (s, Si-$\underline{C}H_2$CH$_3$); HR-ESI-MS m/z calcd for $C_{50}H_{45}N_4O^{13}CRhSi$, $[M]^+$ 861.24457; found 861.24476.

Summary for spectroscopy

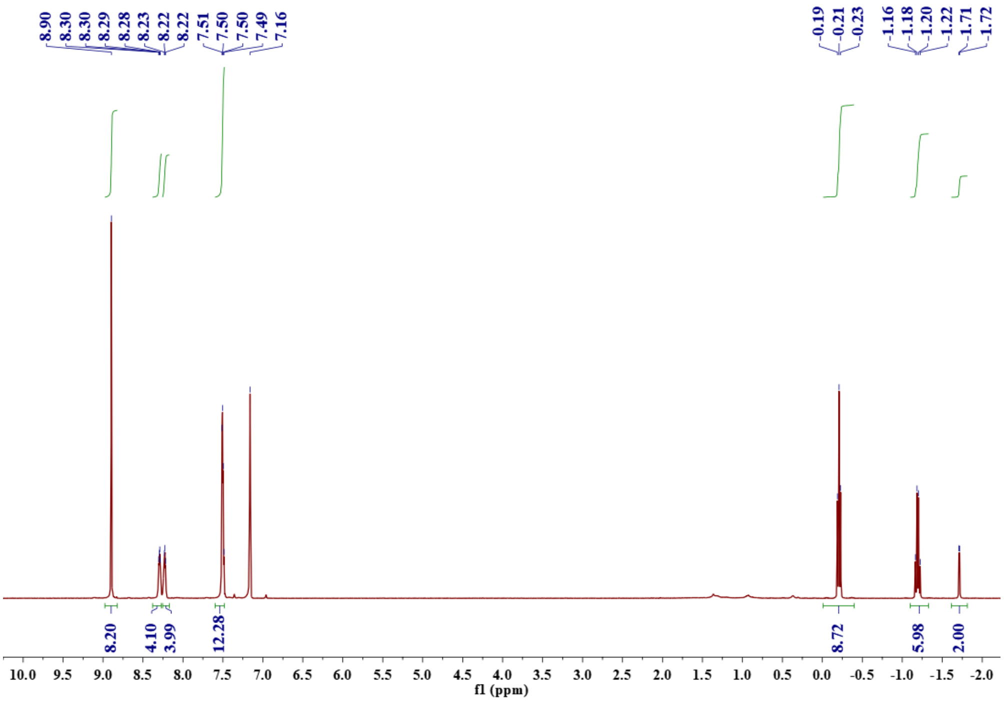

Supplementary Figure 1 ^{1}H NMR (400 MHz, C_6D_6, 25 °C) spectrum of **6a.**

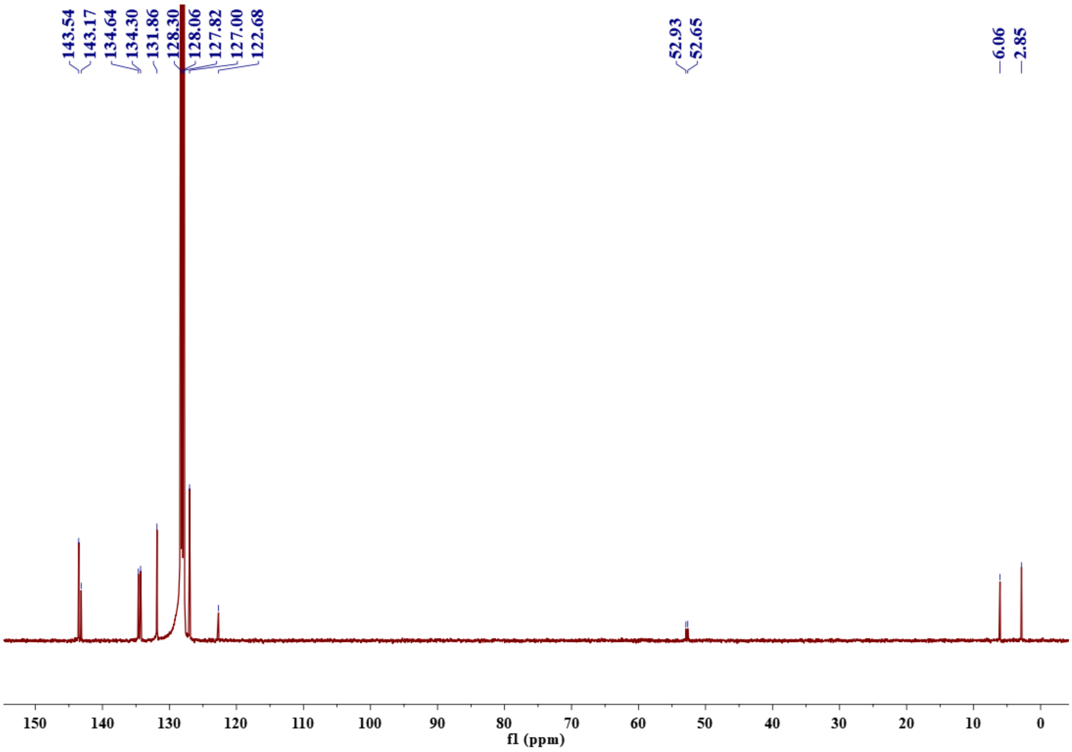

Supplementary Figure 2 ^{13}C NMR (101 MHz, C_6D_6, 25 °C) spectrum of **6a.**

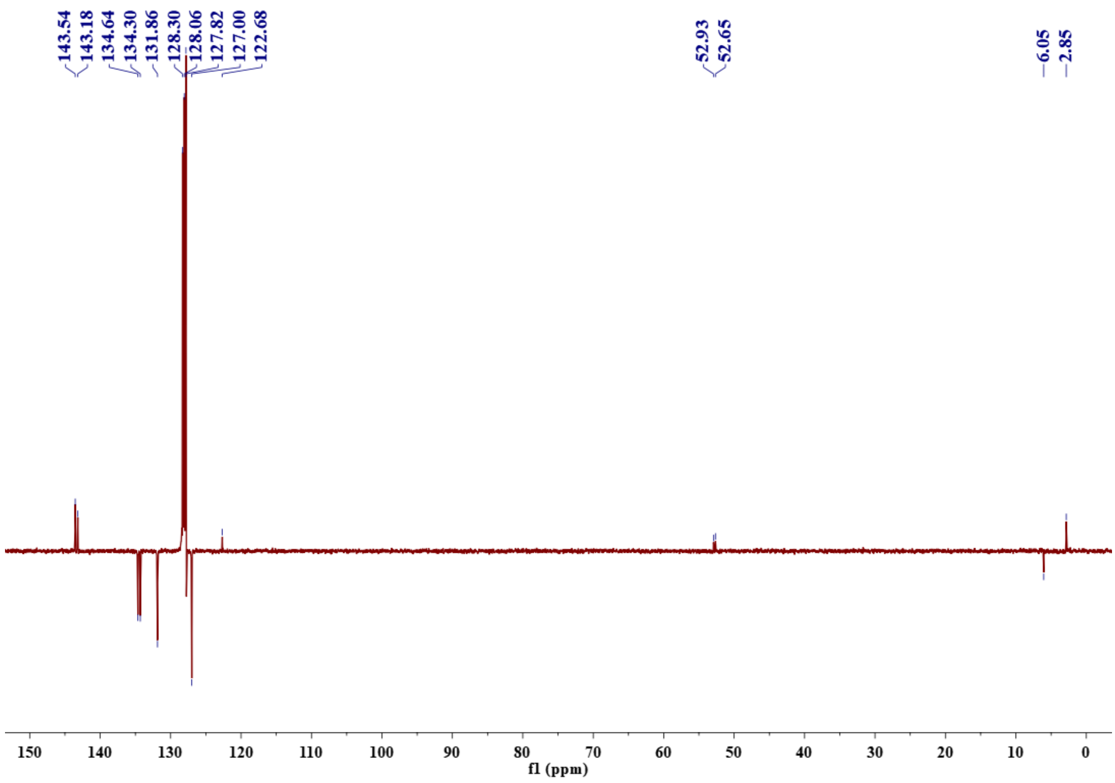

Supplementary Figure 3 ^{13}C APT NMR (101 MHz, C_6D_6, 25 °C) spectrum of **6a.**

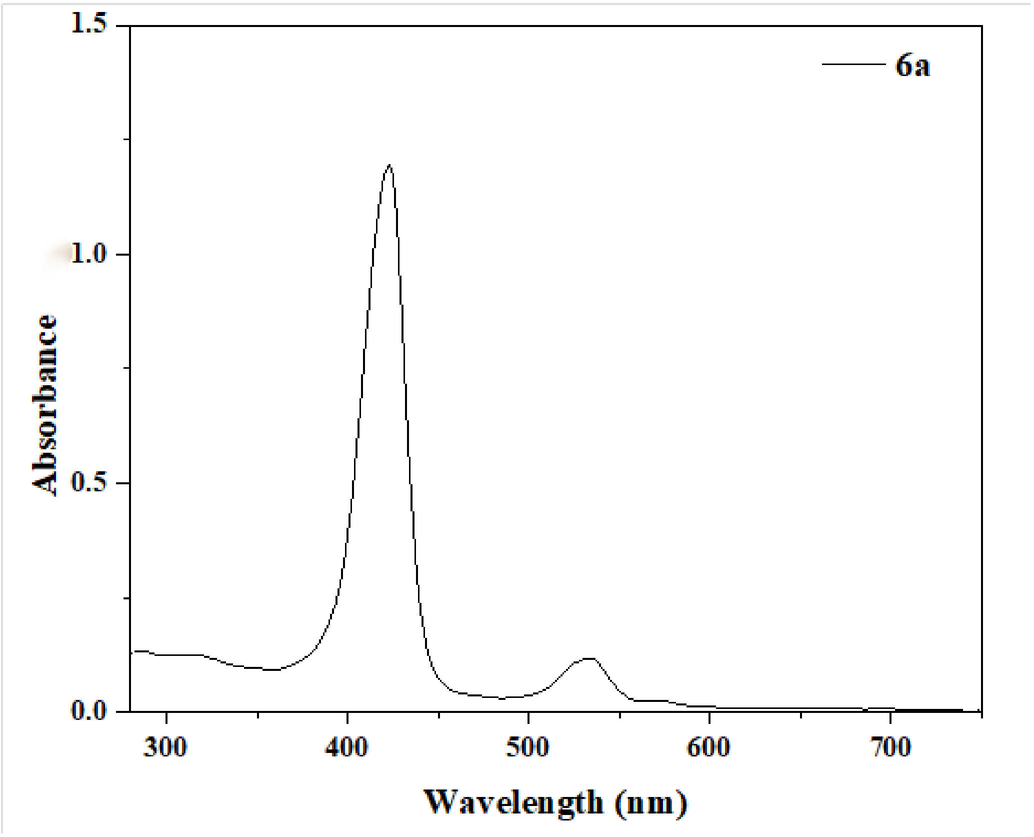

Supplementary Figure 4 UV-Vis spectrum of **6a** in toluene.

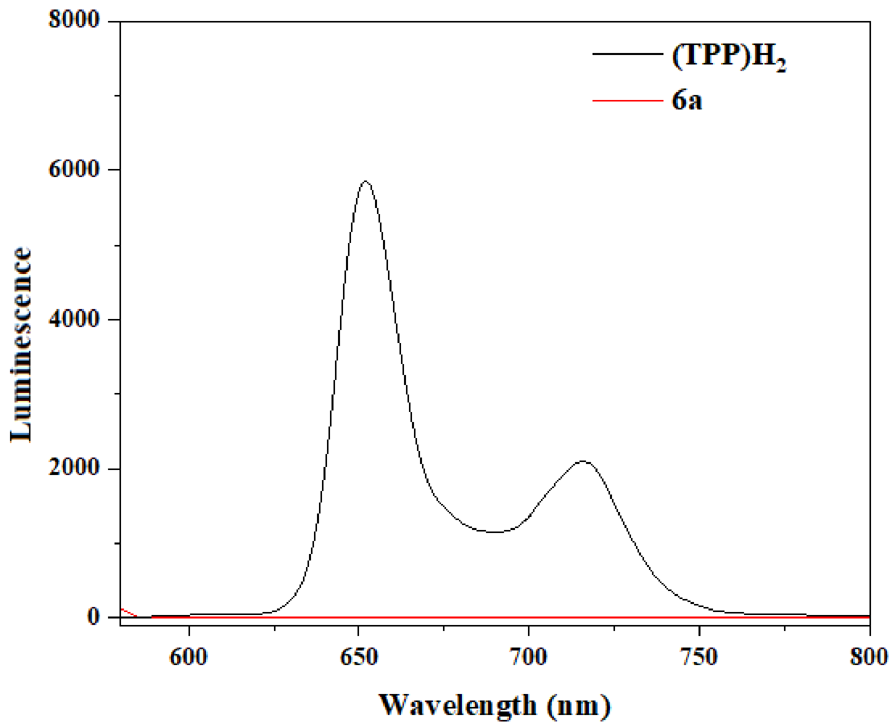

Supplementary Figure 5 Luminescence spectrum of **6a** in toluene, $(TPP)H_2$ for reference. No appreciable luminescence of **6a** was found.

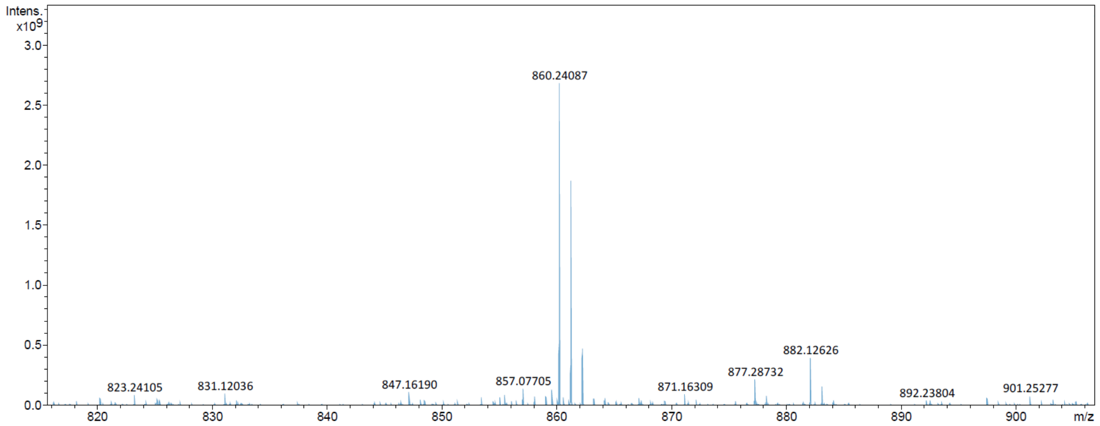

Meas. m/z	#	Ion Formula	Score	m/z	err [ppm]	Mean err [ppm]	mSigma	rdb	e⁻ Conf	N-Rule
860.240873	1	C51H45N4ORhSi	100.00	860.241219	0.4	-1.4	45.6	33.0	odd	ok

Supplementary Figure 6 HR-ESI-MS spectrum of **6a.**

400 μL of THF saturated by **6a**·THF was layered on 1.0 mL of water. The solution was stored at room temperature and red crystals were collected. The single-crystal X-ray diffraction data was collected on a Rigaku MM007HF Saturn724+ diffractometer. The structure was solved and refined using SHELXL-97. Crystals of **6a**·THF was grown in the same way as that for **6a**·THF. The crystallographic data for **6a** are listed in **Supplementary Table 2**.

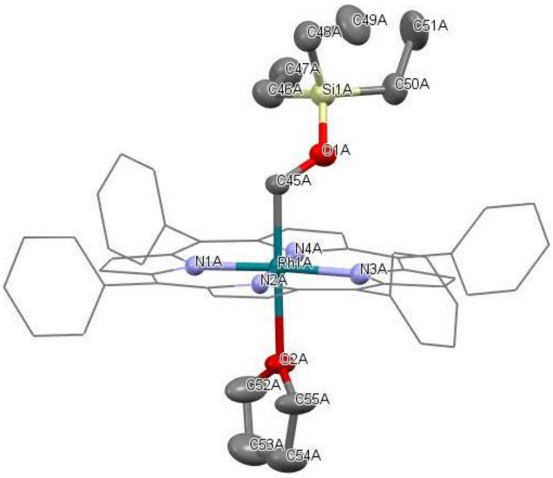

Supplementary Figure 7 X-ray crystal structure presentation of the molecular structure for **6a·THF**. Thermal ellipsoids set at 50% probability; hydrogen atoms and some labels are omitted for clarity. Gray: carbon, yellow: silicon, red: oxygen, light blue: nitrogen, dark blue-green: rhodium.

Supplementary Table 2 Crystal data and structure refinement for **6a·THF**.

Empirical formula	C55 H53 N4 O2 Rh Si	
Formula weight	933.01	
Temperature	173.1500 K	
Wavelength	0.71073 Å	
Crystal system	Triclinic	
Space group	P -1	
Unit cell dimensions	a = 13.287(3) Å	α = 75.999(6)°.
	b = 18.207(3) Å	β = 89.444(7)°.
	c = 19.784(4) Å	γ = 84.548(8)°.
Volume	4622.1(16) Å^3	
Z	4	

Density (calculated)	1.341 Mg/m^3
Absorption coefficient	0.442 mm^{-1}
F(000)	1944
Crystal size	0.273 x 0.081 x 0.063 mm^3
Theta range for data collection	1.158 to 25.200°.
Index ranges	-15<=h<=15, -21<=k<=21, -23<=l<=23
Reflections collected	55894
Independent reflections	16613 [R(int) = 0.0948]
Completeness to theta = 25.200°	99.9 %
Absorption correction	Semi-empirical from equivalents
Max. and min. transmission	1.00000 and 0.72242
Refinement method	Full-matrix least-squares on F^2
Data / restraints / parameters	16613 / 18 / 1160
Goodness-of-fit on F^2	1.149
Final R indices [I>2sigma(I)]	R1 = 0.0859, wR2 = 0.1446
R indices (all data)	R1 = 0.1178, wR2 = 0.1577
Extinction coefficient	n/a
Largest diff. peak and hole	0.672 and -0.543 e.Å^{-3}

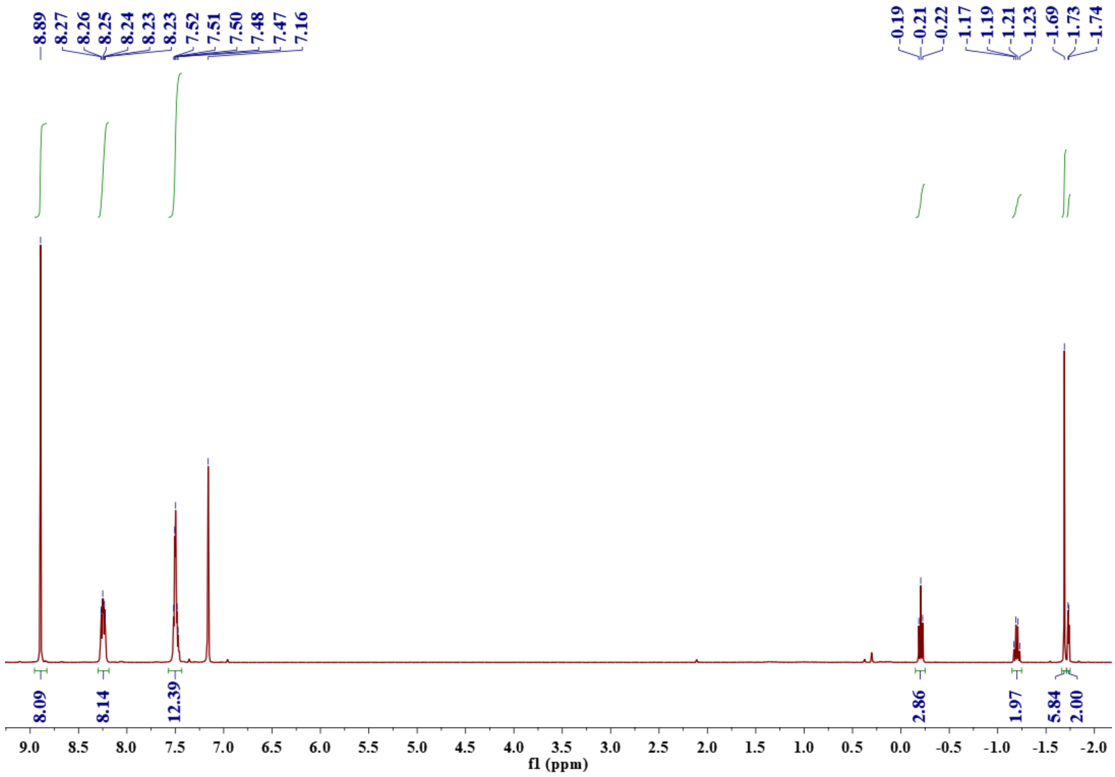

Supplementary Figure 8 ^{1}H NMR (400 MHz, C_6D_6, 25 °C) spectrum of **6b.**

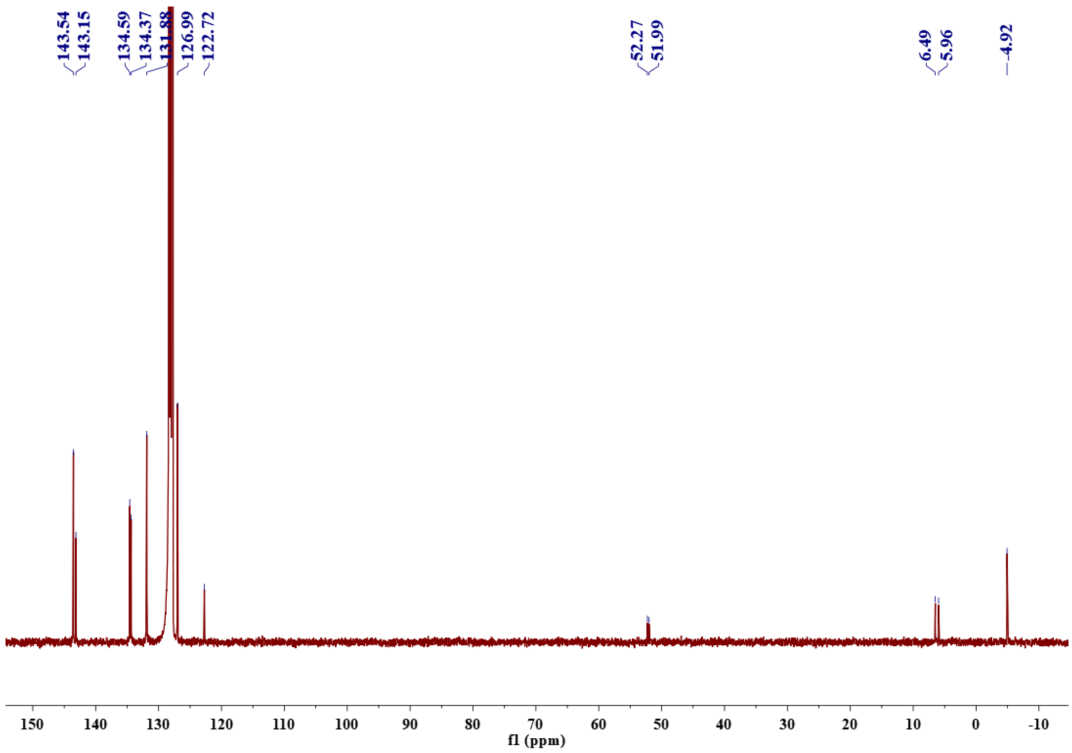

Supplementary Figure 9 ^{13}C NMR (101 MHz, C_6D_6, 25 °C) spectrum of **6b.**

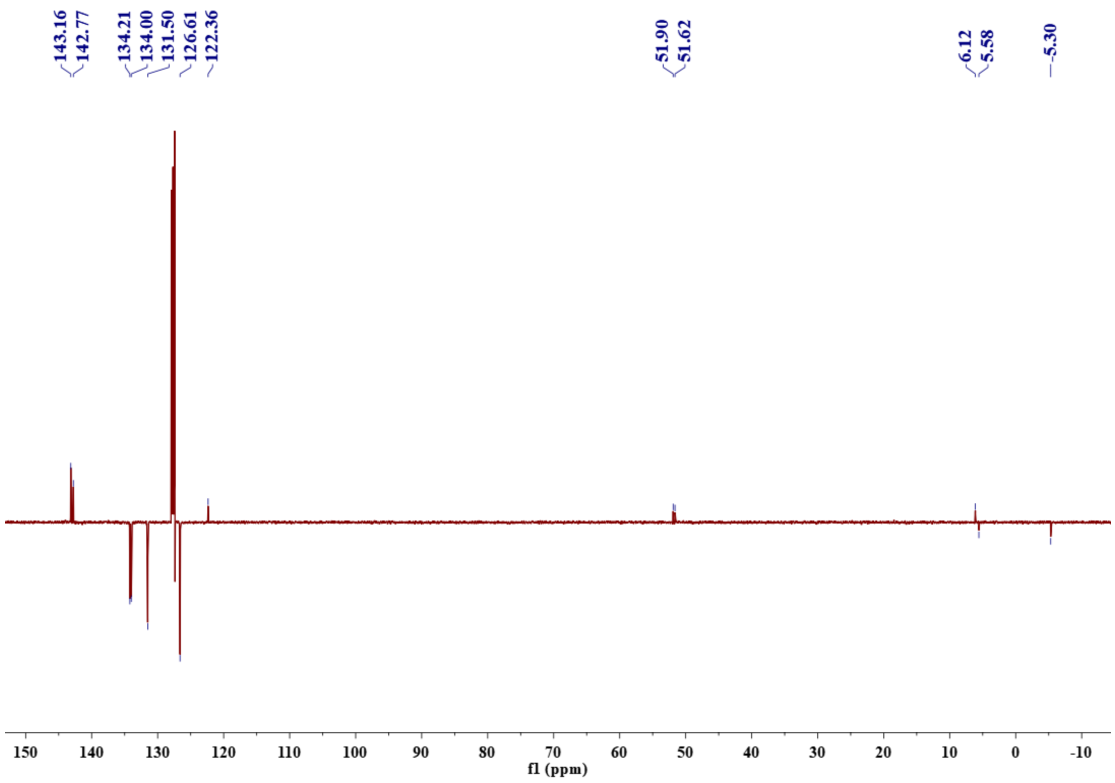

Supplementary Figure 10 ^{13}C APT NMR (101 MHz, C_6D_6, 25 °C) spectrum of **6b.**

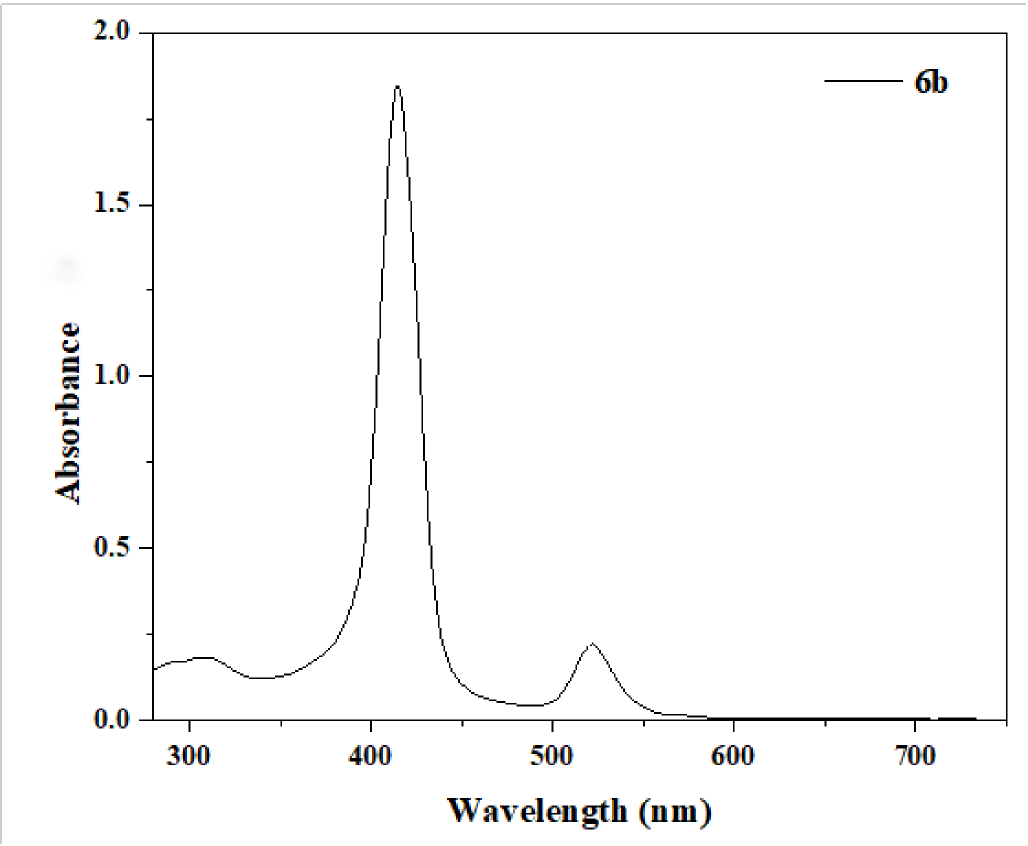

Supplementary Figure 11 UV-Vis spectrum of **6b** in toluene.

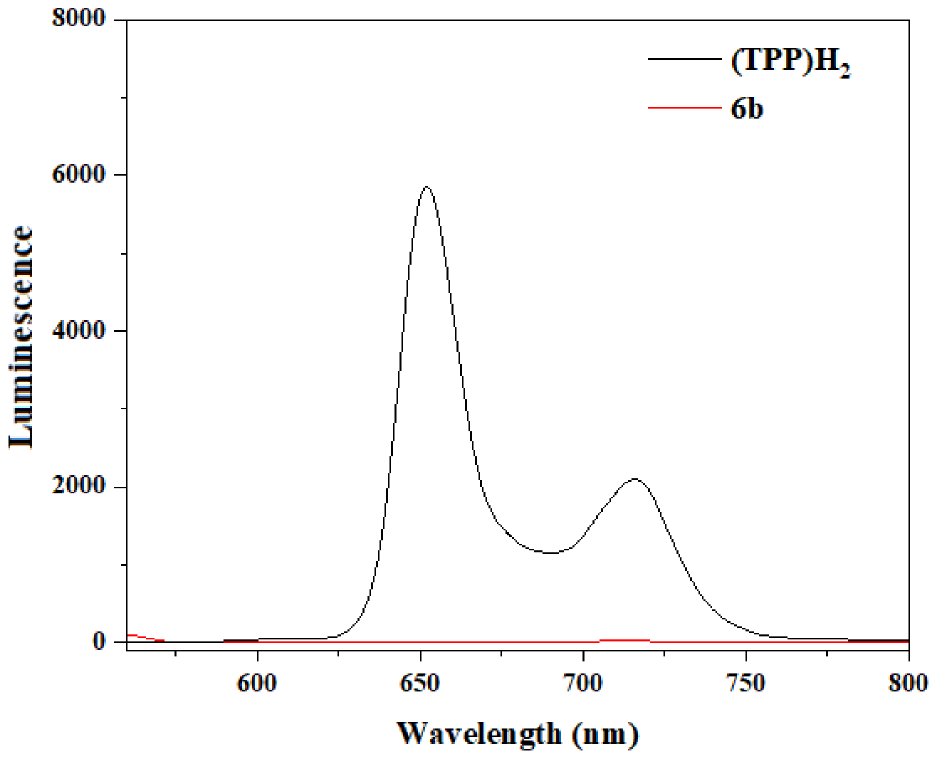

Supplementary Figure 12 Luminescence spectrum of **6b** in toluene, $(TPP)H_2$ for reference. No appreciable luminescence of **6b** was found.

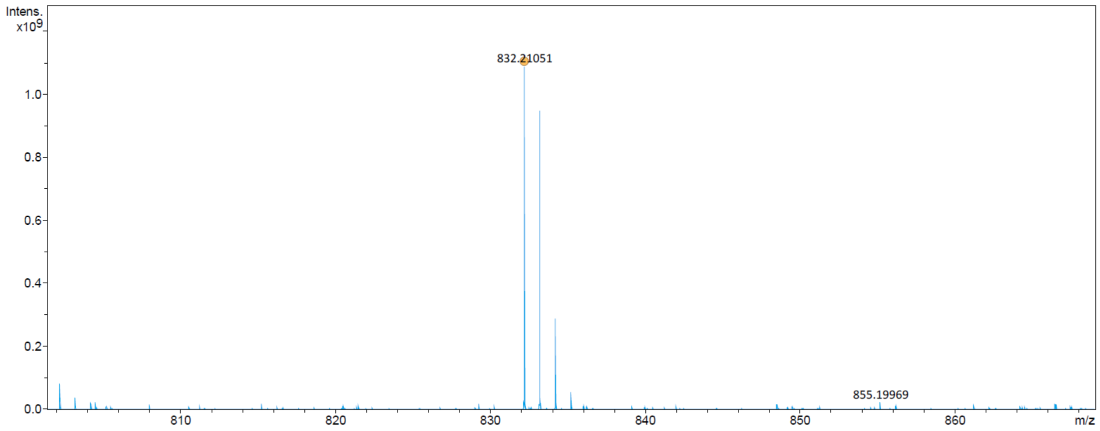

Meas. m/z	#	Ion Formula	Score	m/z	err [ppm]	Mean err [ppm]	mSigma	rdb	e⁻ Conf	N-Rule
832.210514	1	C49H41N4ORhSi	100.00	832.209919	-0.7	614.7	123.3	33.0	odd	ok

Supplementary Figure 13 HR-ESI-MS spectrum of **6b.**

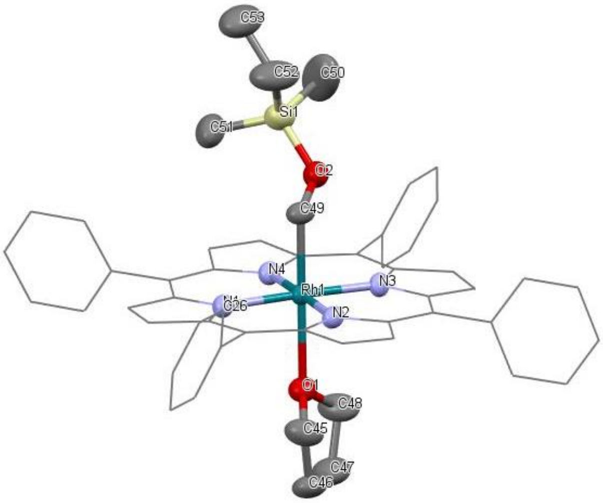

Supplementary Figure 14 X-ray crystal structure presentation of the molecular structure for **6b·THF**. Thermal ellipsoids set at 50% probability; hydrogen atoms and some labels are omitted for clarity.

Supplementary Table 3 Crystal data and structure refinement for **6b·THF**.

Empirical formula	C53 H49 N4 O2 Rh Si	
Formula weight	904.96	
Temperature	173.1500 K	
Wavelength	0.71073 Å	
Crystal system	Triclinic	
Space group	P -1	
Unit cell dimensions	a = 11.5475(11) Å	α = 88.820(7)°.
	b = 12.1867(14) Å	β = 71.395(5)°.

	c = 18.022(2) Å	γ = 67.880(5)°.
Volume	2212.3(4) Å^3	
Z	2	
Density (calculated)	1.359 Mg/m^3	
Absorption coefficient	0.460 mm^{-1}	
F(000)	940	
Crystal size	0.24 x 0.21 x 0.06 mm^3	
Theta range for data collection	1.815 to 27.481°.	
Index ranges	-14<=h<=14, -15<=k<=15, -23<=l<=23	
Reflections collected	27297	
Independent reflections	10089 [R(int) = 0.0434]	
Completeness to theta = 26.000°	99.7 %	
Absorption correction	Semi-empirical from equivalents	
Max. and min. transmission	1.0000 and 0.7984	
Refinement method	Full-matrix least-squares on F^2	
Data / restraints / parameters	10089 / 69 / 582	
Goodness-of-fit on F^2	1.081	
Final R indices [I>2sigma(I)]	R1 = 0.0641, wR2 = 0.1605	
R indices (all data)	R1 = 0.0673, wR2 = 0.1633	
Extinction coefficient	n/a	
Largest diff. peak and hole	2.251 and -1.410 e.Å^{-3}	

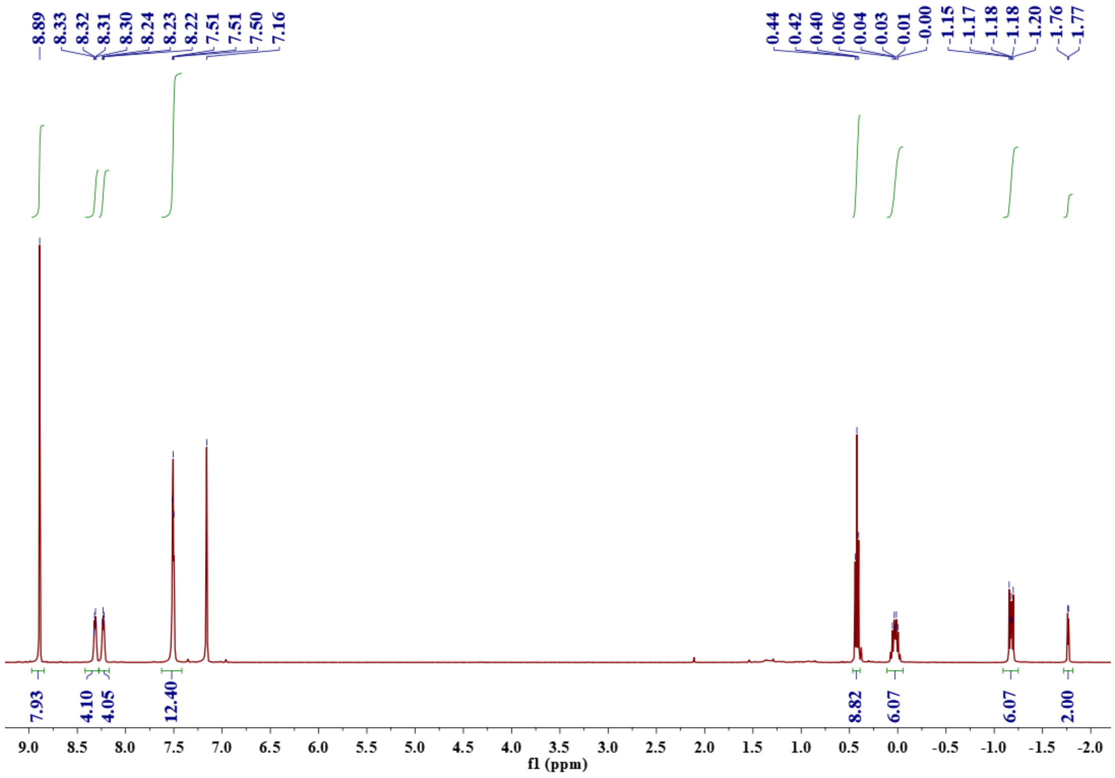

Supplementary Figure 15 ^{1}H NMR (400 MHz, C_6D_6, 25 °C) spectrum of **6c.**

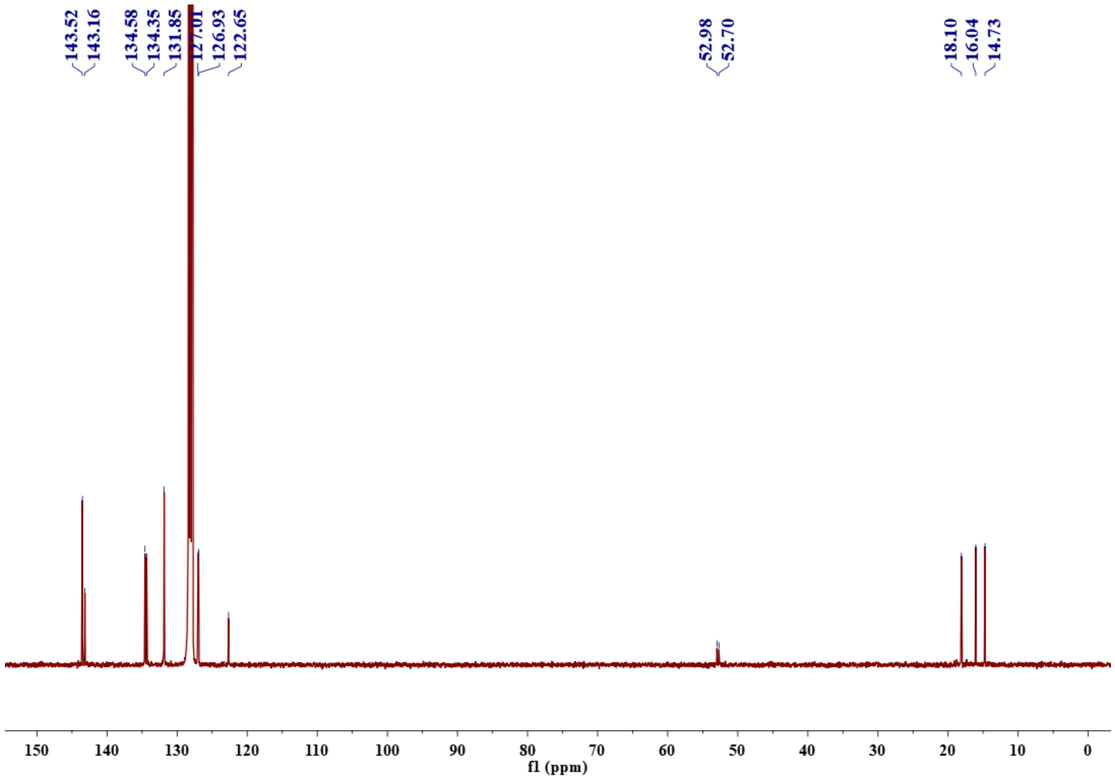

Supplementary Figure 16 ^{13}C NMR (101 MHz, C_6D_6, 25 °C) spectrum of **6c.**

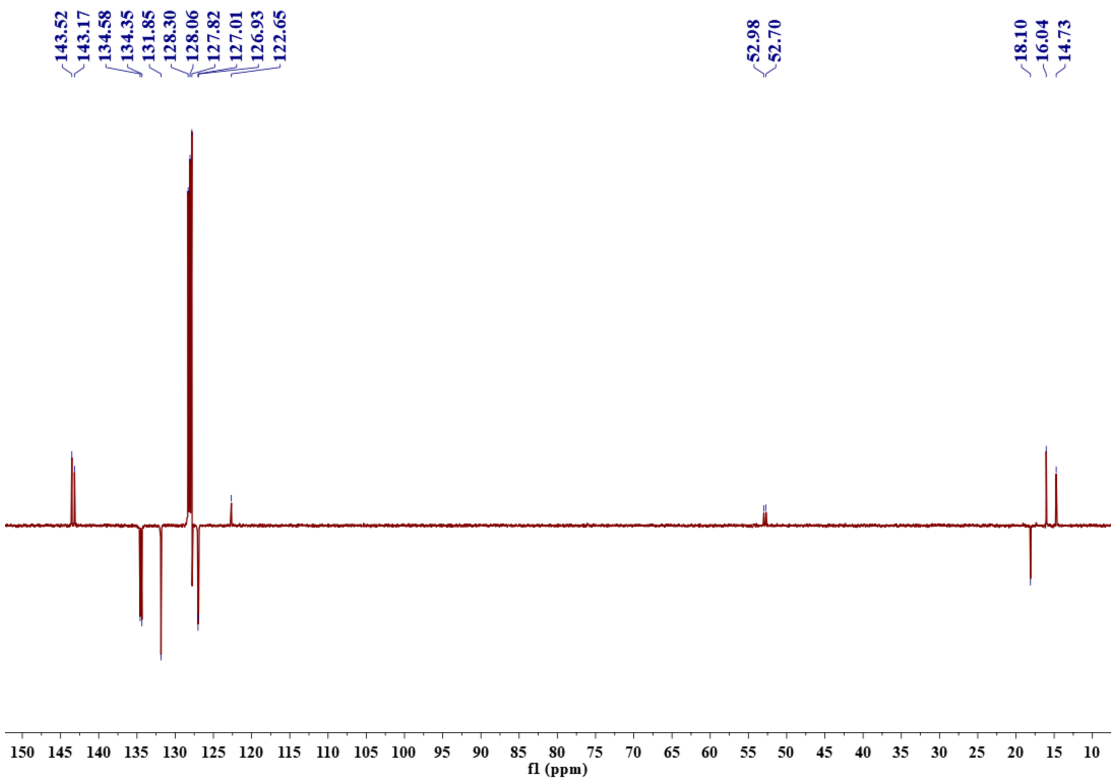

Supplementary Figure 17 ^{13}C APT NMR (101 MHz, C_6D_6, 25 °C) spectrum of **6c.**

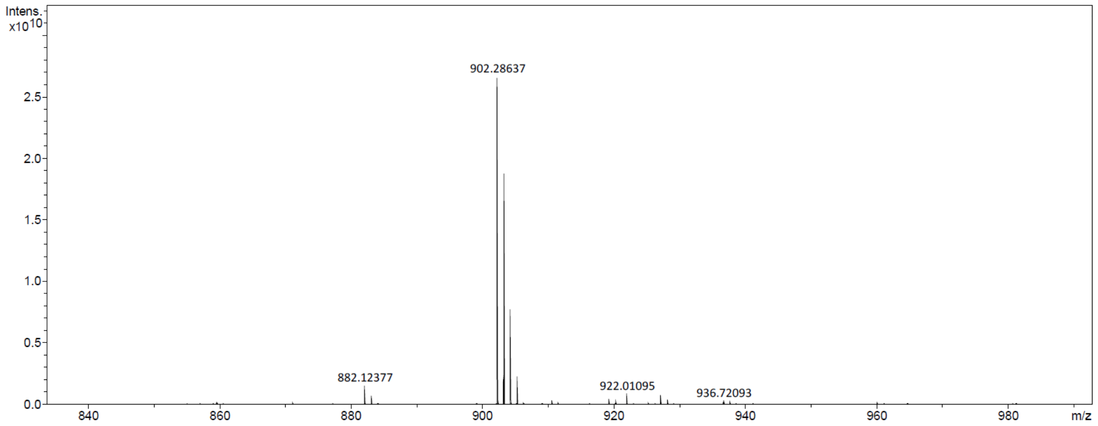

Meas. m/z	#	Ion Formula	Score	m/z	err [ppm]	Mean err [ppm]	mSigma	rdb	e⁻ Conf	N-Rule
902.286367	1	C54H51N4ORhSi	100.00	902.288169	2.0	0.2	31.4	33.0	odd	ok

Supplementary Figure 18 HR-ESI-MS spectrum of **6c.**

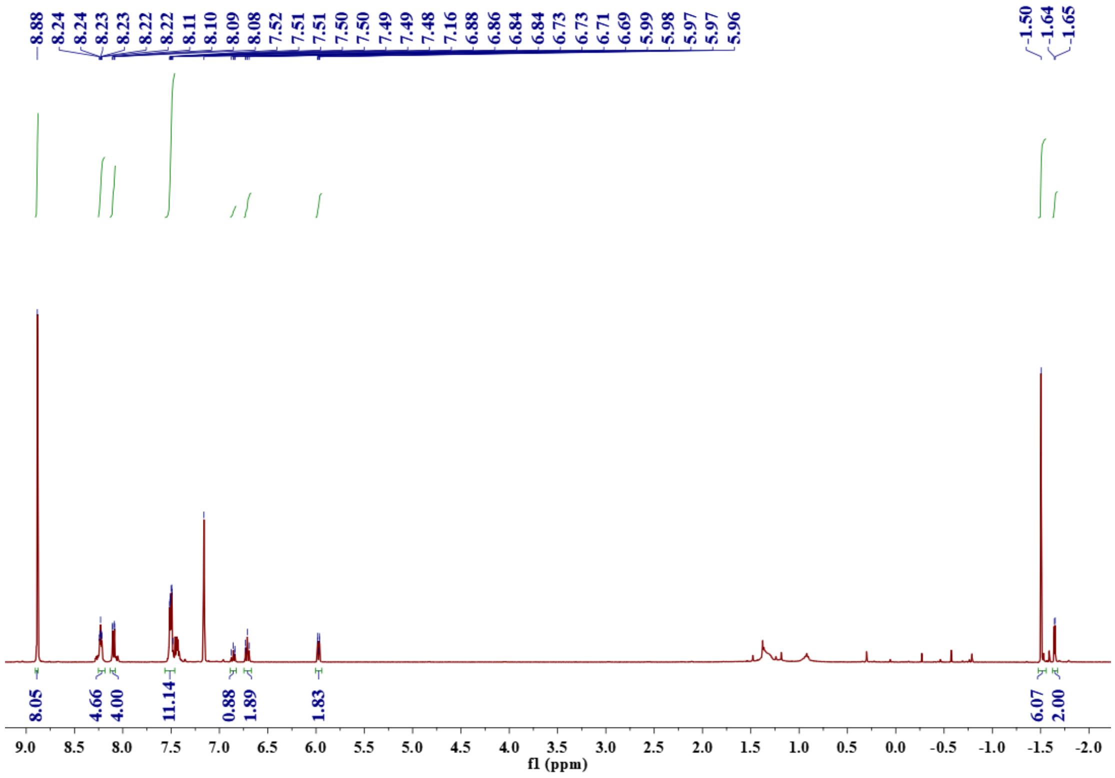

Supplementary Figure 19 ^{1}H NMR (400 MHz, C_6D_6, 25 ℃) spectrum of **6d.**

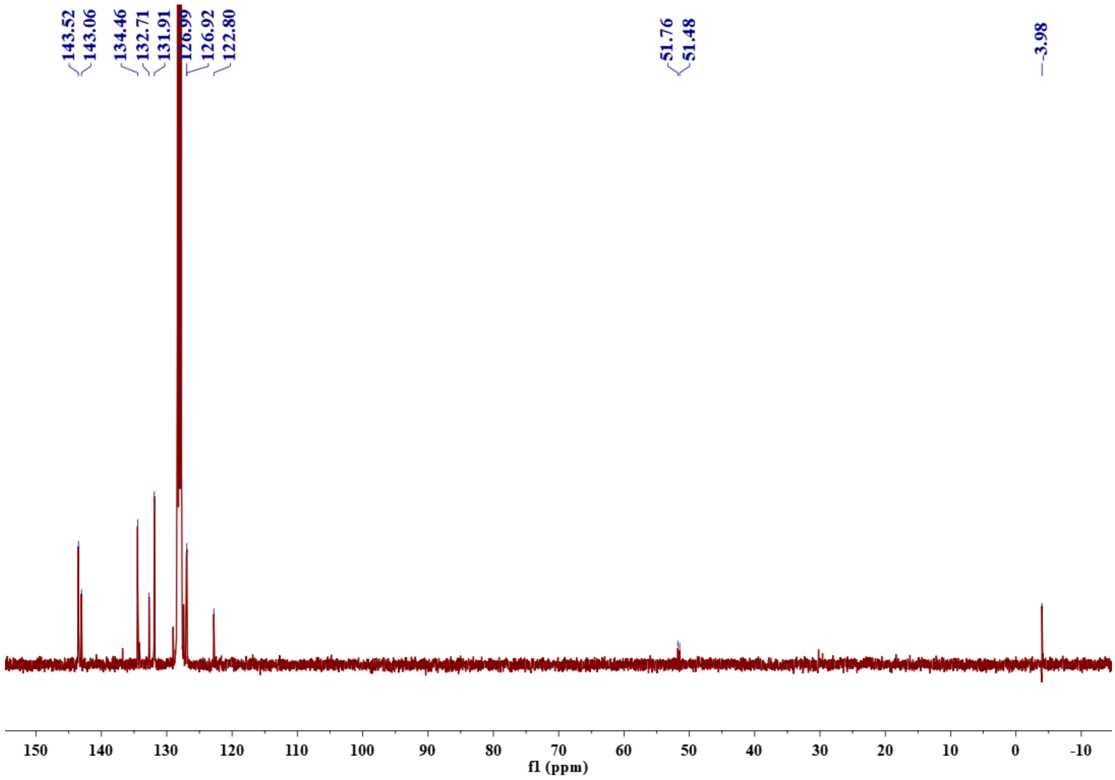

Supplementary Figure 20 ^{13}C NMR (101 MHz, C_6D_6, 25 ℃) spectrum of **6d.**

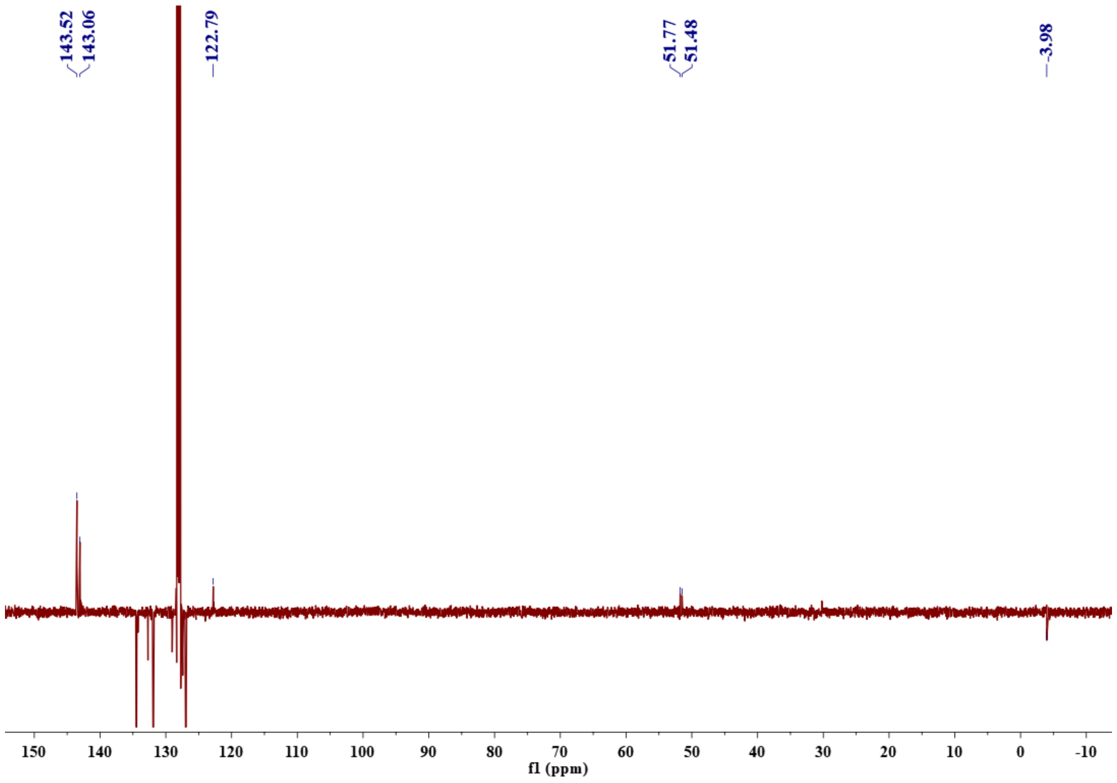

Supplementary Figure 21 ^{13}C APT NMR (101 MHz, C_6D_6, 25 °C) spectrum of **6d.**

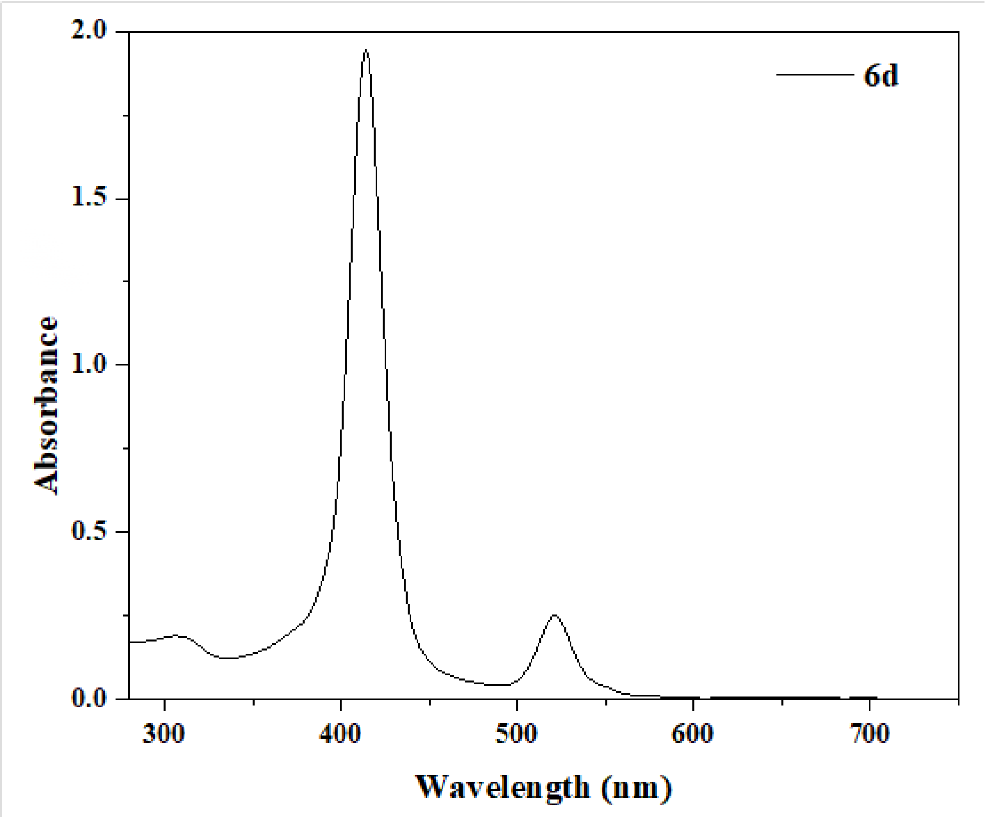

Supplementary Figure 22 UV-Vis spectrum of **6d** in toluene.

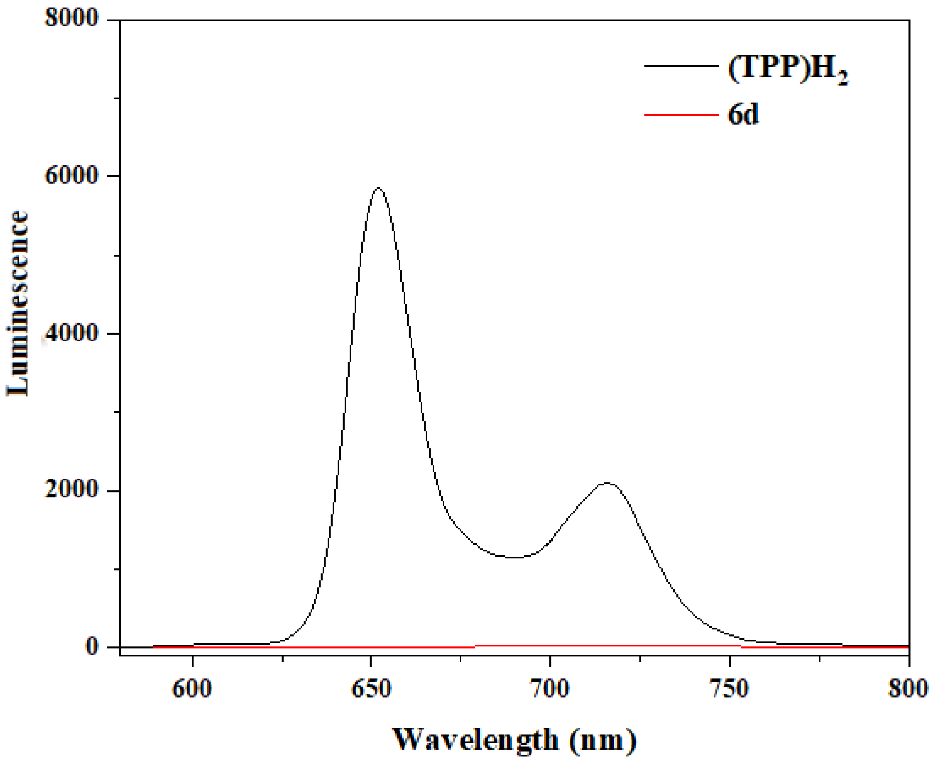

Supplementary Figure 23 Luminescence spectrum of **6d** in toluene, (TPP)H_2 for reference. No appreciable luminescence of **6d** was found.

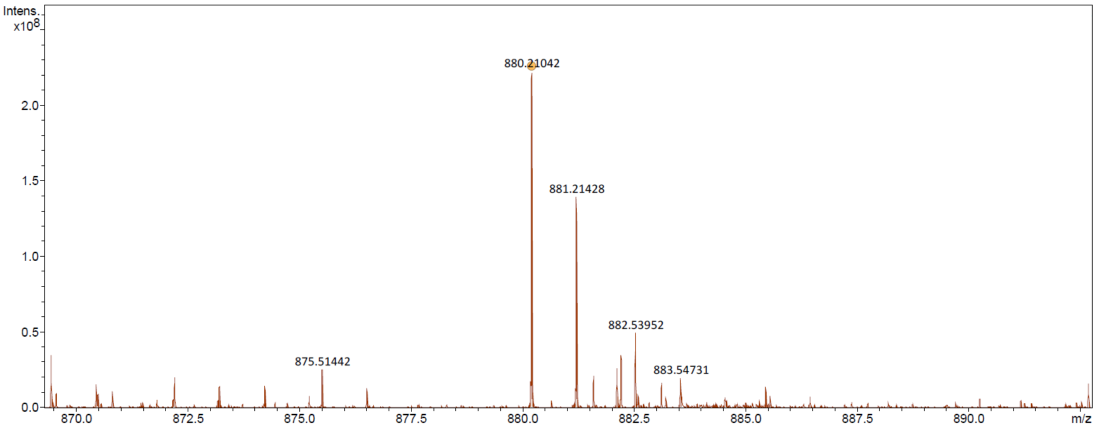

Meas. m/z	#	Ion Formula	Score	m/z	err [ppm]	Mean err [ppm]	mSigma	rdb	e⁻ Conf	N-Rule
880.210425	1	C53H41N4ORhSi	100.00	880.209919	-0.6	1031.8	46.4	37.0	odd	ok

Supplementary Figure 24 HR-ESI-MS spectrum of **6d.**

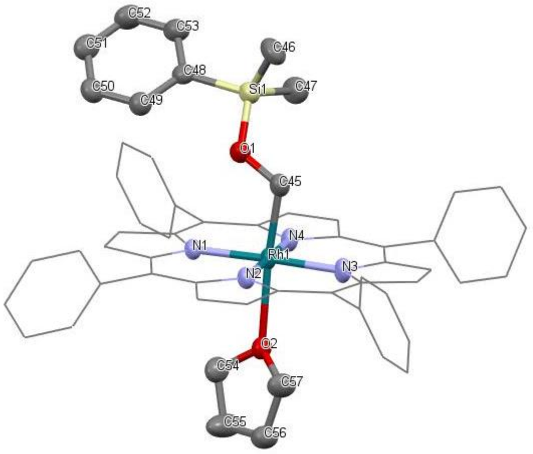

Supplementary Figure 25 X-ray crystal structure presentation of the molecular structure for **6d·THF**. Thermal ellipsoids set at 50% probability; hydrogen atoms and some labels are omitted for clarity. Gray: carbon, yellow: silicon, red: oxygen, light blue: nitrogen, dark blue-green: rhodium.

Supplementary Table 4 Crystal data and structure refinement for **6d·THF**.

Empirical formula	C57 H49 N4 O2 Rh Si	
Formula weight	953.00	
Temperature	173.1500 K	
Wavelength	0.71073 Å	
Crystal system	Triclinic	
Space group	P -1	
Unit cell dimensions	a = 9.7441(19) Å	α = 86.084(7)°.
	b = 11.903(2) Å	β = 86.851(7)°.
	c = 22.094(5) Å	γ = 76.534(6)°.
Volume	2484.3(9) $Å^3$	
Z	2	
Density (calculated)	1.274 Mg/m^3	
Absorption coefficient	0.413 mm^{-1}	

F(000)	988
Crystal size	0.42 x 0.12 x 0.06 mm^3
Theta range for data collection	0.925 to 27.477°.
Index ranges	-12<=h<=12, -15<=k<=15, -28<=l<=28
Reflections collected	32631
Independent reflections	11382 [R(int) = 0.0485]
Completeness to theta = 26.000°	99.8 %
Absorption correction	Semi-empirical from equivalents
Max. and min. transmission	1.0000 and 0.7914
Refinement method	Full-matrix least-squares on F^2
Data / restraints / parameters	11382 / 0 / 588
Goodness-of-fit on F^2	1.102
Final R indices [I>2sigma(I)]	R1 = 0.0518, wR2 = 0.1138
R indices (all data)	R1 = 0.0568, wR2 = 0.1172
Extinction coefficient	n/a
Largest diff. peak and hole	0.522 and -0.608 e.$Å^{-3}$

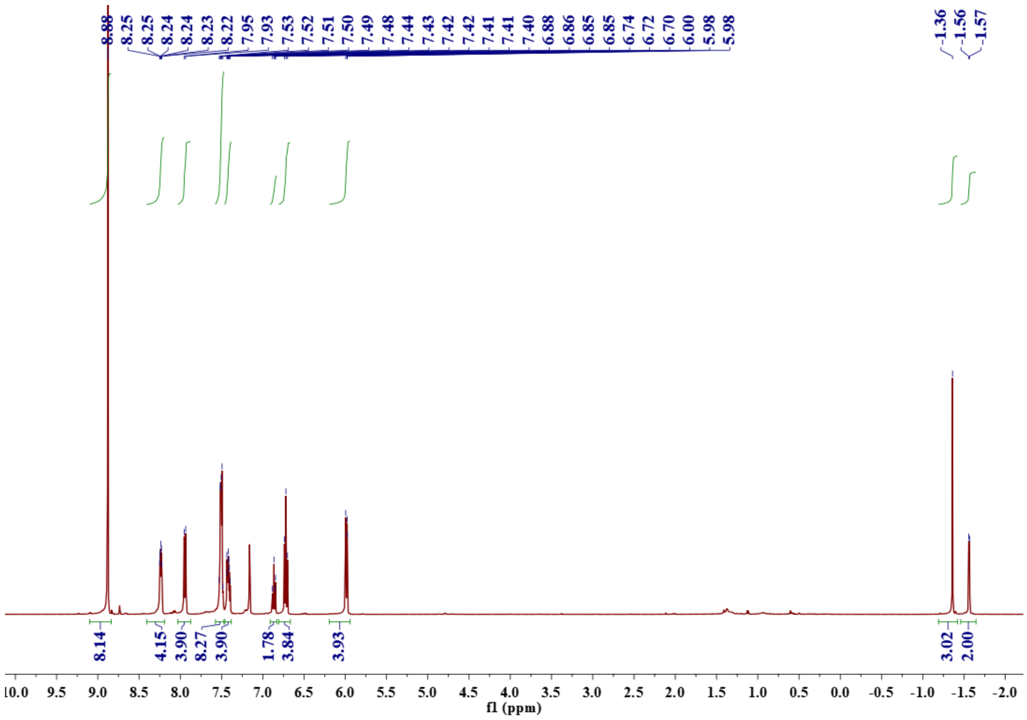

Supplementary Figure 26 ^{1}H NMR (400 MHz, C_6D_6, 25 °C) spectrum of **6e.**

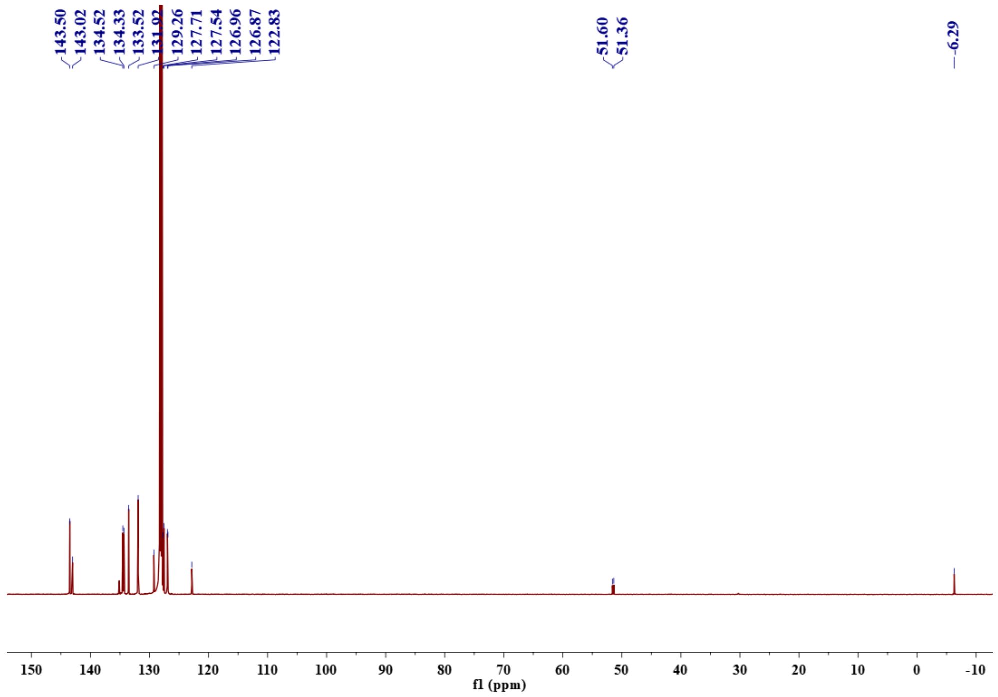

Supplementary Figure 27 ^{13}C NMR (101 MHz, C_6D_6, 25 °C) spectrum of **6e.**

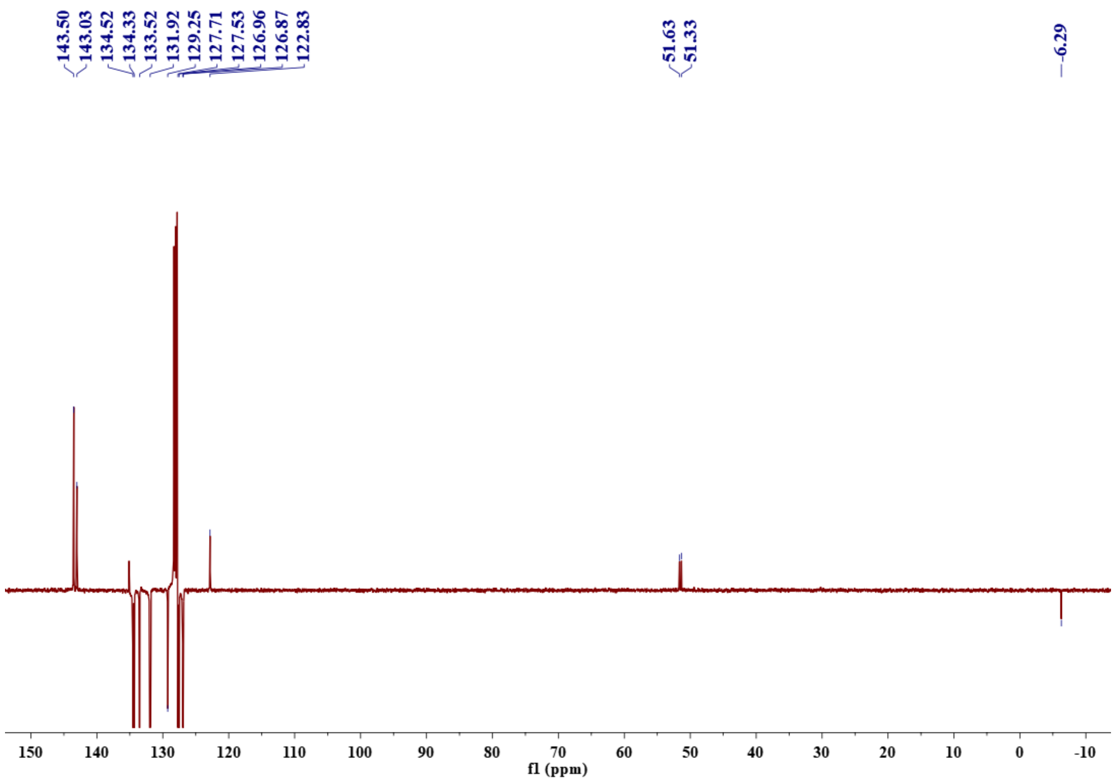

Supplementary Figure 28 ^{13}C APT NMR (101 MHz, C_6D_6, 25 °C) spectrum of **6e.**

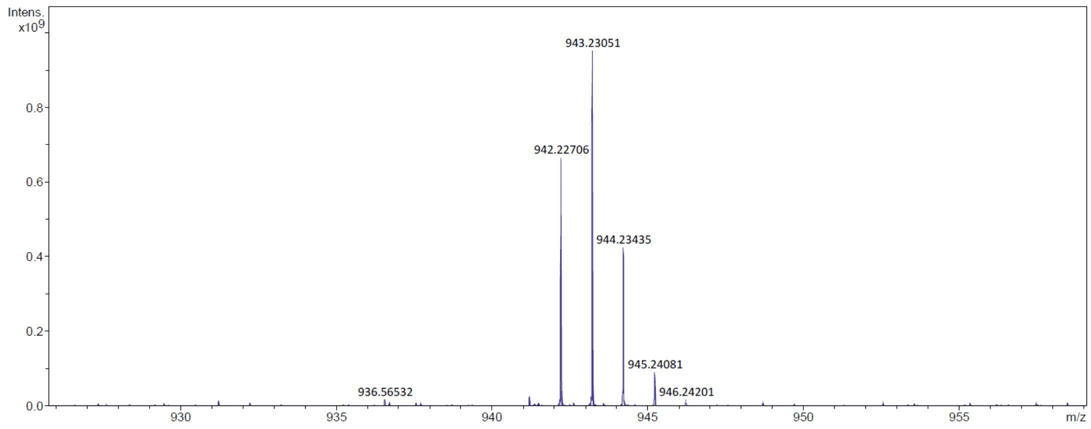

Meas. m/z	#	Ion Formula	Score	m/z	err [ppm]	Mean err [ppm]	mSigma	rdb	e⁻ Conf	N-Rule
942.227056	1	C58H43N4ORhSi	100.00	942.225569	-1.6	-4.1	205.6	41.0	odd	ok

Supplementary Figure 29 HR-ESI-MS spectrum of **6e.**

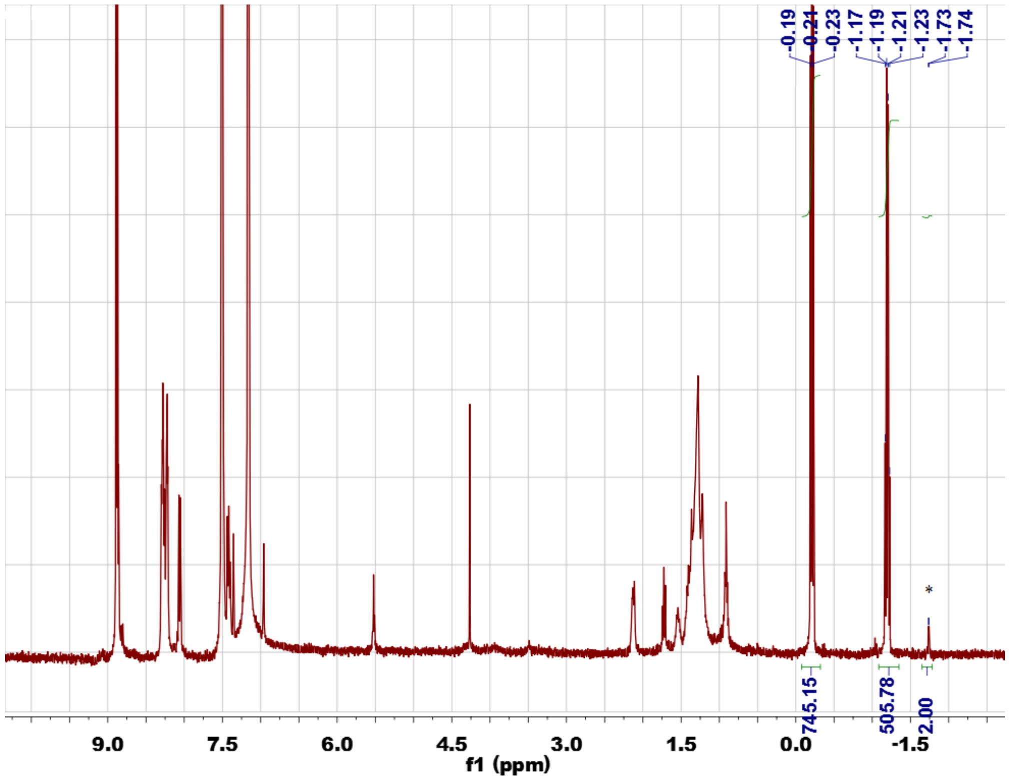

Supplementary Figure 30 ^{1}H NMR (400 MHz, C_6D_6, 25 °C) spectrum of **6f.** "*" at -1.73 ppm represents the residual of (TPP)RhCH_2OSiEt$_3$ in C_6D_6.

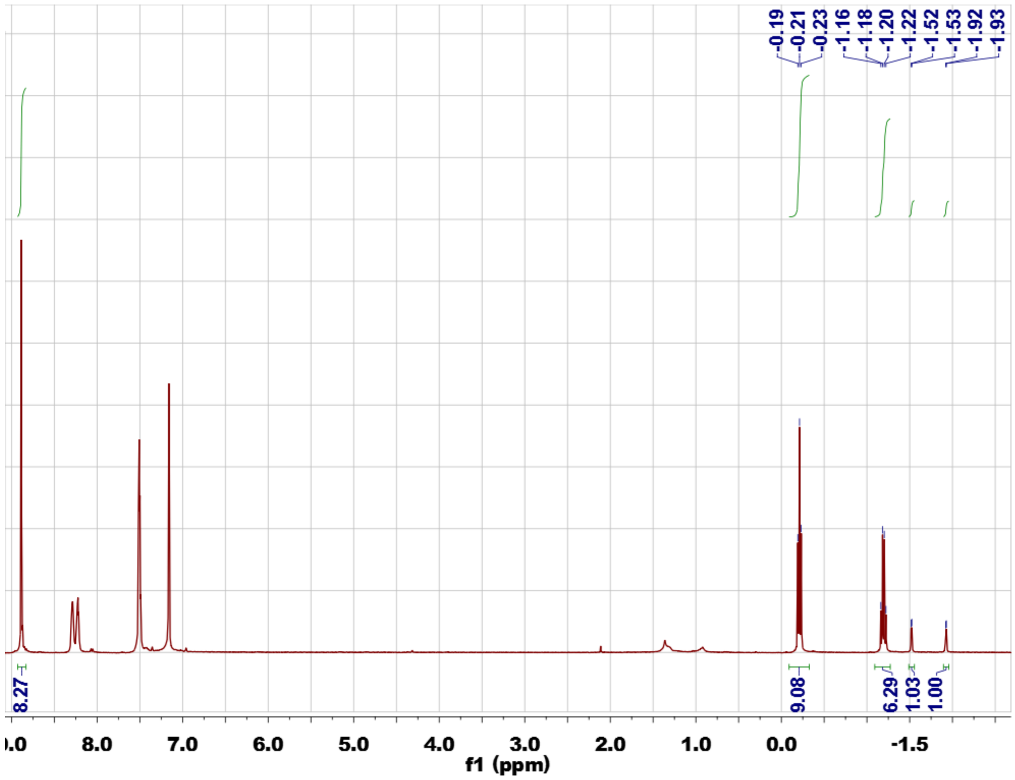

Supplementary Figure 31 ^{1}H NMR (400 MHz, C_6D_6, 25 °C) spectrum of **6g.**

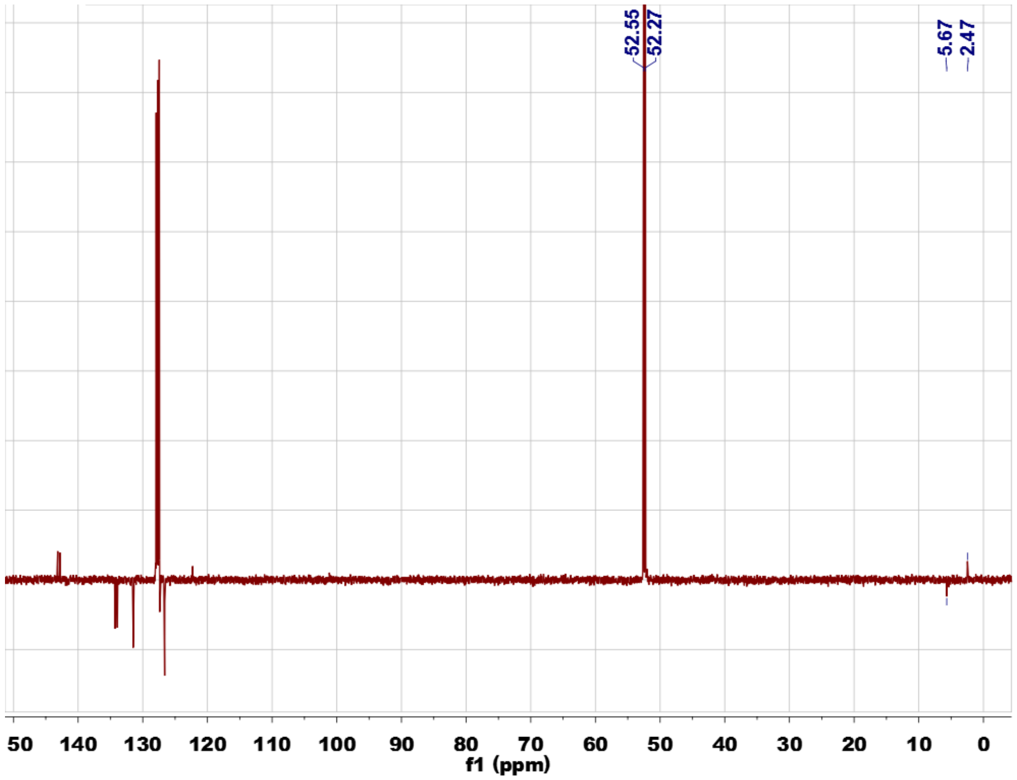

Supplementary Figure 32 ^{13}C APT NMR (101 MHz, C_6D_6, 25 °C) spectrum of **6g.**

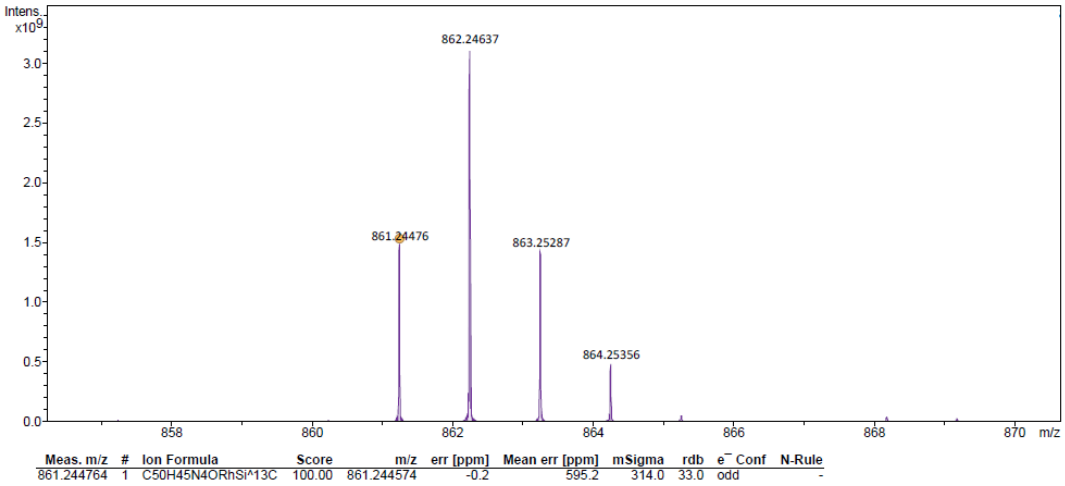

Supplementary Figure 33 HR-ESI-MS spectrum of **6g.**

Release products and characterization of $XCH_2OSiR^1R^2R^3$(X = TEMPO, H, Br, D)

0.7 mg of (TPP)$RhCH_2OSiR^1R^2R^3$ and excess TEMPO (over 10 eq.) were added into a vacuum adapted NMR tube with 350 μL of deuterated benzene. After three freeze-pump-thaw

cycles, the NMR tube was exposed to visible light in ethanol bath at 6ºC for 1 hour which afforded a dark orange red solution containing TEMPO-Rh(TPP) ($[M]^+$, *m/z* = 872.28564) and TEMPO-$CH_2OSi(CH_2CH_3)_3$ (**9**) ($[M+H]^+$, *m/z* = 302.25170).

0.7 mg of (TPP)$RhCH_2OSiR^1R^2R^3$ and 2 μL of silane or 1.5 mg of (TPP)RhH were added into a vacuum adapted NMR tube with 350 μL of deuterated benzene. After three freeze-pump-thaw cycles, the NMR tube was exposed to visible light in ethanol bath at 6ºC for 1 hour which afforded a dark orange red solution containing (TPP)$RhSiMePh_2$ and $CH_3OSiR^1R^2R^3$. The yields are shown in **Supplementary Table 1**.

In order to confirm the source of hydrogen, we synthesized $(CH_3CH_2)_3SiD$ (60 % D) according to published procedure.[10] Addition of this partially deuterated silane to (TPP)$RhCH_2OSi(CH_2CH_3)_3$ followed by irradiation for 2 hours at 6 ºC produced $DCH_2OSi(CH_2CH_3)_3$ (56 % D).

0.7 mg of (TPP)$RhCH_2OSiR^1R^2R^3$ and 2 μL of $BrCCl_3$ were added into a vacuum adapted NMR tube with 350 μL of deuterated benzene. After three freeze-pump-thaw cycles, the NMR tube was exposed to visible light in alcohol bath at 6ºC for 1 hour, affording a dark orange red solution containing (TPP)RhBr and $BrCH_2OSiR^1R^2R^3$.

CH_3OSiEt_3 (7a): ^{1}H NMR (400 MHz, C_6D_6) δ (ppm): 3.31 (s, 3 H, C$\underline{H}_3$OSi-), 0.99 (t, 9 H, $^3J_{H\text{-}H}$ = 8.0 Hz, -$SiCH_2C\underline{H}_3$), 0.58 (q, 6 H, $^3J_{H\text{-}H}$ = 8.0 Hz, -$SiC\underline{H}_2CH_3$); ^{13}C NMR (DEPT-135, 100 MHz, C_6D_6) δ (ppm): 50.08 (s, $\underline{C}H_3$OSi-), 6.66 (s, Si-$CH_2\underline{C}H_3$), 4.21 (s, Si-$\underline{C}H_2CH_3$). GC-MS m/z calcd for $C_7H_{18}OSi$, $[M]^+$ 146.1; found 146.1.

CH_3OSiMe_2Et (7b): ^{1}H NMR (500 MHz, C_6D_6) δ (ppm): 3.27 (s, 3 H, C$\underline{H}_3$OSi-), 0.97 (t, 3 H, $^3J_{H\text{-}H}$ = 7.9 Hz, -$SiCH_2C\underline{H}_3$), 0.54 (q, 2 H, $^3J_{H\text{-}H}$ = 8.0 Hz, -$SiC\underline{H}_2CH_3$), 0.05 (s, 6 H, Si-C$\underline{H}_3$); ^{13}C NMR (DEPT-135, 126 MHz, C_6D_6) δ (ppm): 50.02 (s, $\underline{C}H_3$OSi-), 8.06 (s, Si-$CH_2\underline{C}H_3$), 6.96 (s, Si-$\underline{C}H_2CH_3$), -3.02 (s, Si-C$\underline{H}_3$).

$CH_3OSi^nPr_3$ (7c): ^{1}H NMR (400 MHz, C_6D_6) δ (ppm): 3.32 (s, 3 H, C$\underline{H}_3$OSi-), 1.43 (m, 6 H, Si-$CH_2C\underline{H}_2CH_3$), 1.01 (t, 9 H, $^3J_{H\text{-}H}$ = 8.0 Hz, Si-$CH_2CH_2C\underline{H}_3$), 0.61 (m, 6 H, Si-$C\underline{H}_2CH_2CH_3$); ^{13}C NMR (DEPT-135, 126 MHz, C_6D_6) δ (ppm): 50.07 (s, $\underline{C}H_3$OSi-),18.22 (s, Si- $CH_2CH_2\underline{C}H_3$),

16.84 (s, Si- $CH_2\underline{C}H_2CH_3$), 16.15 (s, Si-$\underline{C}H_2CH_2CH_3$). GC-MS m/z calcd for $C_{10}H_{24}OSi$, $[M]^+$ 188.2; found 146.2.

CH_3OSiMe_2Ph (7d): ^1H NMR (400 MHz, C_6D_6) δ (ppm): 7.56 (m, 2 H, Si-phenyl), 7.22 (m, 2 H, Si-phenyl), 6.15 (m, 1 H, Si-phenyl), 3.28 (s, 3 H, $C\underline{H_3}OSi$-), 0.30 (s, 6 H, -$SiC\underline{H_3}$). HR-ESI-MS m/z calcd for $C_9H_{14}OSi$, $[M]^+$ 166.08139, found 167.08808.

$CH_3OSiMePh_2$ (7e): ^1H NMR (400 MHz, C_6D_6) δ (ppm): 3.37 (s, 3 H, $C\underline{H_3}OSi$-), 0.30 (s, 3 H, -$SiC\underline{H_3}$); ^{13}C NMR (DEPT-135, 100 MHz, C_6D_6) δ (ppm): 59.23 ($\underline{C}H_3OSi$-), 3.43 (Si-$\underline{C}H_3$).

DCH_2OSiEt_3 (7f): ^1H NMR (400 MHz, C_6D_6) δ (ppm): 3.28 (s, 2 H, $^2J_{D\text{-}H}$ = 1.6 Hz, $DC\underline{H_2}OSi$-), 0.94 (t, 9 H, $^3J_{H\text{-}H}$ = 8.0 Hz, -$SiCH_2C\underline{H_3}$), 0.49 (q, 6 H, $^3J_{H\text{-}H}$ = 8.0 Hz, -$SiC\underline{H_2}CH_3$).

$BrCH_2OSiEt_3$ (8): ^1H NMR (400 MHz, C_6D_6) δ (ppm): 5.41 (s, 2 H, $BrC\underline{H_2}OSi$-), 0.94 (t, 9 H, $^3J_{H\text{-}H}$ = 8.0 Hz, -$SiCH_2C\underline{H_3}$), 0.54 (q, 6 H, $^3J_{H\text{-}H}$ = 8.0 Hz, -$SiC\underline{H_2}CH_3$).

(TPP)RhBr (2): ^1H NMR (400 MHz, C_6D_6) δ (ppm): 9.04 (s, 8 H, pyrrole), 8.11 (m, 4 H, *o*-phenyl), 7.94 (m, 4 H, *o'*-phenyl), 7.44-7.36 (m, 12 H, *m*-phenyl, *p*-phenyl); HR-ESI-MS m/z calcd for $[M+H]^+$ $C_{44}H_{29}N_4RhBr$ 795.06191; found 795.06191.

(TPP)RhI (1): ^1H NMR (400 MHz, C_6D_6) δ (ppm): 8.94 (s, 8 H, pyrrole), 8.26 (d, 4 H, *o*-phenyl), 8.01 (d, 4 H, *o'*-phenyl), 7.51 (m, 8 H, *m*-phenyl), 7.35 (m, 4 H, *p*-phenyl). UV-Vis: λ_{abs} (nm) (toluene) 374, 430, 540, 570. HR-ESI-MS m/z calcd for $[M+H]^+$ $C_{44}H_{29}N_4RhI$ 843.04865; found 843.04844.

Summary for spectroscopy

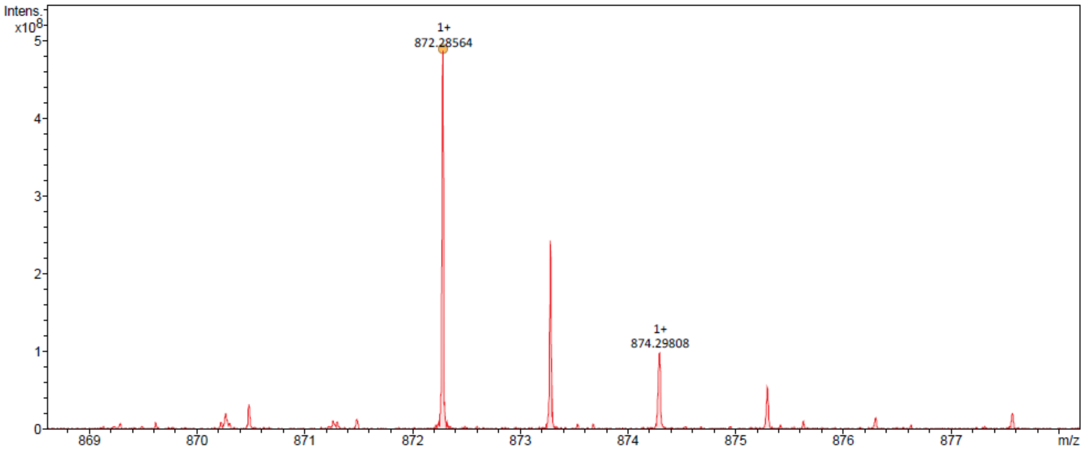

Meas. m/z	#	Ion Formula	Score	m/z	err [ppm]	Mean err [ppm]	mSigma	rdb	e⁻ Conf	N-Rule
872.285640	1	C53H47N5ORh	100.00	872.283017	-3.0	-3.0	299.5	34.0	even	ok

Supplementary Figure 34 HR-ESI-MS of TEMPO-Rh(TPP).

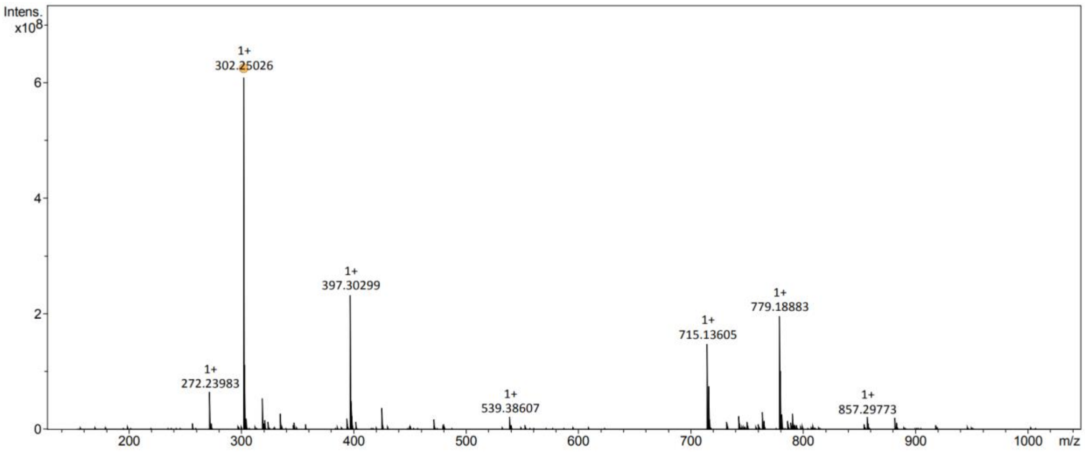

Meas. m/z	#	Ion Formula	Score	m/z	err [ppm]	Mean err [ppm]	mSigma	rdb	e⁻ Conf	N-Rule
302.250257	1	C16H36NO2Si	100.00	302.250982	2.4	1.0	4.4	1.0	even	ok

Supplementary Figure 35 HR-ESI-MS of **9.**

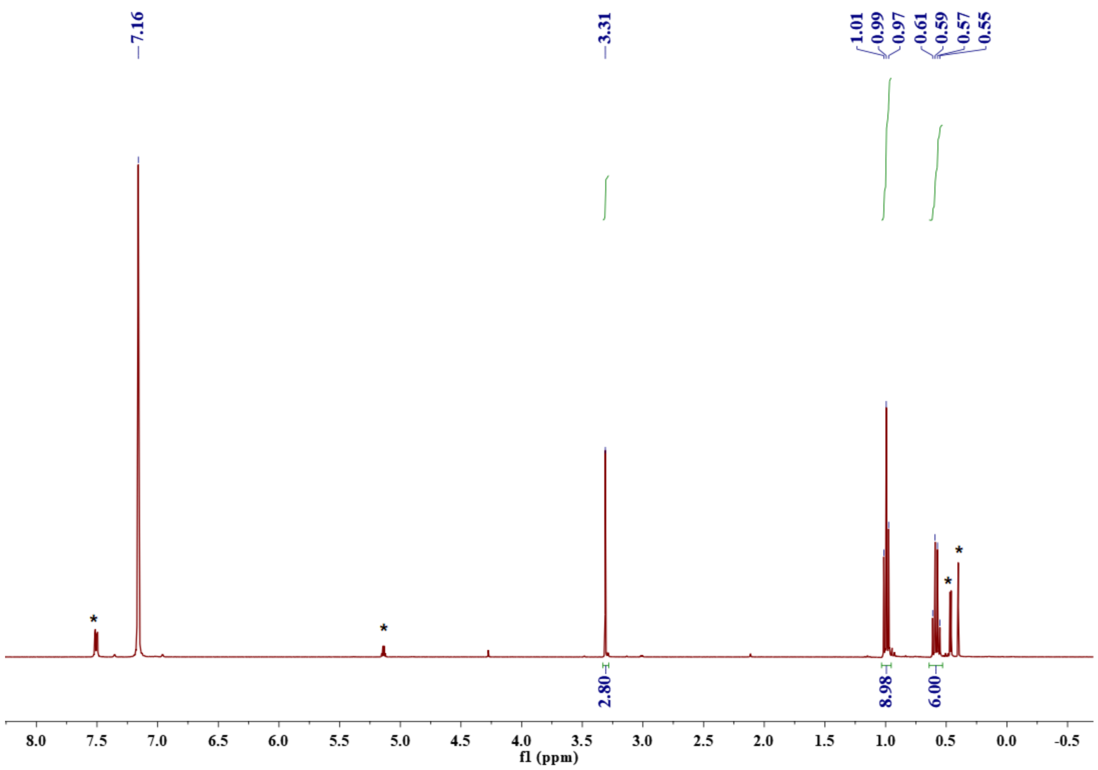

Supplementary Figure 36 ^{1}H NMR (400 MHz, C_6D_6, 25 °C) spectrum of **7a**. Signals labelled by “*” represent $HSiMePh_2$ and water.

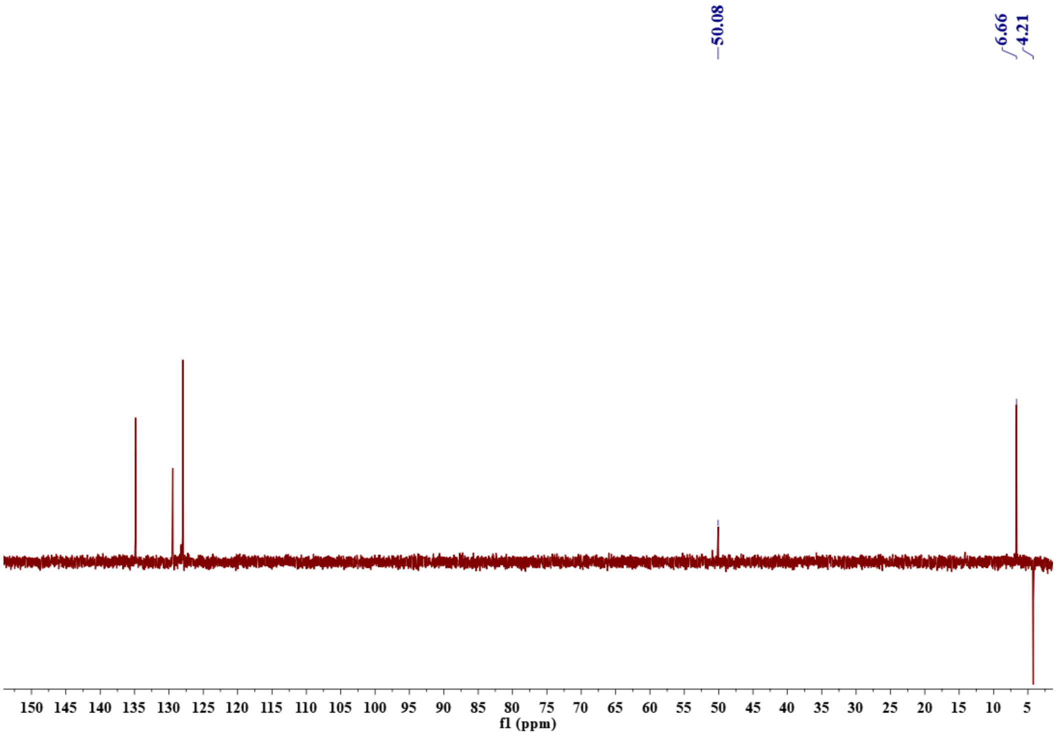

Supplementary Figure 37 ^{13}C DEPT-135 NMR (100 MHz, C_6D_6, 25 °C) spectrum of **7a.**

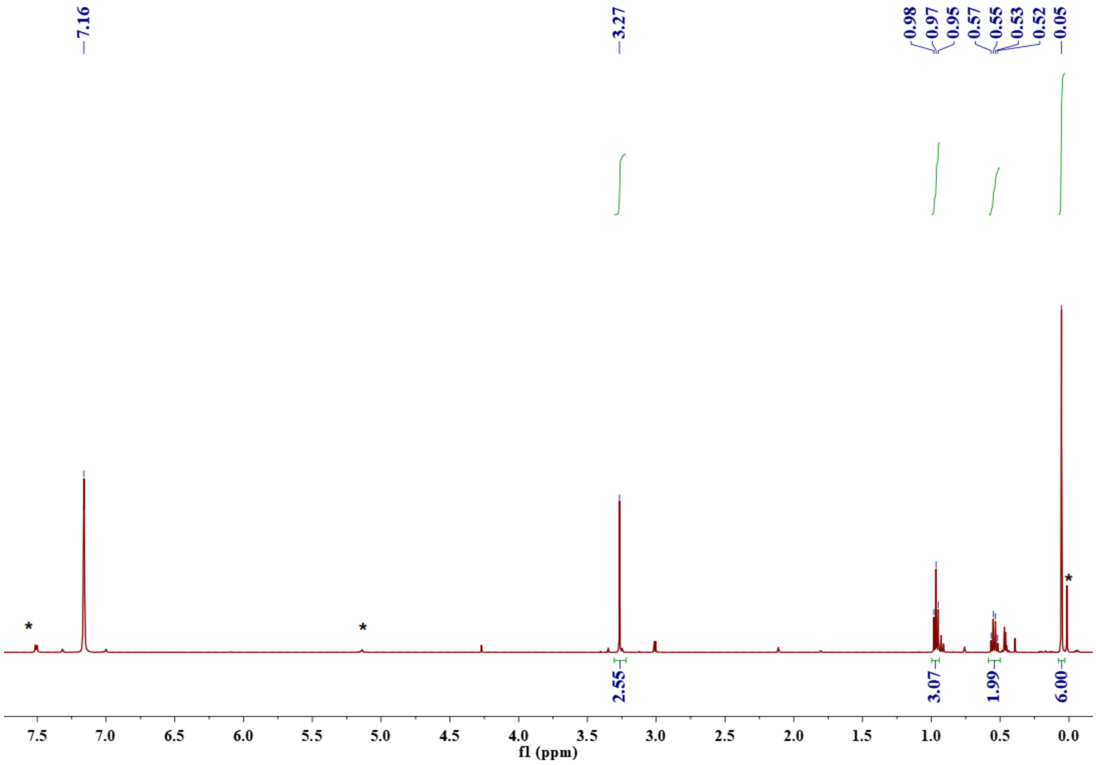

Supplementary Figure 38 ^{1}H NMR (500 MHz, C_6D_6, 25 °C) spectrum of **7b.** Signals labelled by “*” represent $HSiMePh_2$ and water.

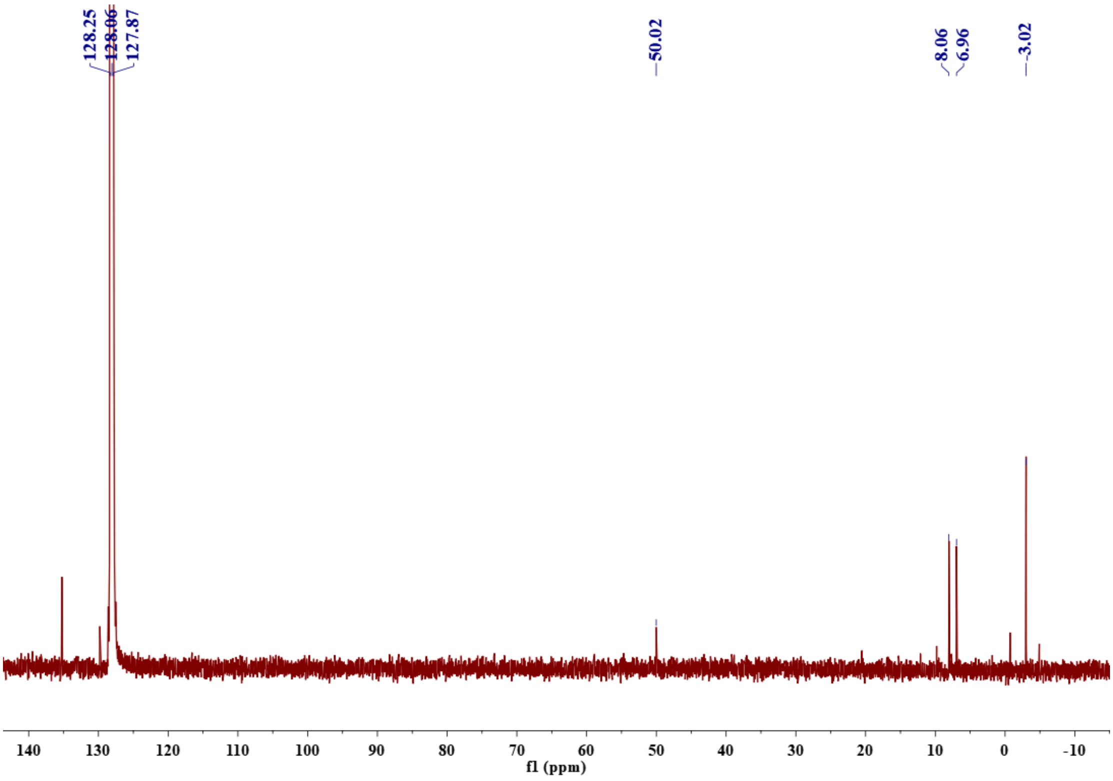

Supplementary Figure 39 ^{13}C NMR (101 MHz, C_6D_6, 25 °C) spectrum of **7b.**

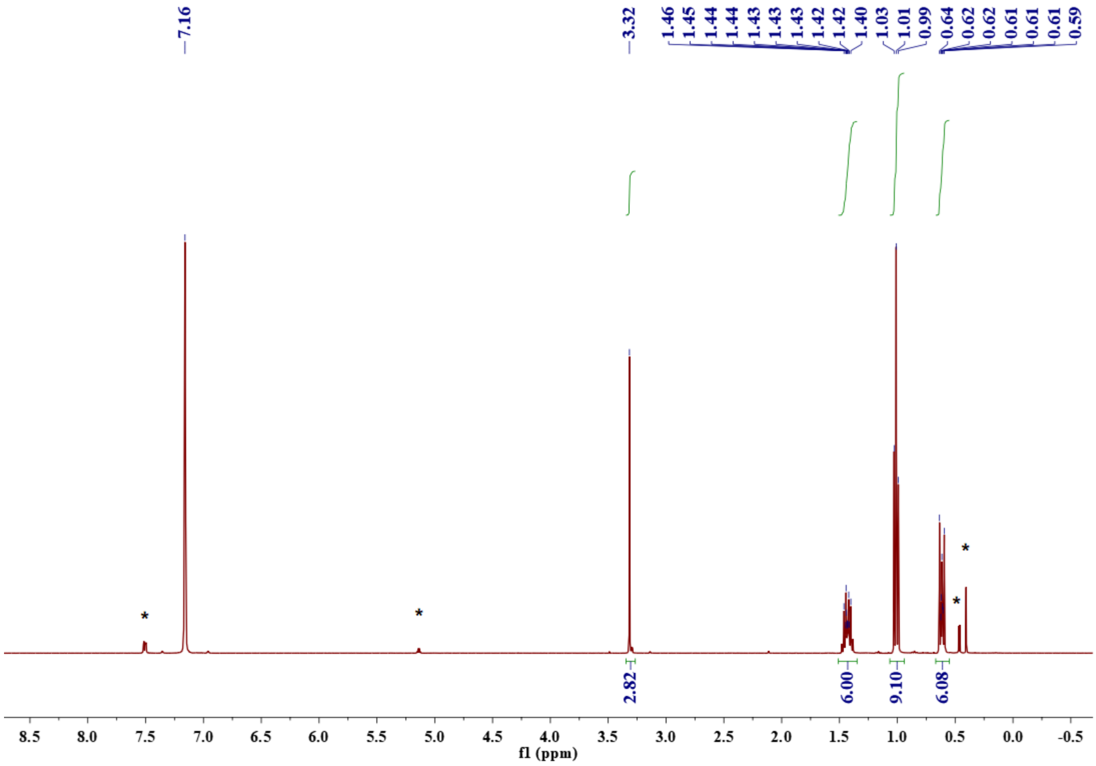

Supplementary Figure 40 ^{1}H NMR (400 MHz, C_6D_6, 25 °C) spectrum of **7c.** Signals labelled by "*" represent $HSiMePh_2$ and water.

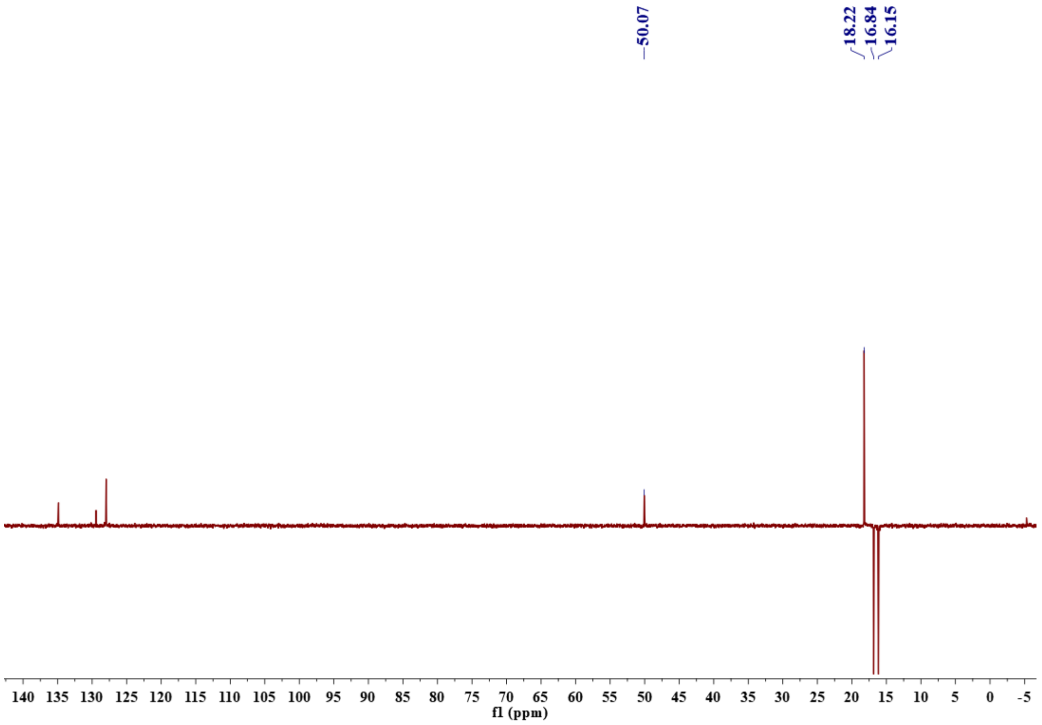

Supplementary Figure 41 ^{13}C DEPT-135 NMR (126 MHz, C_6D_6, 25 °C) spectrum of **7c.**

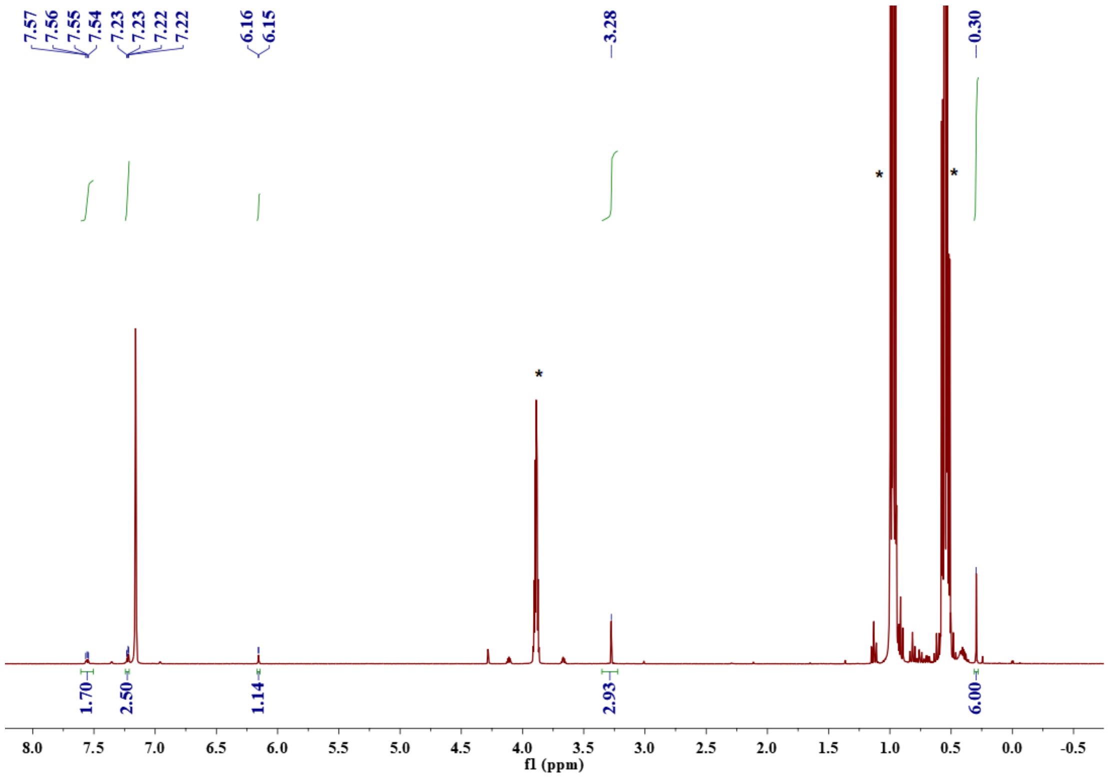

Supplementary Figure 42 ¹H NMR (400 MHz, C_6D_6, 25 °C) spectrum of **7d.** Signals labelled by "*" represent $HSiMePh_2$ and water.

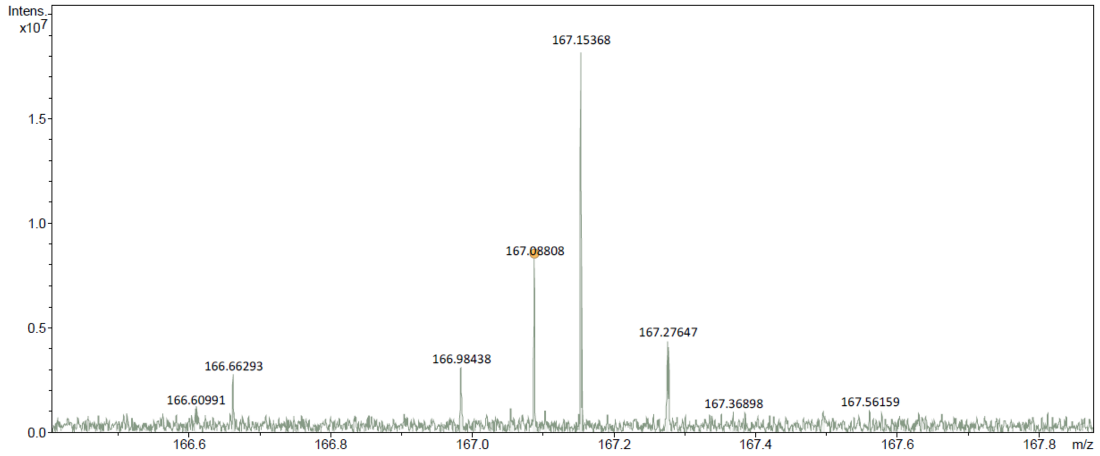

Meas. m/z	#	Ion Formula	Score	m/z	err [ppm]	Mean err [ppm]	mSigma	rdb	e⁻ Conf	N-Rule
167.088077	1	C9H15OSi	100.00	167.088668	3.5	2.0	62.9	4.0	even	ok

Supplementary Figure 43 HR-ESI-MS spectrum of **7d.**

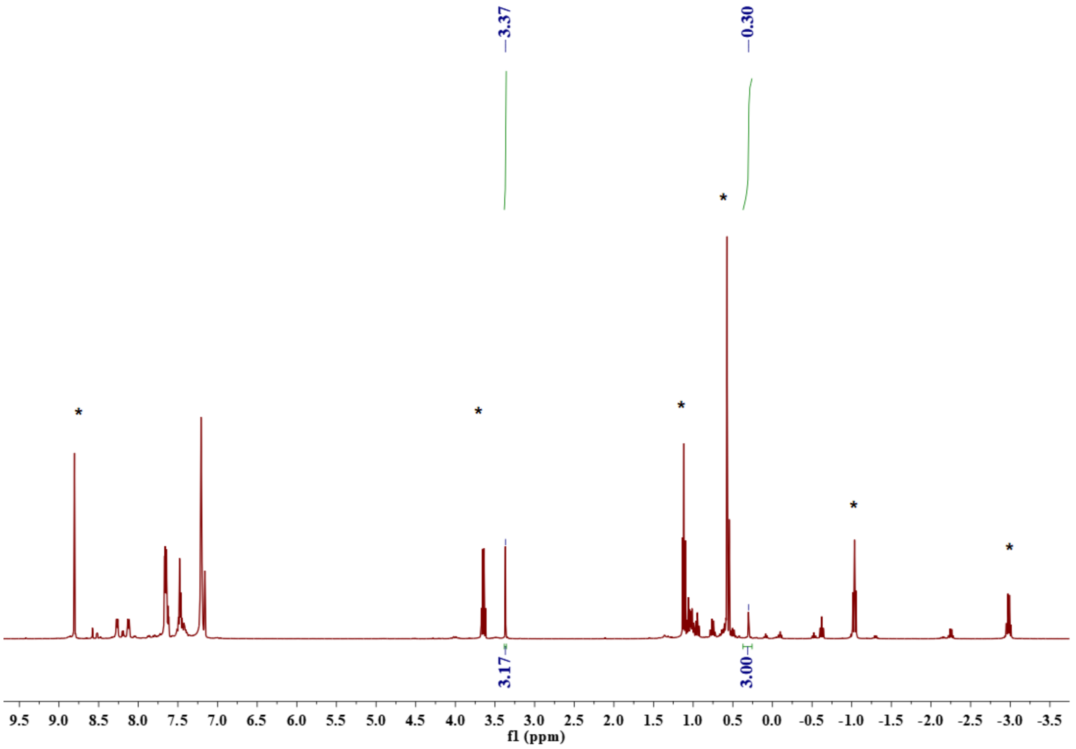

Supplementary Figure 44 ^{1}H NMR (400 MHz, C_6D_6, 25 °C) spectrum of **7e.** Signals labelled by "*" represent $HSiMePh_2$ and water.

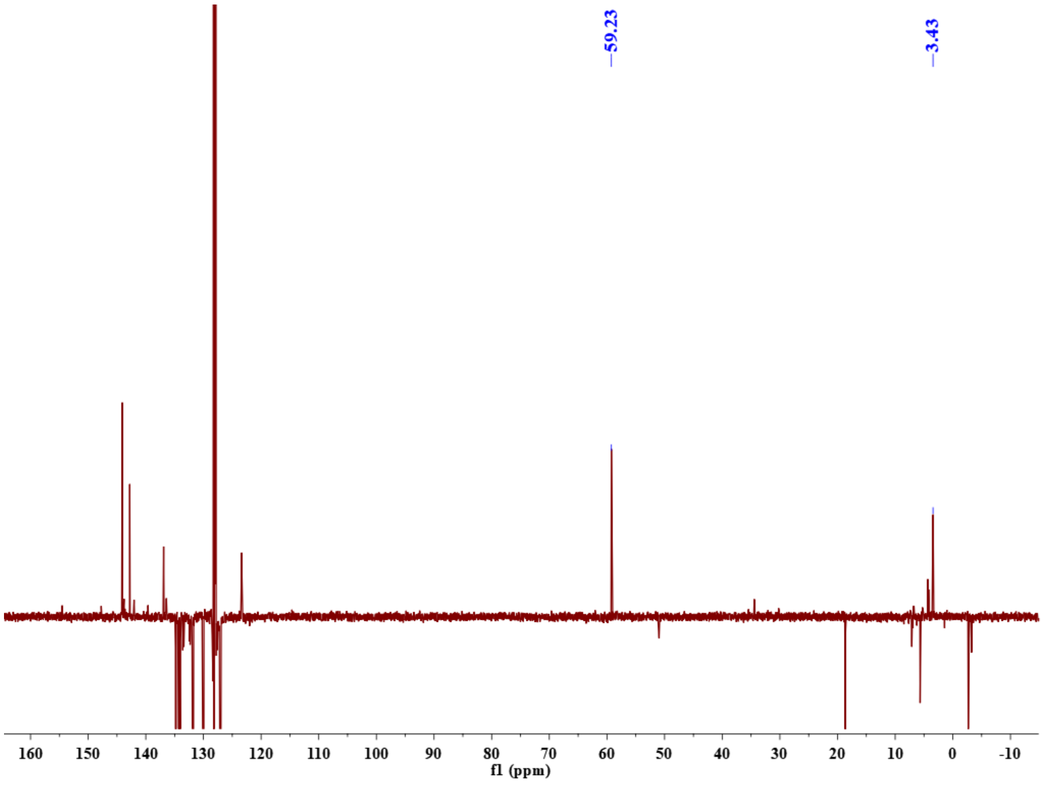

Supplementary Figure 45 ^{13}C DEPT-135 NMR (100 MHz, C_6D_6, 25 °C) spectrum of **7e.**

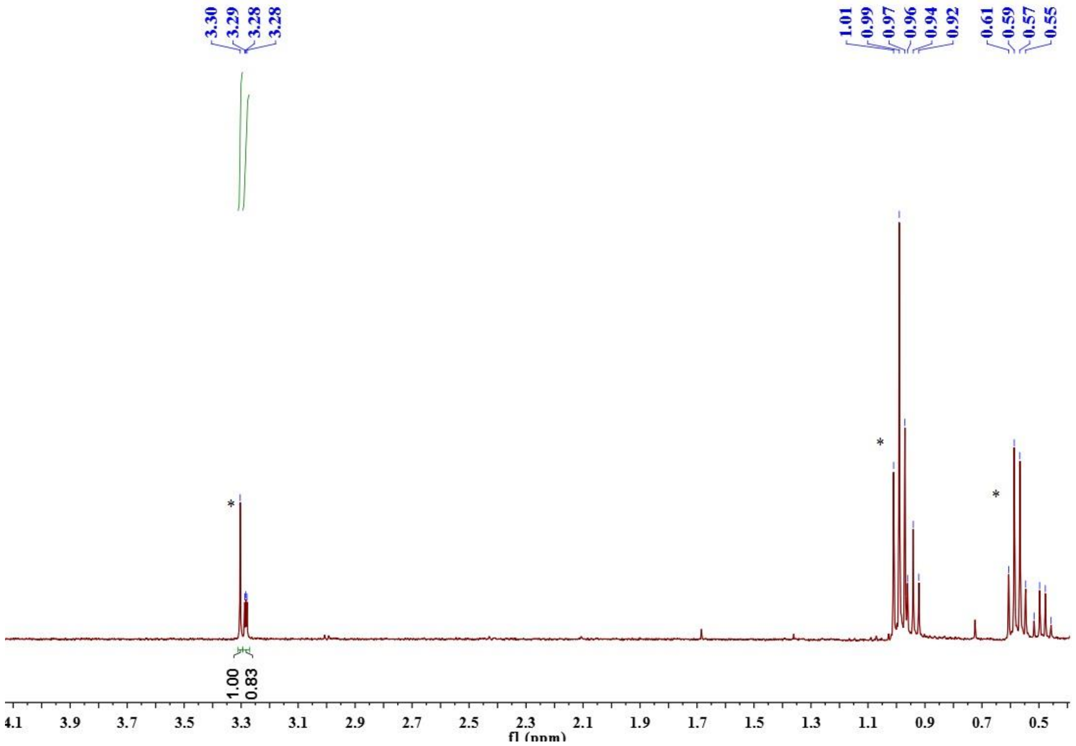

Supplementary Figure 46 ^{1}H NMR (400 MHz, C_6D_6, 25 °C) spectrum of **7f.** Signals labelled by “*” represent CH_3OSiEt_3.

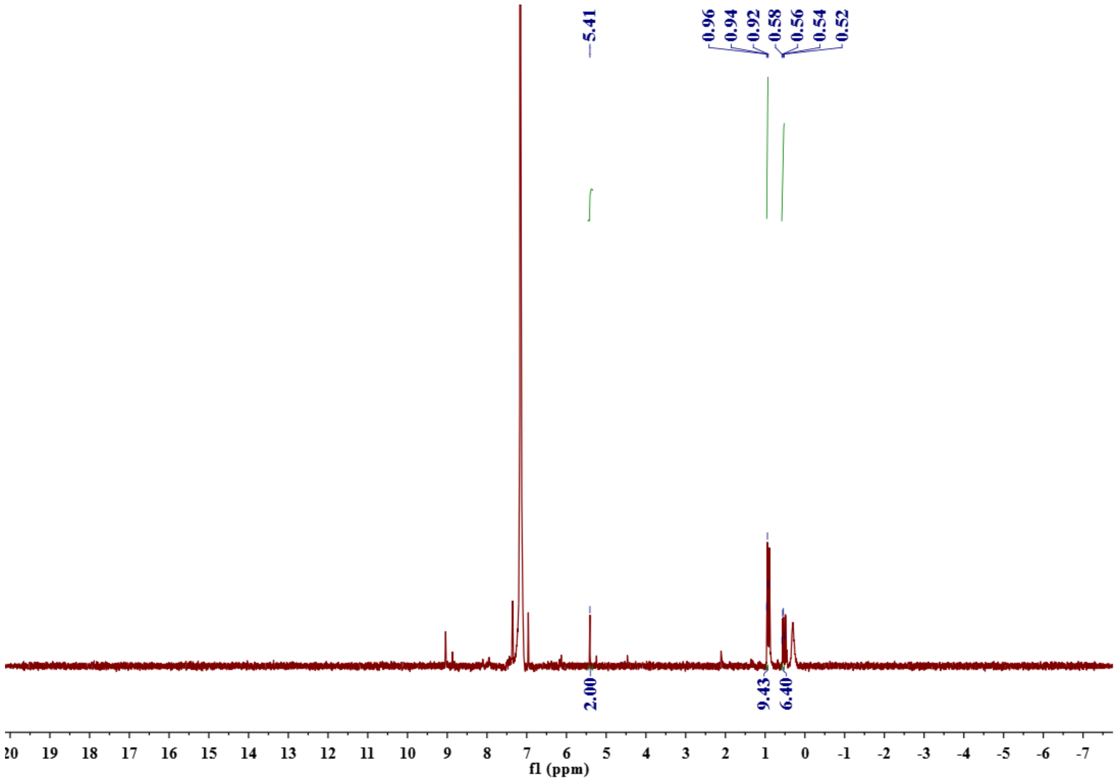

Supplementary Figure 47 ^{1}H NMR (400 MHz, C_6D_6, 25 °C) spectrum of **8.**

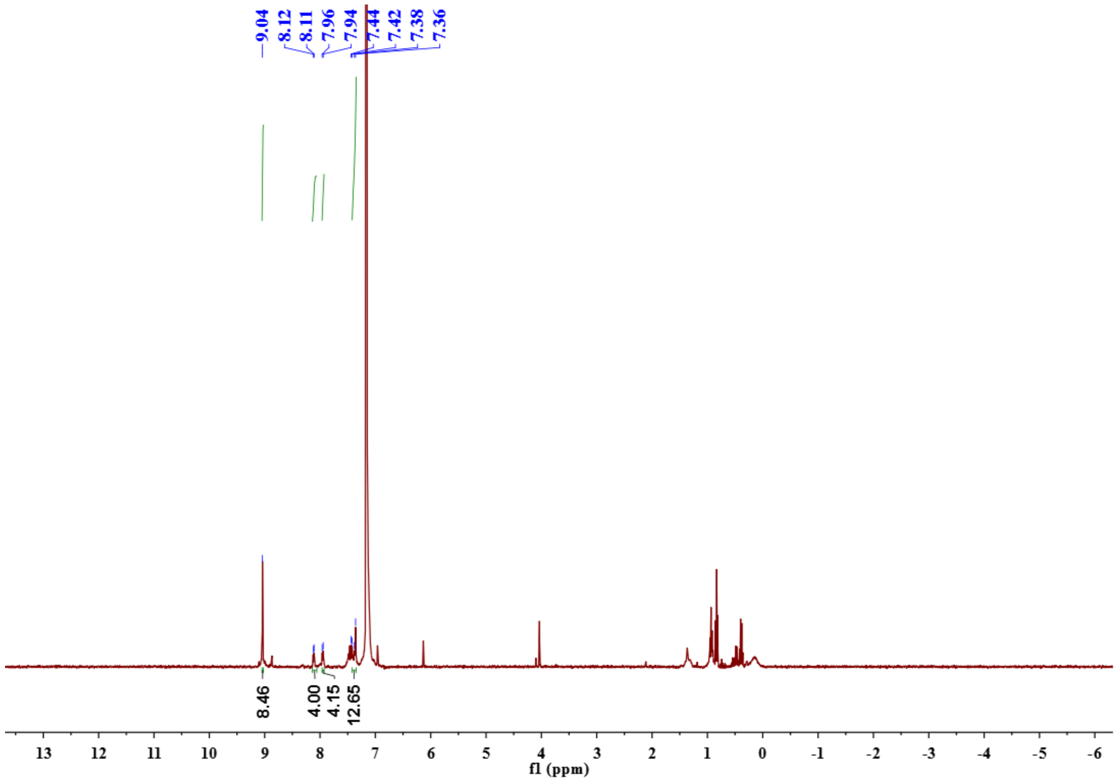

Supplementary Figure 48 ^{1}H NMR (400 MHz, C_6D_6, 25 °C) spectrum of **2.**

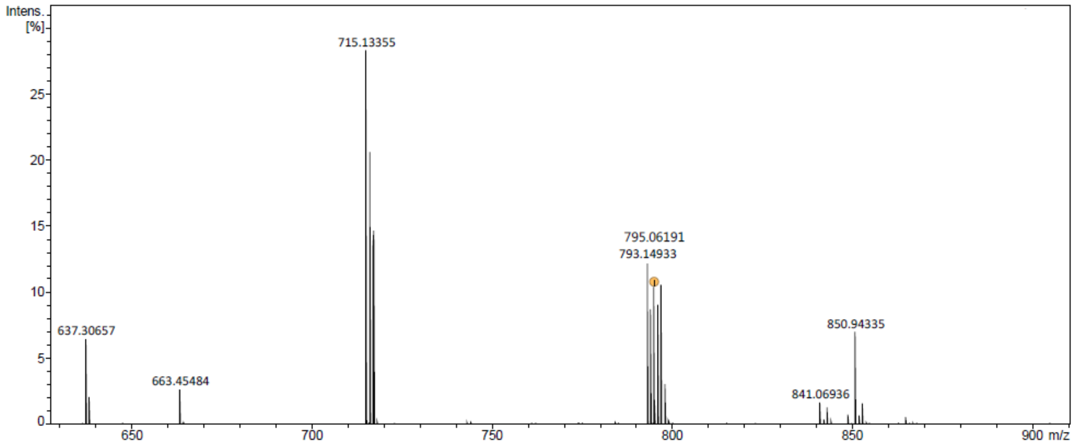

Meas. m/z	#	Ion Formula	Score	m/z	err [ppm]	Mean err [ppm]	mSigma	rdb	e⁻ Conf	N-Rule
795.061905	1	C44H29BrN4Rh	100.00	795.062509	0.8	-0.3	292.8	35.5	odd	-

Supplementary Figure 49 HR-ESI-MS spectrum of **2.**

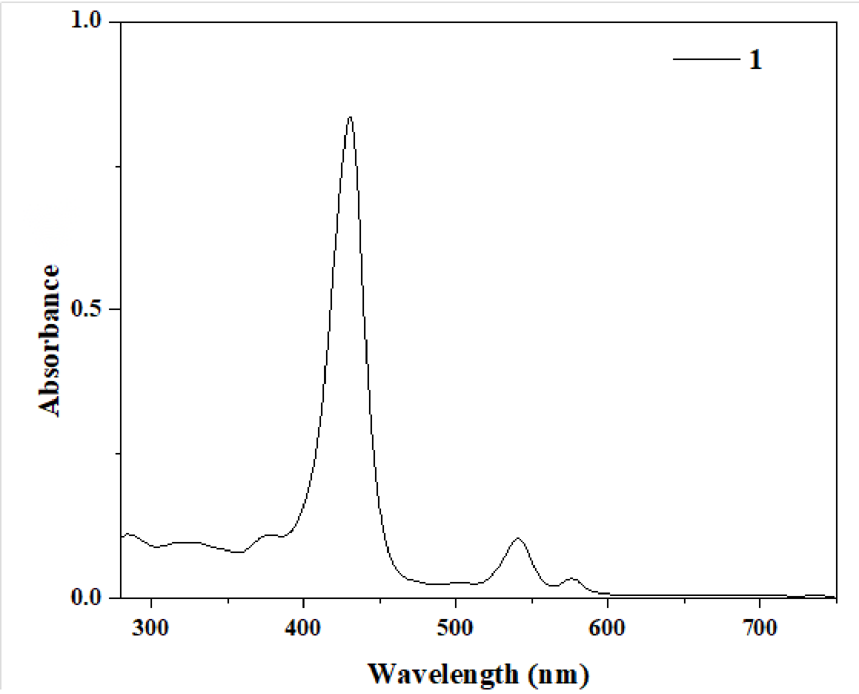

Supplementary Figure 50 UV-Vis spectrum of **1** in toluene.

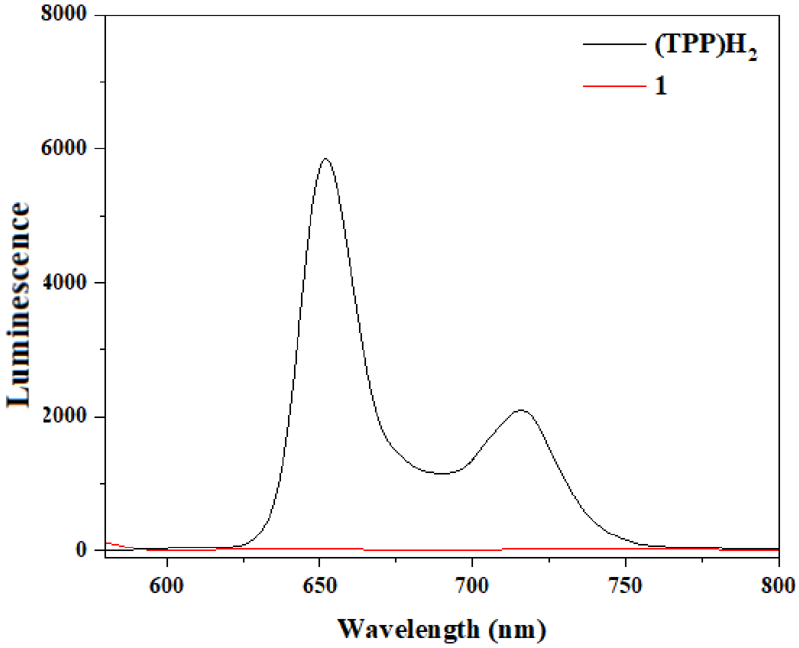

Supplementary Figure 51 Luminescence spectrum of **(TPP)RhI (1)** in toluene, **(TPP)H_2** for reference. No appreciable luminescence of **1** was found.

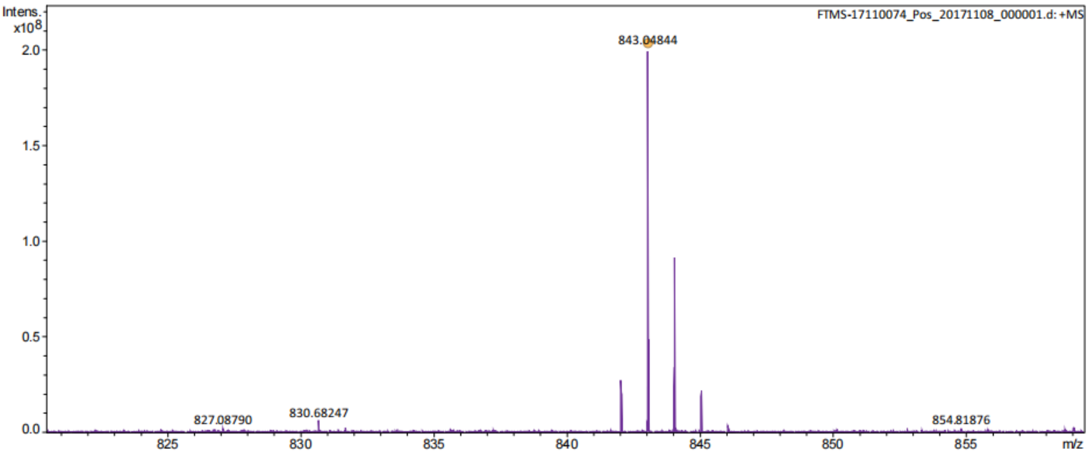

Meas. m/z	#	Ion Formula	Score	m/z	err [ppm]	Mean err [ppm]	mSigma	rdb	e⁻ Conf	N-Rule
843.048437	1	C44H29IN4Rh	100.00	843.048650	0.3	-0.1	18.5	33.0	even	ok

Supplementary Figure 52 HR-ESI-MS spectrum of **1.**

Synthesis and characterization of (TPP)RhCOCH$_2$OSiR^{1}R^{2}R^3

1 atm of ^{13}CO was pressurized into a dry deuterated toluene solution containing 2 mg of (TPP)RhCH$_2$OSiEt$_3$ in a vacuum NMR tube under dark. Subsequent variable-temperature NMR characterization revealed the presence of (TPP)Rh(^{13}CO)CH$_2$OSiEt$_3$ in ^{1}H NMR (**Figure 3**) and ^{13}C NMR (**Supplementary Figure 44**).

8 atm of CO was pressurized into a dry C_6D_6 solution containing 2 mg of (TPP)RhCH$_2$OSiR^{1}R^{2}R^3 (**6**) in a vacuum NMR tube. Then we irradiated it with 420-780 nm light provided by a 500 W high-pressure mercury lamp with a 420-780 nm filter at 6 ^{o}C over 20 hours. Subsequent NMR characterization revealed the presence of the CO insertion product (TPP)RhCOCH$_2$OSiR^{1}R^{2}R^3 (**10**) in ^{1}H NMR and ^{13}C NMR. The NMR yields are over 90 % (**Supplementary Table 5**). The solution was evaporated to dryness under reduced pressure, followed by column chromatography on silica gel with PE:DCM =3:1 to give (TPP)RhCOCH$_2$OSiR^{1}R^{2}R^3.

Supplementary Table 5 The NMR yields of the second CO insertion into Rh-C bonds.

Entry	Substrate	Product	Yield
1	(TPP)RhCH$_2$OSiEt3 **6a**	(TPP)RhCOCH$_2$OSiEt$_3$ **10a**	92%
2	(TPP)RhCH$_2$OSiMe$_2$Et **6b**	(TPP)RhCOCH$_2$OSiMe$_2$Et **10b**	91%
3	(TPP)RhCH$_2$OSi^{n}Pr$_3$ **6c**	(TPP)RhCOCH$_2$OSi^{n}Pr$_3$ **10c**	91%
4	(TPP)RhCH$_2$OSiMe$_2$Ph **6d**	(TPP)RhCOCH$_2$OSiMe$_2$Ph **10d**	93%
5	(TPP)RhCH$_2$OSiMePh$_2$ **6e**	(TPP)RhCOCH$_2$OSiMePh$_2$ **10e**	92%

(TPP)RhCOCH$_2$OSiEt$_3$ (10a): ^{1}H NMR (400 MHz, C_6D_6) δ (ppm): 8.90 (s, 8 H, pyrrole), 8.21 (m, 4 H, *o*-phenyl), 8.12 (m, 4 H, *o'*-phenyl), 7.50 (m, 12 H, *m*-phenyl, *p*-phenyl), 0.06 (t, 9 H, $^3J_{H-H}$ = 8.0 Hz, Si-CH$_2$C*H$_3$*), -0.71 (q, 6 H, $^3J_{H-H}$ = 8.0 Hz, Si-C*H$_2$*CH$_3$), -1.52 (s, 2 H, Si-O-C*H$_2$*-CO-Rh); ^{13}C NMR (126 MHz, C_6D_6) δ (ppm): 198.00 (d, $^1J_{Rh-C}$ = 31.7 Hz, Rh-*C*OCH$_2$-), 143.44 (porphyrin carbon), 142.69 (porphyrin carbon), 134.65 (porphyrin carbon), 134.16 (porphyrin carbon), 132.02 (porphyrin carbon), 127.02 (porphyrin carbon), 123.09 (porphyrin carbon), 62.79 (d, $^2J_{Rh-C}$ = 4.7 Hz, SiO*C*H$_2$-C(O)Rh), 6.13 (s, Si-CH$_2$*C*H$_3$), 3.50 (s, Si-*C*H$_2$CH$_3$). UV-Vis: λ_{abs} (nm) (toluene) 415, 521, 574. HR-ESI-MS m/z calcd for $C_{52}H_{46}N_4O_2RhSi$, [M+H]$^+$ 889.24396; found 860.24590; FTIR $\nu_{CO} \approx$ 1731 cm^{-1}. Single crystals suitable for XRD studies were obtained by slow diffusion from a concentrated THF solution to deionized water.

(TPP)RhCOCH$_2$OSiMe$_2$Et (10b): ^{1}H NMR (400 MHz, C_6D_6) δ (ppm): 8.90 (s, 8 H, pyrrole), 8.21 (m, 4 H, *o*-phenyl), 8.09 (m, 4 H, *o'*-phenyl), 7.46 (m, 12 H, *m*-phenyl, *p*-phenyl), 0.07 (t, 3 H, $^3J_{H-H}$ = 8.0 Hz, Si-CH$_2$C*H$_3$*), -0.71 (q, 2 H, $^3J_{H-H}$ = 8.0 Hz, Si-C*H$_2$*CH$_3$), -1.27 (s, 6 H, Si-C*H$_3$*), -1.54 (s, 2 H, Si-O-C*H$_2$*-CO-Rh); ^{13}C NMR (126 MHz, C_6D_6) δ (ppm): 198.42 (d, $^1J_{Rh-C}$ = 31.2 Hz, Rh-*C*OCH$_2$-), 143.44 (porphyrin carbon), 142.68 (porphyrin carbon), 134.66 (porphyrin carbon), 134.10 (porphyrin carbon), 132.02 (porphyrin carbon), 127.02 (porphyrin carbon), 123.08 (porphyrin carbon), 62.70 (d, $^2J_{Rh-C}$ = 4.9 Hz, SiO*C*H$_2$-C(O)Rh), 7.17 (Si-CH$_2$*C*H$_3$), 6.09 (s, Si-*C*H$_2$CH$_3$), -4.02 (s, Si-*C*H$_3$); HR-ESI-MS m/z calcd for $C_{50}H_{42}N_4O_2RhSi$, [M+H]$^+$ 861.212659; found 861.21356; FTIR $\nu_{CO} \approx$1731 cm^{-1}.

(TPP)RhCOCH$_2$OSi^{n}Pr$_3$ (10c): ^{1}H NMR (500 MHz, C_6D_6) δ (ppm): 8.92 (s, 8 H, pyrrole), 8.21 (m, 4 H, *o*-phenyl), 8.15 (m, 4 H, *o'*-phenyl), 7.49 (m, 12 H, *m*-phenyl, *p*-phenyl), 0.52 (t, 9 H, ^3J = 8.0 Hz, Si-CH$_2$CH$_2$C*H$_3$*), 0.42 (m, 6 H, Si-CH$_2$C*H$_2$*CH$_3$), -0.64 (m, 6 H, Si-C*H$_2$*CH$_2$CH$_3$), -1.53 (s, 2 H, Si-O-C*H$_2$*-CO-Rh); ^{13}C NMR (126 MHz, C_6D_6) δ (ppm): 197.51 (d, $^1J_{Rh-C}$ = 30.8 Hz,

Rh-$\underline{C}OCH_2$-), 143.45 (porphyrin carbon), 142.69 (porphyrin carbon), 134.73 (porphyrin carbon), 134.18 (porphyrin carbon), 132.03 (porphyrin carbon), 127.01 (porphyrin carbon), 123.11 (porphyrin carbon), 62.58 (d, ^2J = 4.7 Hz, SiO$\underline{C}H_2$-C(O)Rh), 18.19 (Si-$CH_2CH_2\underline{C}H_3$), 16.23 (s, Si-$CH_2\underline{C}H_2CH_3$), 15.37 (s, Si-$\underline{C}H_2CH_2CH_3$); HR-ESI-MS m/z calcd for $C_{55}H_{51}N_4O_2RhSi$, $[M]^+$ 930.28306; found 930.28414; FTIR $\nu_{CO} \approx 1730$ cm^{-1}.Single crystals suitable for XRD studies were obtained by slow diffusion from a concentrated THF solution to deionized water.

(TPP)RhCOCH$_2$OSiMe$_2$Ph (10d): ^{1}H NMR (500 MHz, C_6D_6) δ (ppm): 8.87 (s, 8 H, pyrrole), 8.21 (m, 4 H, *o*-phenyl), 8.97 (m, 4 H, *o'*-phenyl), 7.46 (m, 12 H, *m*-phenyl, *p*-phenyl), 7.02 (m, 1 H, Si-phenyl), 6.90 (m, 2 H, Si-phenyl), 6.52 (s, 2 H, Si-phenyl), -1.10 (s, 6 H, Si-O-C$\underline{H_2}$-CO-Rh), -1.10 (s, 6 H, Si-C$\underline{H_3}$), -1.55 (s, 2 H, Si-O-C$\underline{H_2}$-CO-Rh); HR-ESI-MS m/z calcd for $C_{54}H_{42}N_4O_2RhSi$, $[M+H]^+$ 909.21266; found 909.21354; FTIR $\nu_{CO} \approx 1726$ cm^{-1}.

(TPP)RhCOCH$_2$OSiMePh$_2$ (10e): ^{1}H NMR (500 MHz, C_6D_6) δ (ppm): 8.86 (s, 8 H, pyrrole), 8.21 (m, 4 H, *o*-phenyl), 7.89 (m, 4 H, *o'*-phenyl), 7.51 (m, 8 H, *m*-phenyl), 7.43 (m, 4 H, *p*-phenyl), 6.96 (m, 2 H, Si-phenyl), 6.83 (m, 4 H, Si-phenyl), 6.58 (m, 4 H, Si-phenyl), -1.12 (s, 3 H, Si-C$\underline{H_3}$), -1.49 (s, 2 H, Si-O-C$\underline{H_2}$-CO-Rh); ^{13}C NMR (126 MHz, C_6D_6) δ (ppm): 195.22 (d, $^1J_{Rh\text{-}C}$ = 31.8 Hz, Rh-$\underline{C}OCH_2$-), 141.41 (s, porphyrin carbon), 140.64 (s, porphyrin carbon), 132.92 (s, porphyrin carbon), 132.81 (s, porphyrin carbon), 132.06 (s, Si-phenyl), 131.95 (s, porphyrin carbon), 130.01 (s, Si-phenyl), 127.67 (s, Si-phenyl), 125.76 (s, Si-phenyl),124.99 (porphyrin carbon), 121.09 (s, porphyrin carbon), 59.93 (d, $^2J_{Rh\text{-}C}$ = 4.8 Hz, Si-O-$\underline{C}H_2$-CO-Rh), -6.79 (s, Si-$\underline{C}H_3$); HR-ESI-MS m/z calcd for $C_{59}H_{43}N_4O_2RhSi$, $[M]^+$ 970.22103; found 971.22994; FTIR $\nu_{CO} \approx 1726$ cm^{-1}.

(TPP)Rh^{13}COCH$_2$OSiEt$_3$ (10f): ^{1}H NMR (400 MHz, C_6D_6) δ (ppm): 8.90 (s, 8 H, pyrrole), 8.21 (m, 4 H, *o*-phenyl), 8.12 (m, 4 H, *o'*-phenyl), 7.45 (m, 12 H, *m*-phenyl, *p*-phenyl), 0.06 (t, 9 H, $^3J_{H\text{-}H}$ = 8.0 Hz, Si-$CH_2C\underline{H_3}$), -0.72 (q, 6 H, $^3J_{H\text{-}H}$ = 8.0 Hz, Si-C$\underline{H_2}$CH$_3$), -1.52 (d, 2 H, $^2J_{13C\text{-}H}$ = 2.1 Hz, Si-O-C$\underline{H_2}$-^{13}CO-Rh); ^{13}C NMR (126 MHz, C_6D_6) δ (ppm): 198.00 (d, $^1J_{Rh\text{-}C}$ = 31.2 Hz, Rh-$\underline{C}OCH_2$-), 143.44 (porphyrin carbon), 142.70 (porphyrin carbon), 134.65 (porphyrin carbon), 134.16 (porphyrin carbon), 132.01 (porphyrin carbon), 127.02 (porphyrin carbon), 123.09 (porphyrin carbon), 62.78 (dd, $^1J_{13C\text{-}C}$ = 33.2 Hz, $^2J_{Rh\text{-}C}$ = 4.7 Hz, SiO$\underline{C}H_2$-^{13}C(O)Rh), 6.13

(Si-$CH_2\underline{C}H_3$), 3.50 (s, Si-$\underline{C}H_2CH_3$); HR-ESI-MS m/z calcd for $C_{51}H_{46}N_4O_2RhSi^{13}C$, $[M+H]^+$ 890.24731, found 890.24846; FTIR $\nu_{13CO} \approx 1705\ cm^{-1}$.

(TPP)Rh^{13}CO^{13}CH$_2$OSiEt$_3$ (10g): ^{1}H NMR (500 MHz, C_6D_6) δ (ppm): 8.90 (s, 8 H, pyrrole), 8.21 (m, 4 H, *o*-phenyl), 8.12 (m, 4 H, *o'*-phenyl), 7.50 (m, 12 H, *m*-phenyl, *p*-phenyl), 0.06 (t, 9 H, $^3J_{H\text{-}H}$ = 8.0 Hz, Si-$CH_2C\underline{H_3}$), -0.71 (q, 6 H, $^3J_{H\text{-}H}$ = 8.0 Hz, Si-$C\underline{H_2}CH_3$), -1.52 (dd, 2 H, $^1J_{13C\text{-}H}$ = 149.2 Hz, $^2J_{13C\text{-}H}$ = 2.1 Hz, Si-O-$^{13}C\underline{H_2}$-^{13}CO-Rh); ^{13}C NMR (126 MHz, C_6D_6) δ (ppm): 198.00 (dd, $^1J_{13C\text{-}C}$ = 32.9, $^1J_{Rh\text{-}C}$ = 31.7 Hz, Rh-$^{13}\underline{C}$O^{13}CH$_2$-), 62.79 (dd, $^1J_{13C\text{-}C}$ = 33.0 Hz, $^2J_{Rh\text{-}C}$ = 4.9 Hz, SiO$^{13}\underline{C}$H$_2$-^{13}C(O)Rh), other peaks are not obvious because of low concentration; HR-ESI-MS m/z calcd for $C_{50}H_{46}N_4O_2RhSi^{13}C_2$, $[M+H]^+$ 891.25067; found 891.24991; FTIR $\nu_{13CO} \approx 1658\ cm^{-1}$.

Summary for spectroscopy

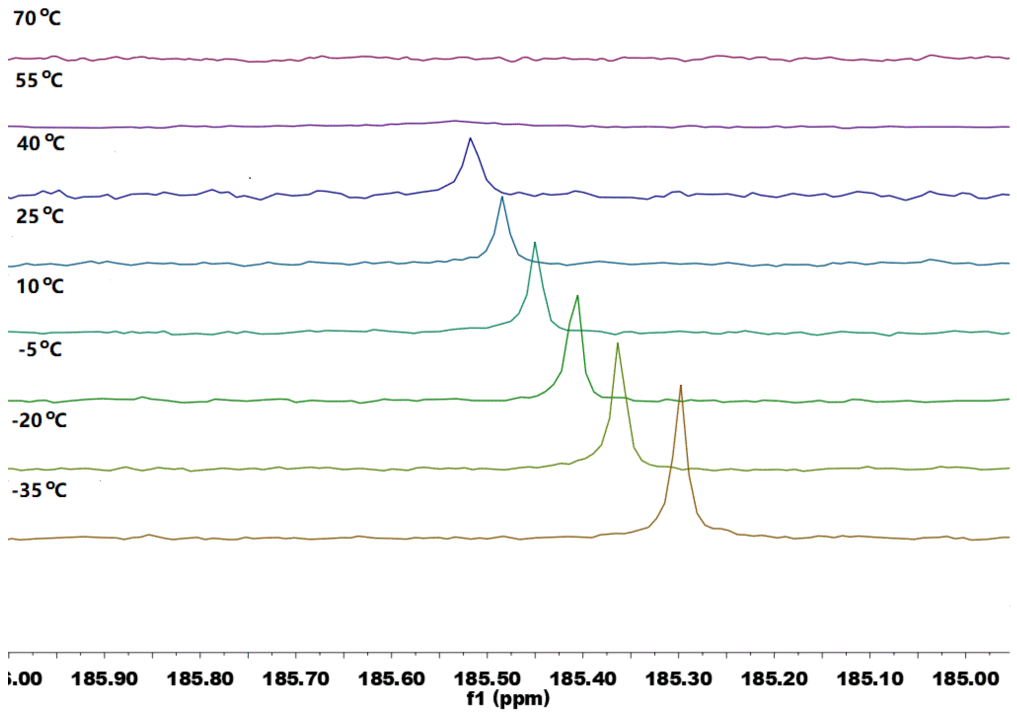

Supplementary Figure 53 ^{13}C NMR spectra of **(TPP)Rh(^{13}CO)CH$_2$OSiEt$_3$ (6a-^{13}CO)** in deuterated toluene under different temperatures.

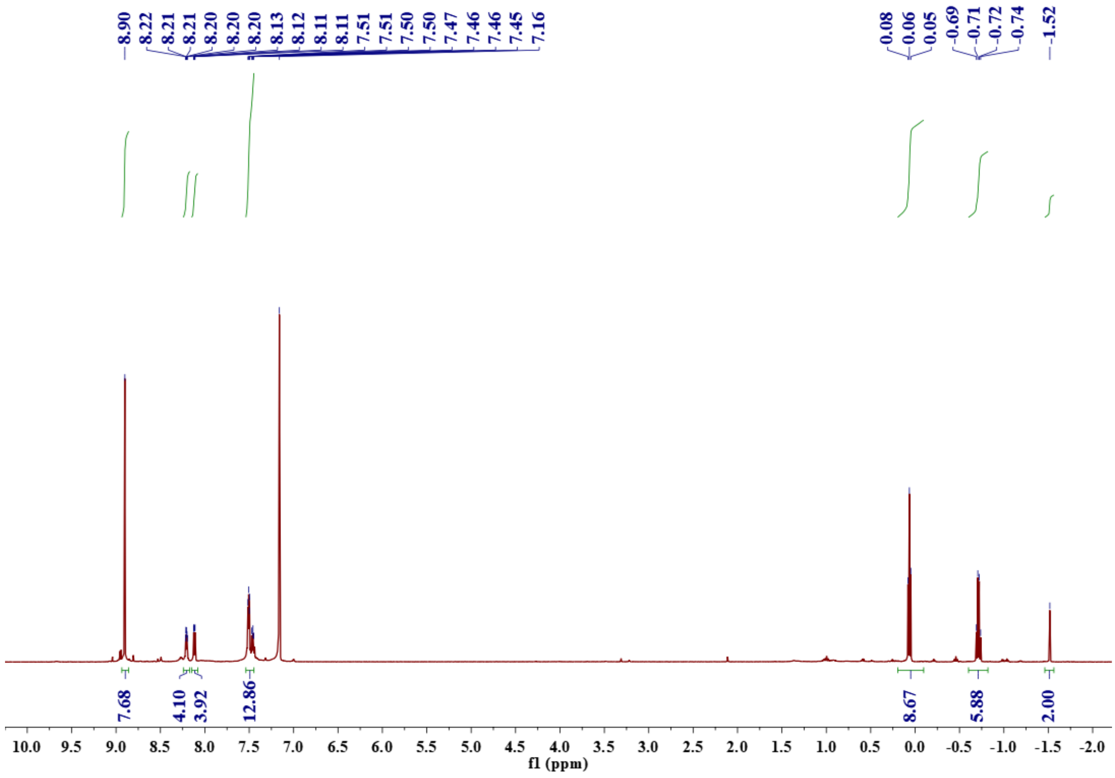

Supplementary Figure 54 ^{1}H NMR (400 MHz, C_6D_6, 25 °C) spectrum of **10a.**

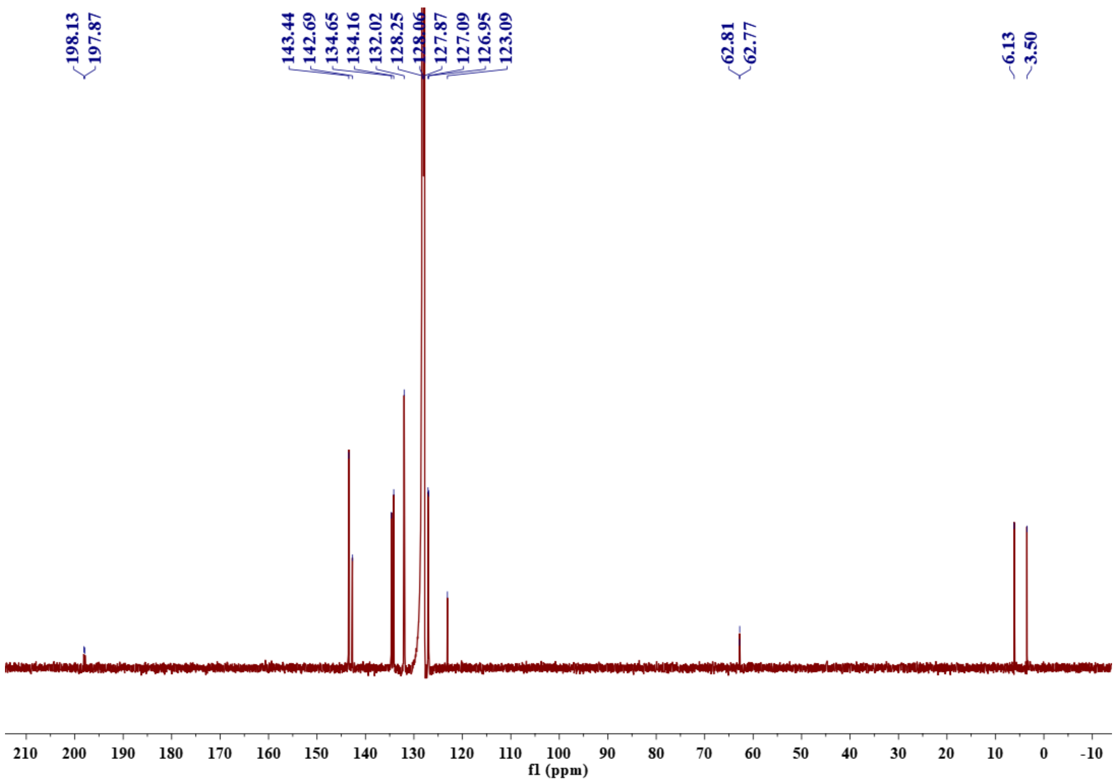

Supplementary Figure 55 ^{13}C APT NMR (126 MHz, C_6D_6, 25 °C) spectrum of **10a.**

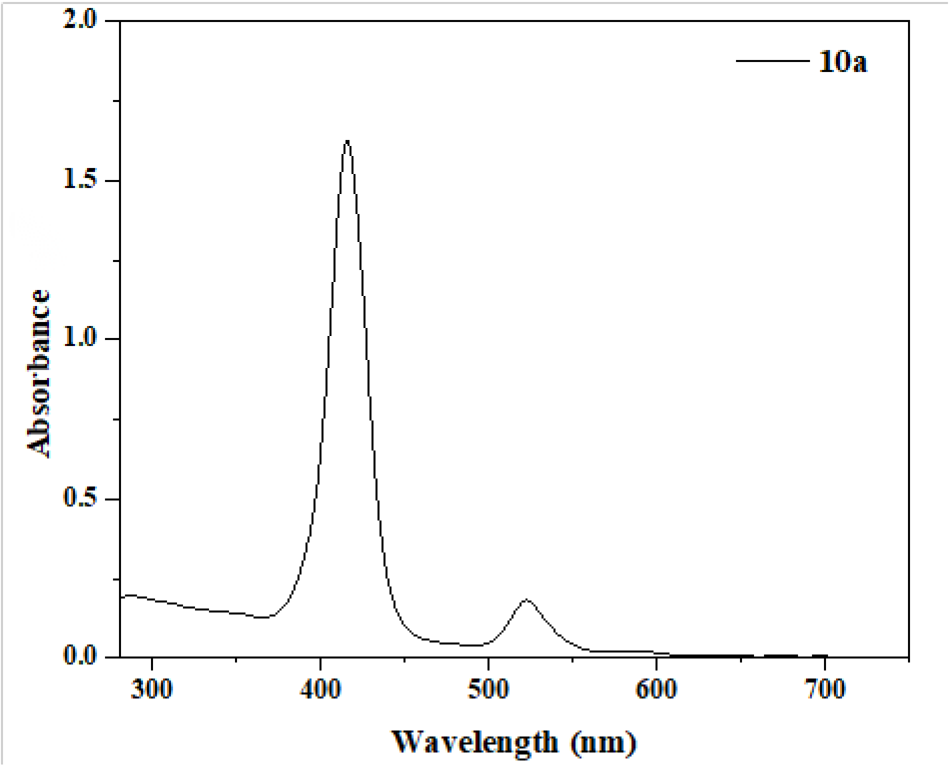

Supplementary Figure 56 UV-Vis spectrum of **10a** in toluene.

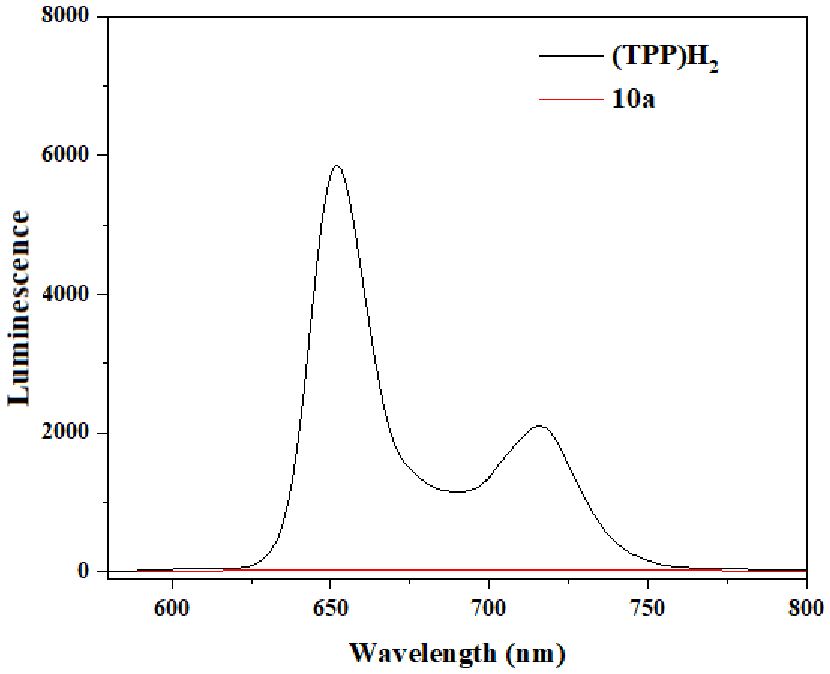

Supplementary Figure 57 Luminescence spectrum of **10a** in toluene, $(TPP)H_2$ for reference. No appreciable luminescence of **10a** was found.

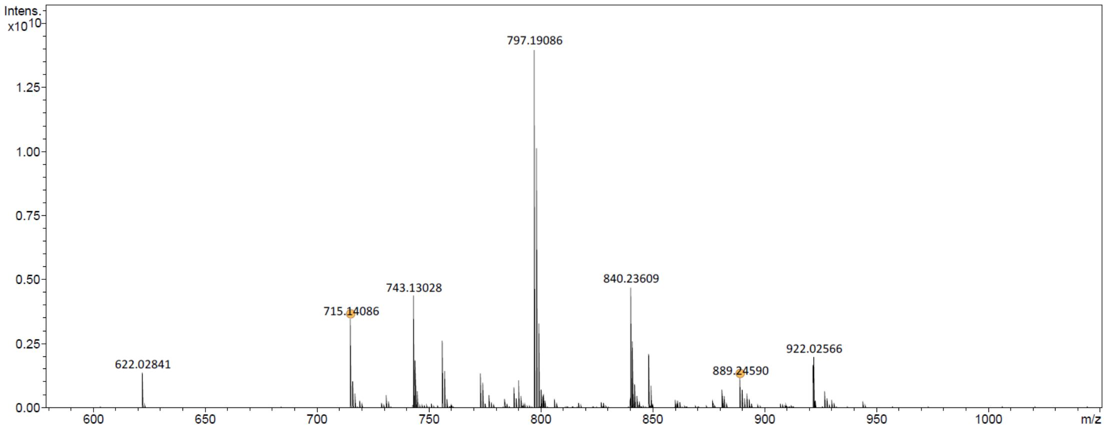

Meas. m/z	#	Ion Formula	Score	m/z	err [ppm]	Mean err [ppm]	mSigma	rdb	e⁻ Conf	N-Rule
715.140864	1	C44H28N4Rh	100.00	715.136352	-6.3	-1.9	113.0	33.5	even	ok
889.245898	1	C52H46N4O2RhSi	100.00	889.243959	-2.2	-6.8	234.2	33.5	even	ok

Supplementary Figure 58 HR-ESI-MS spectrum of **10a.**

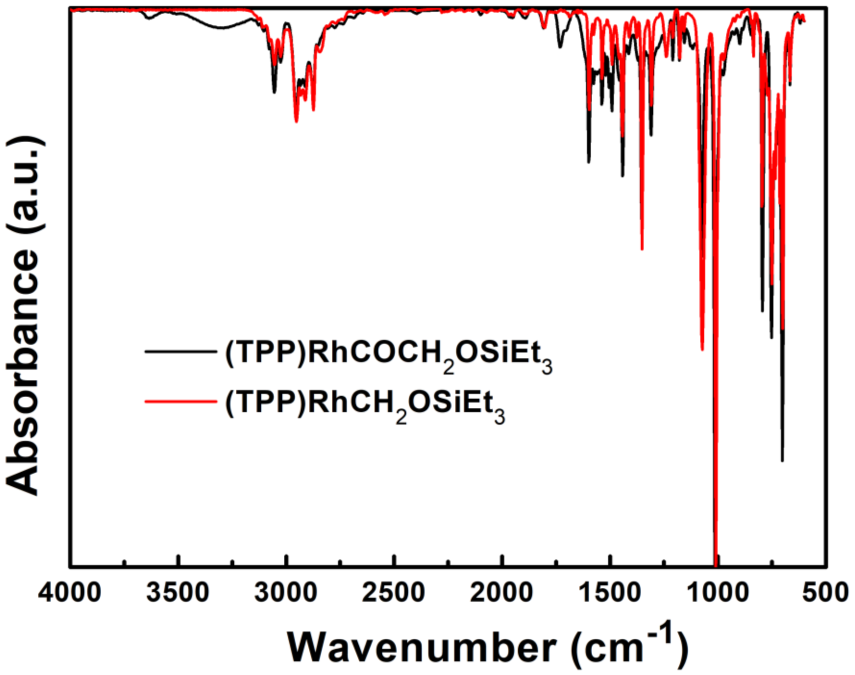

Supplementary Figure 59 FTIR spectrum of **10a**, compared against the spectrum of **6a.**

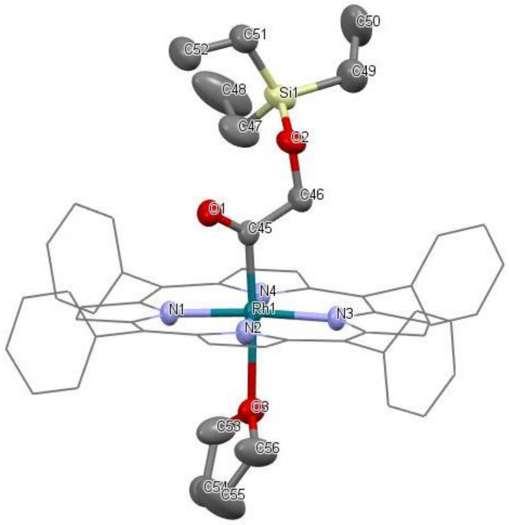

Supplementary Figure 60 X-ray crystal structure presentation of the molecular structure for **10a·THF.** Thermal ellipsoids set at 50% probability; hydrogen atoms and some labels are omitted for clarity. Gray: carbon, yellow: silicon, red: oxygen, light blue: nitrogen, dark blue-green: rhodium.

Supplementary Table 6 Crystal data and structure refinement for **10a**·**THF**.

Empirical formula	C116 H114 N8 O7 Rh2 Si2	
Formula weight	1994.15	
Temperature	173.1500 K	
Wavelength	0.71073 Å	
Crystal system	Monoclinic	
Space group	C 1 2/c 1	
Unit cell dimensions	a = 24.496(2) Å	α = 90°.
	b = 10.6767(9) Å	β = 103.700(13)°.
	c = 39.085(4) Å	γ = 90°.
Volume	9931.5(17) Å^3	
Z	4	

Density (calculated)	1.334 Mg/m^3
Absorption coefficient	0.418 mm^{-1}
F(000)	4160
Crystal size	0.243 x 0.218 x 0.079 mm^3
Theta range for data collection	1.711 to 27.489°.
Index ranges	-31<=h<=31, -13<=k<=13, -50<=l<=50
Reflections collected	65994
Independent reflections	11372 [R(int) = 0.0367]
Completeness to theta = 26.000°	99.9 %
Absorption correction	Semi-empirical from equivalents
Max. and min. transmission	1.00000 and 0.82517
Refinement method	Full-matrix least-squares on F^2
Data / restraints / parameters	11372 / 0 / 612
Goodness-of-fit on F^2	1.088
Final R indices [I>2sigma(I)]	R1 = 0.0485, wR2 = 0.1201
R indices (all data)	R1 = 0.0515, wR2 = 0.1224
Extinction coefficient	n/a
Largest diff. peak and hole	0.634 and -0.471 e.Å^{-3}

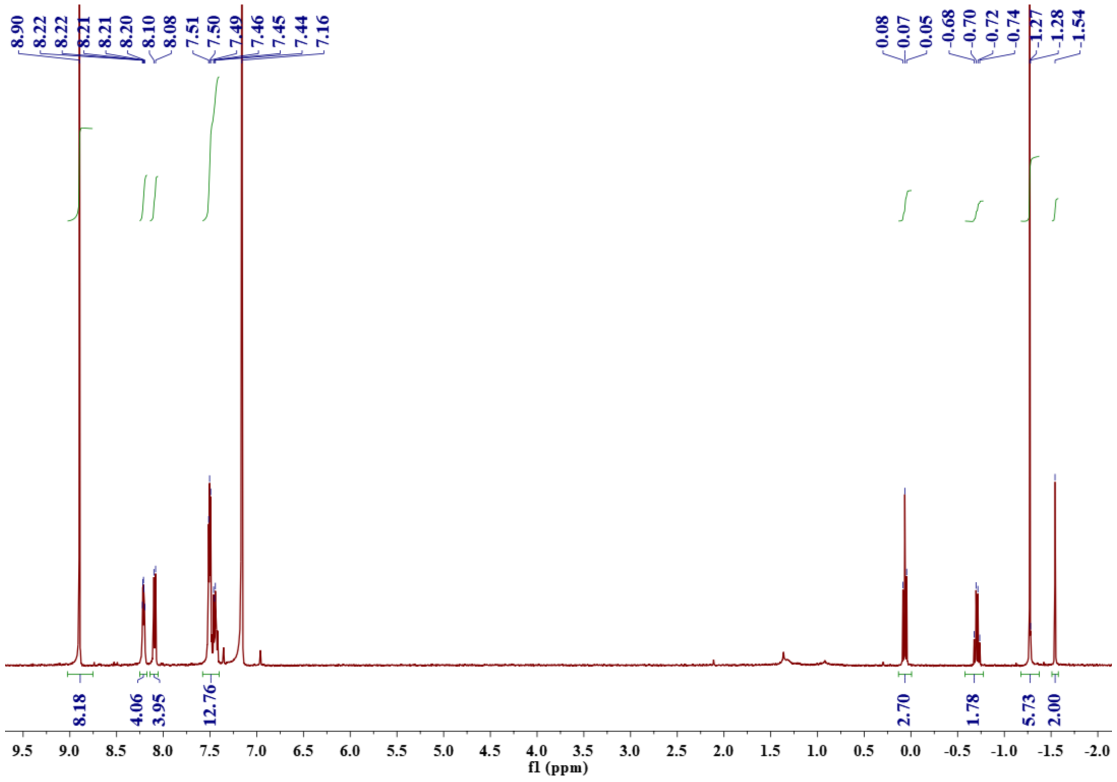

Supplementary Figure 61 ^{1}H NMR (400 MHz, C_6D_6, 25 °C) spectrum of **10b.**

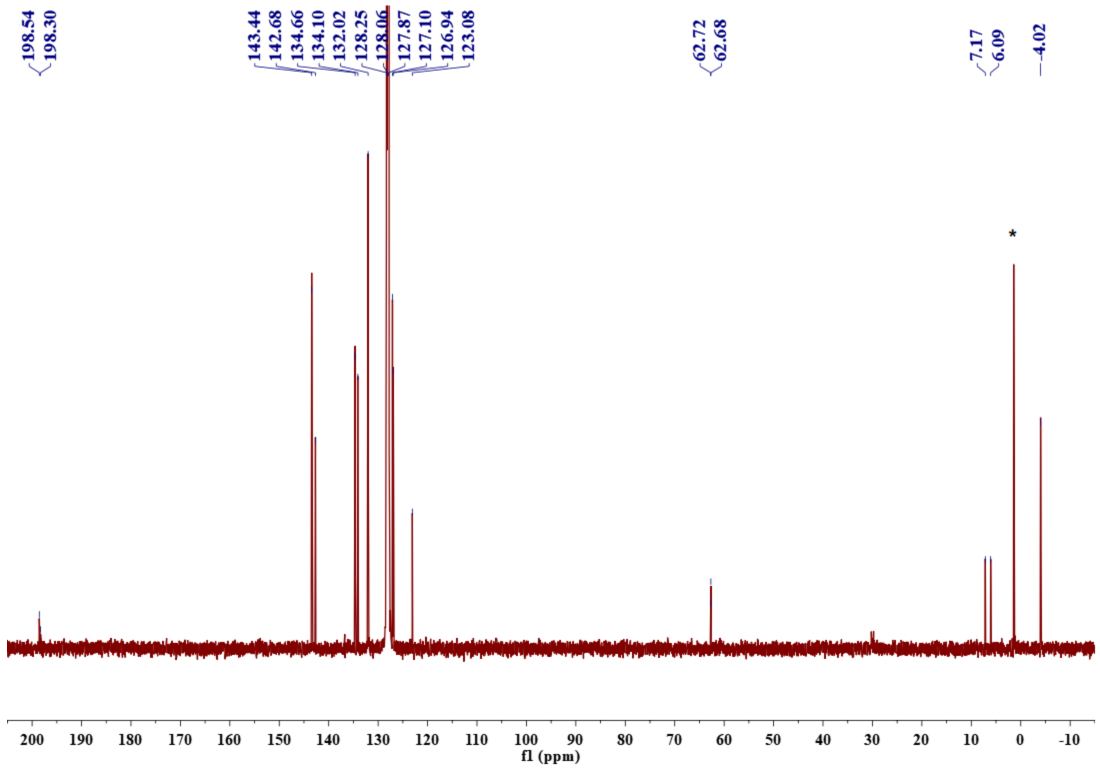

Supplementary Figure 62 ^{13}C NMR (126 MHz, C_6D_6, 25 °C) spectrum of **10b.** Signals labelled by "*" represent silicone grease.

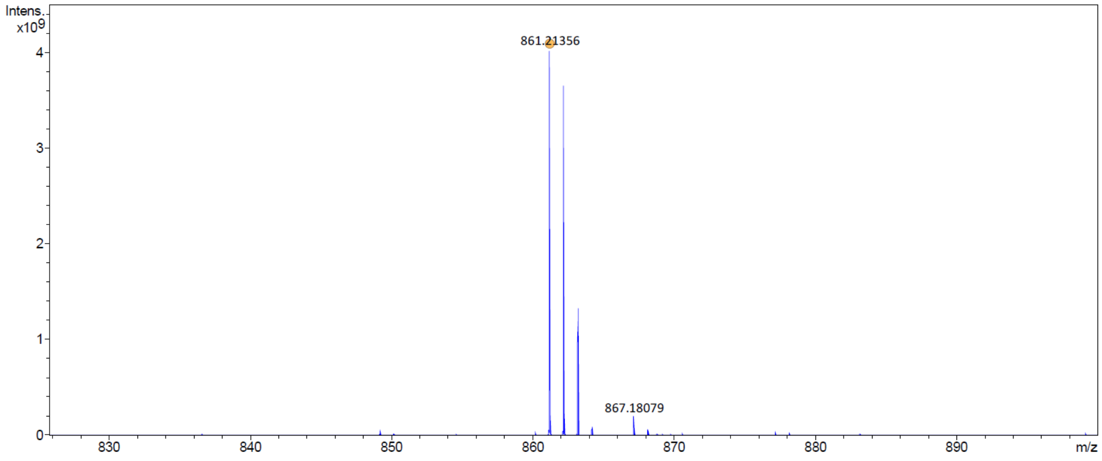

Meas. m/z	#	Ion Formula	Score	m/z	err [ppm]	Mean err [ppm]	mSigma	rdb	e⁻ Conf	N-Rule
861.213564	1	C50H42N4O2RhSi	100.00	861.212659	-1.1	1.5	142.7	33.5	even	ok

Supplementary Figure 63 HR-ESI-MS spectrum of **10b.**

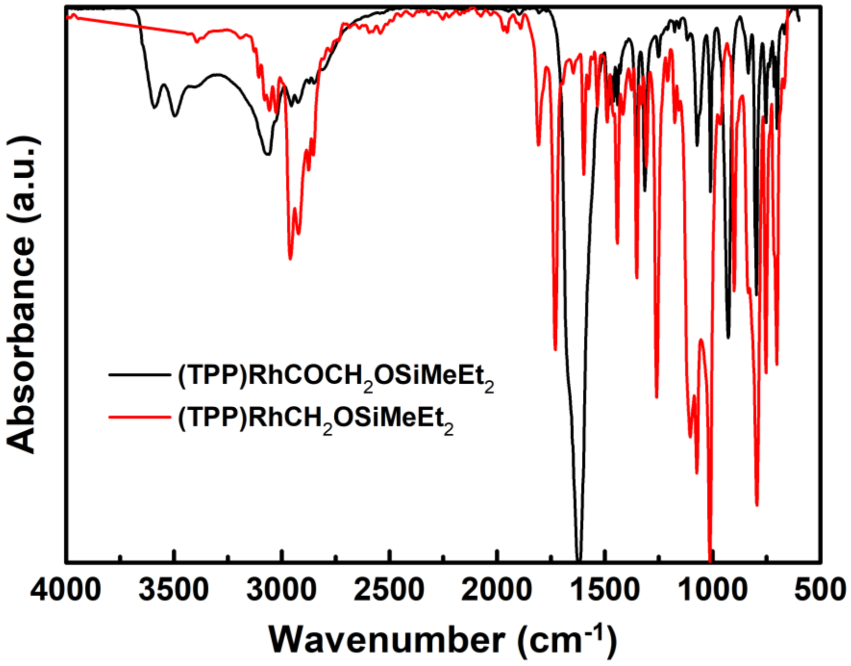

Supplementary Figure 64 FTIR spectrum of **10b**, compared against the spectrum of **6b.**

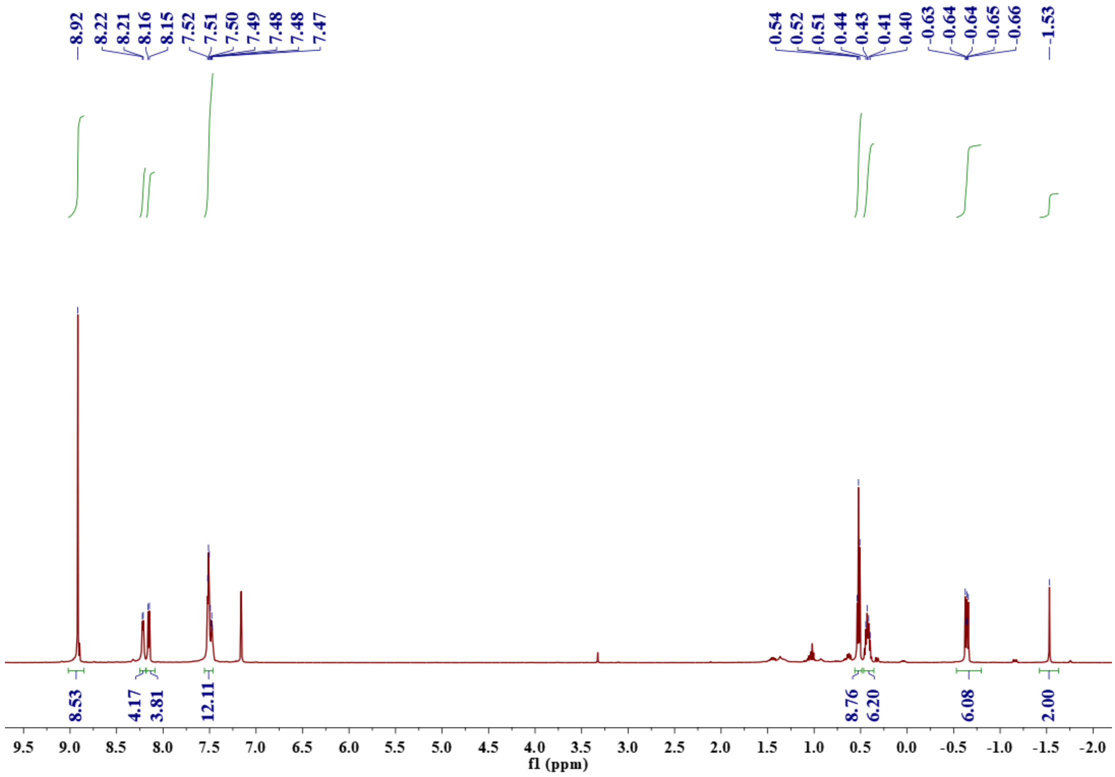

Supplementary Figure 65 ^{1}H NMR (500 MHz, C_6D_6, 25 °C) spectrum of **10c.**

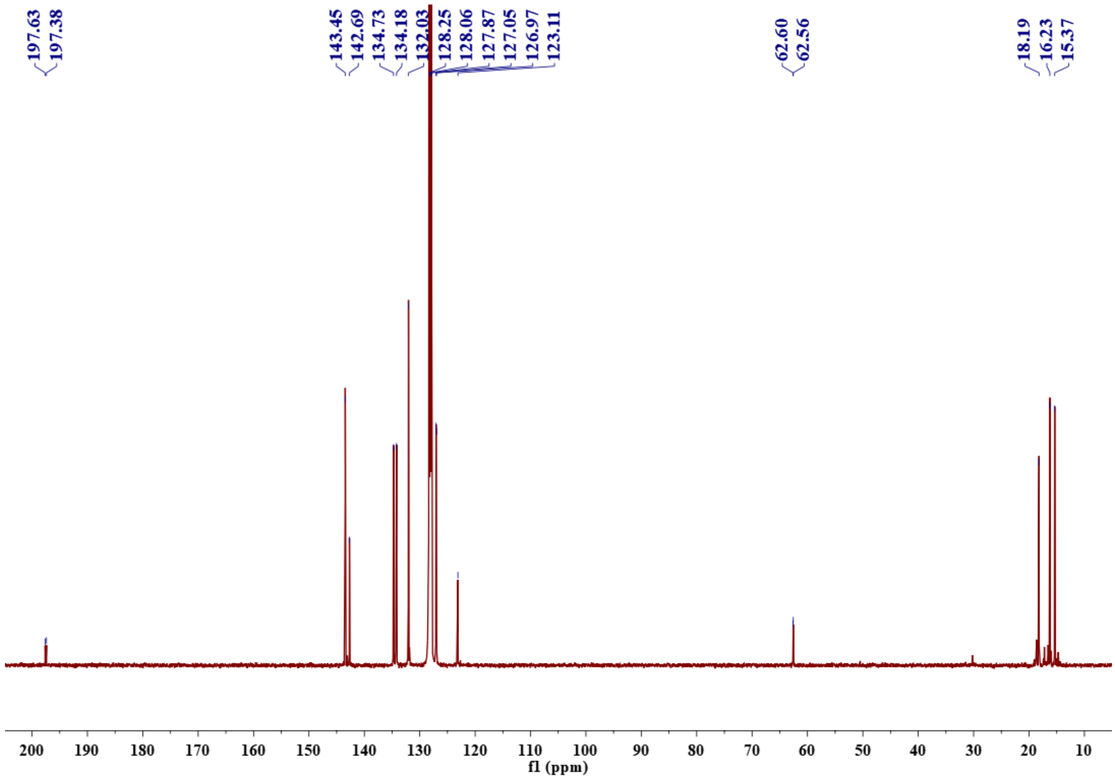

Supplementary Figure 66 ^{13}C NMR (126 MHz, C_6D_6, 25 °C) spectrum of **10c.**

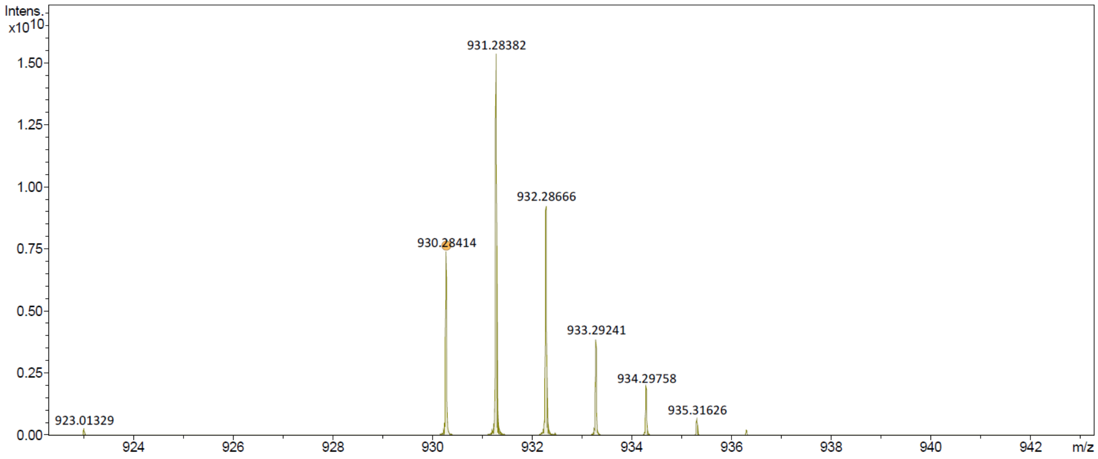

Meas. m/z	#	Ion Formula	Score	m/z	err [ppm]	Mean err [ppm]	mSigma	rdb	e^- Conf	N-Rule
930.284145	1	C55H51N4O2RhSi	100.00	930.283084	-1.1	-0.9	330.3	34.0	odd	ok

Supplementary Figure 67 HR-ESI-MS spectrum of **10c.**

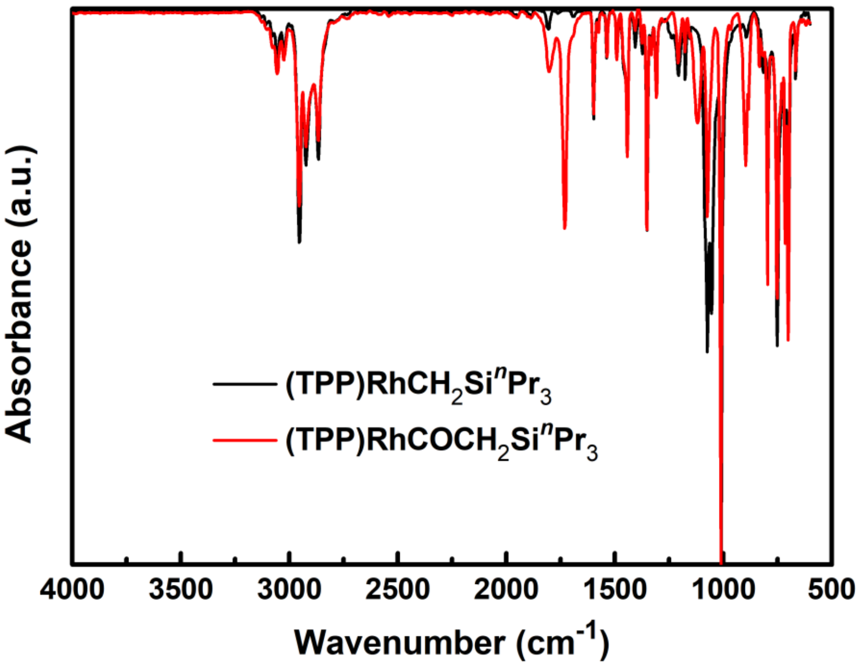

Supplementary Figure 68 FTIR spectrum of **10c**, compared against the spectrum of **6c.**

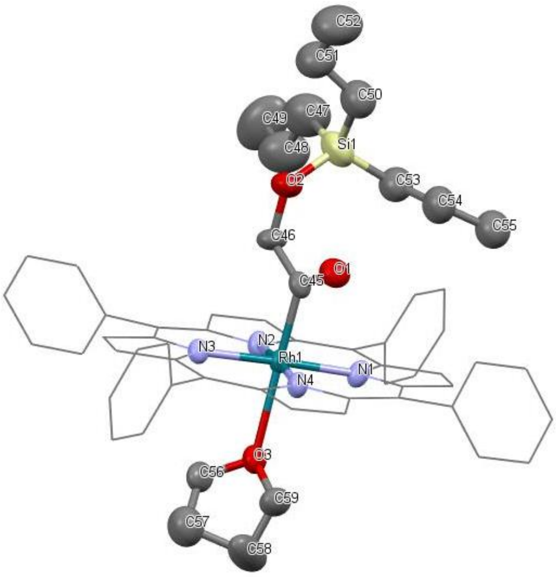

Supplementary Figure 69 X-ray crystal structure presentation of the molecular structure for **10c·THF.** Thermal ellipsoids set at 50 % probability; hydrogen atoms and some labels are omitted for clarity. Gray: carbon, yellow: silicon, red: oxygen, light blue: nitrogen, dark blue-green: rhodium.

Supplementary Table 7 Crystal data and structure refinement for **10c·THF**.

Empirical formula	C59 H59 N4 O3 Rh Si	
Formula weight	1003.10	
Temperature	173 K	
Wavelength	0.71073 Å	
Crystal system	Triclinic	
Space group	P -1	
Unit cell dimensions	a = 9.5640(8) Å	α = 94.166(18)°.
	b = 11.5743(10) Å	β = 97.061(18)°.
	c = 24.0330(19) Å	γ = 103.345(11)°.
Volume	2554.8(4) Å^3	
Z	2	

Density (calculated)	1.304 Mg/m^3
Absorption coefficient	0.406 mm^{-1}
F(000)	1048
Crystal size	0.312 x 0.235 x 0.061 mm^3
Theta range for data collection	1.717 to 25.200°.
Index ranges	-11<=h<=11, -13<=k<=13, -28<=l<=28
Reflections collected	30384
Independent reflections	9192 [R(int) = 0.1110]
Completeness to theta = 25.200°	99.9 %
Absorption correction	Semi-empirical from equivalents
Max. and min. transmission	1.000 and 0.702
Refinement method	Full-matrix least-squares on F^2
Data / restraints / parameters	9192 / 224 / 685
Goodness-of-fit on F^2	1.214
Final R indices [I>2sigma(I)]	R1 = 0.1147, wR2 = 0.1798
R indices (all data)	R1 = 0.1413, wR2 = 0.1918
Extinction coefficient	n/a
Largest diff. peak and hole	0.755 and -1.123 e.Å^{-3}

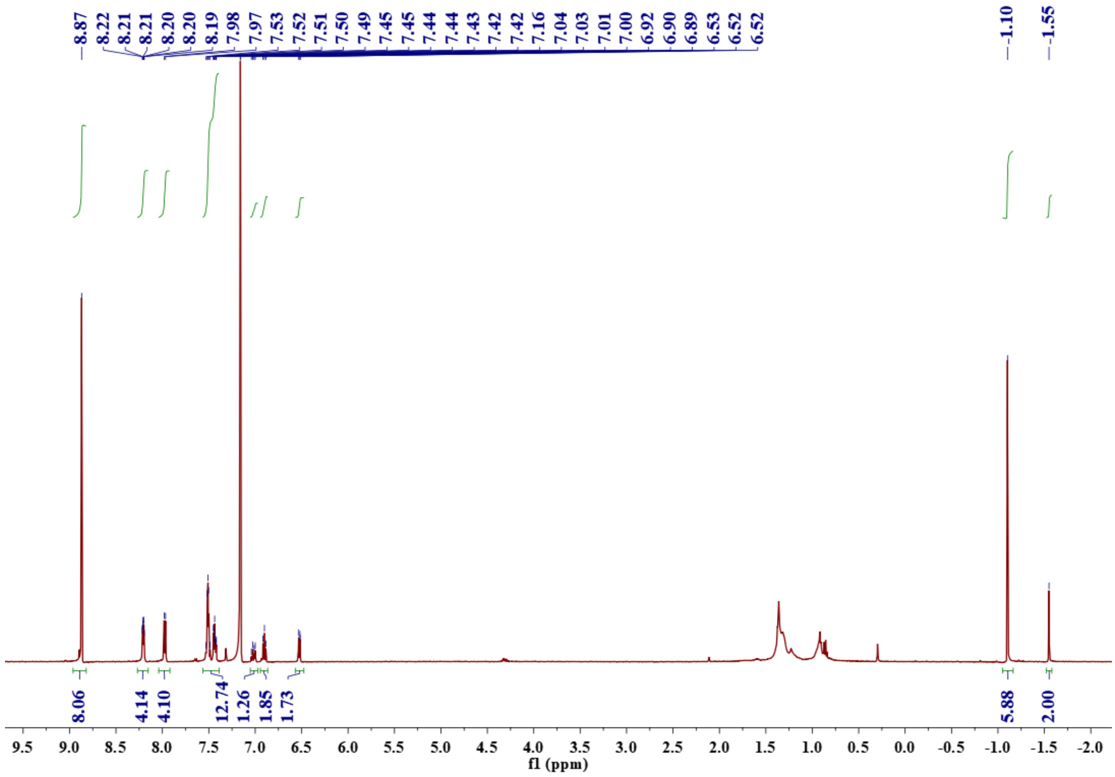

Supplementary Figure 70 ^{1}H NMR (500 MHz, C_6D_6, 25 °C) spectrum of **10d.**

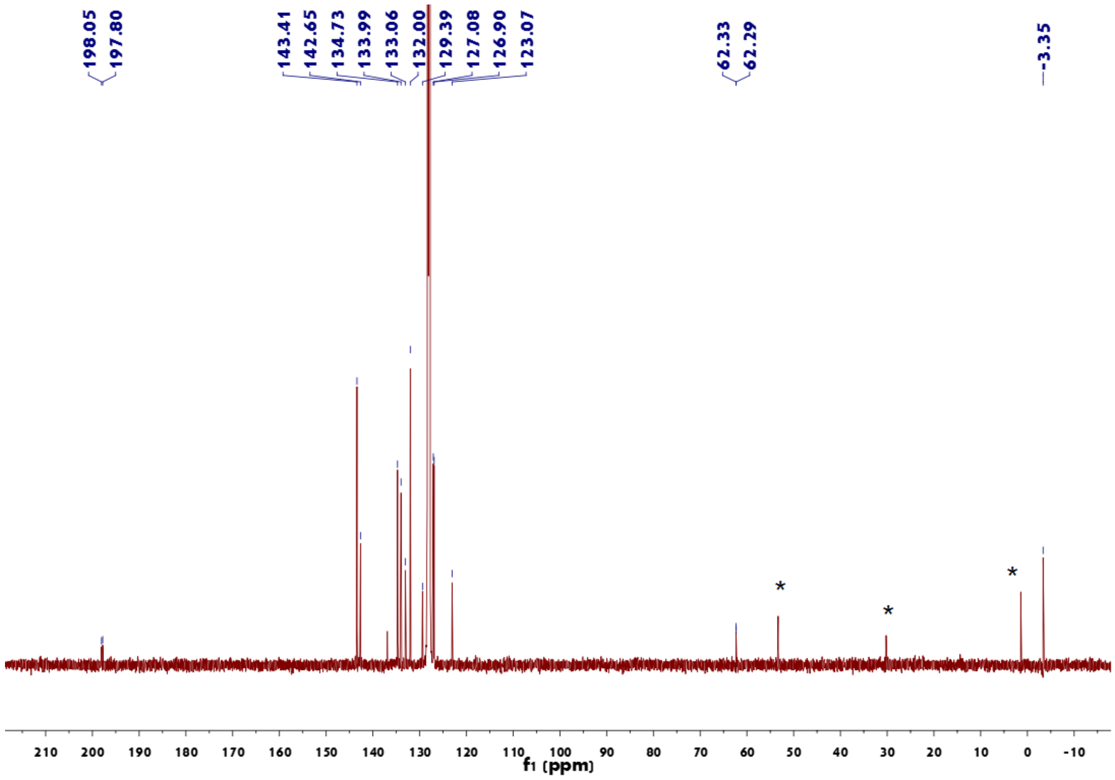

Supplementary Figure 71 ^{13}C NMR (126 MHz, C_6D_6, 25 °C) spectrum of **10d.** Signals labelled by “*” represent silicone grease and CH_2Cl_2.

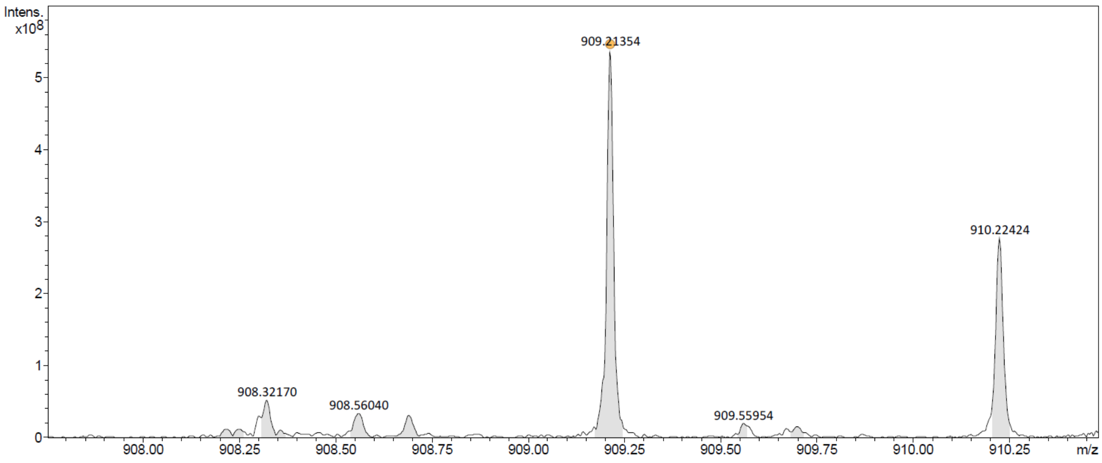

Meas. m/z	#	Ion Formula	Score	m/z	err [ppm]	Mean err [ppm]	mSigma	rdb	e⁻ Conf	N-Rule
909.213536	1	C54H42N4O2RhSi	100.00	909.212659	-1.0	552.9	78.2	37.5	even	ok

Supplementary Figure 72 HR-ESI-MS spectrum of **10d.**

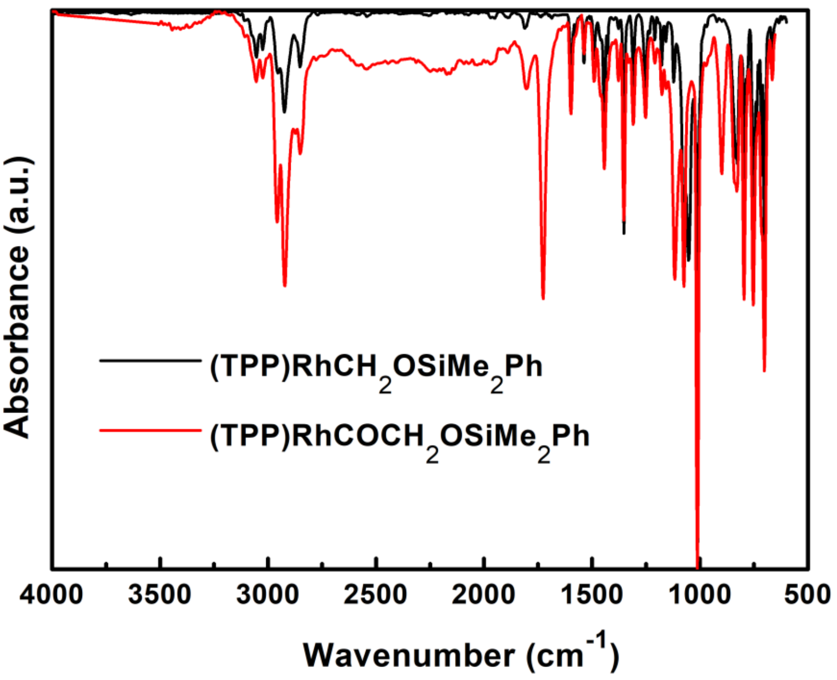

Supplementary Figure 73 FTIR spectrum of **10d**, compared against the spectrum of **6d.**

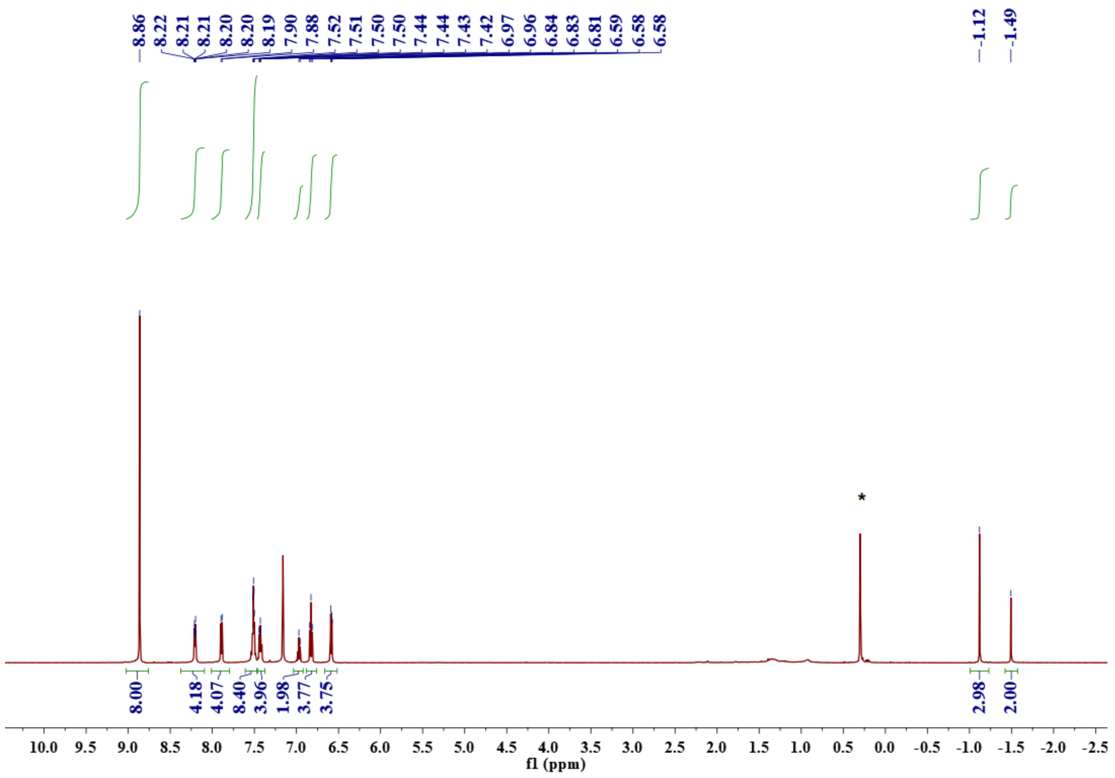

Supplementary Figure 74 ^{1}H NMR (500 MHz, C_6D_6, 25 °C) spectrum of **10e.** Signals labelled by "*" represent silicone grease.

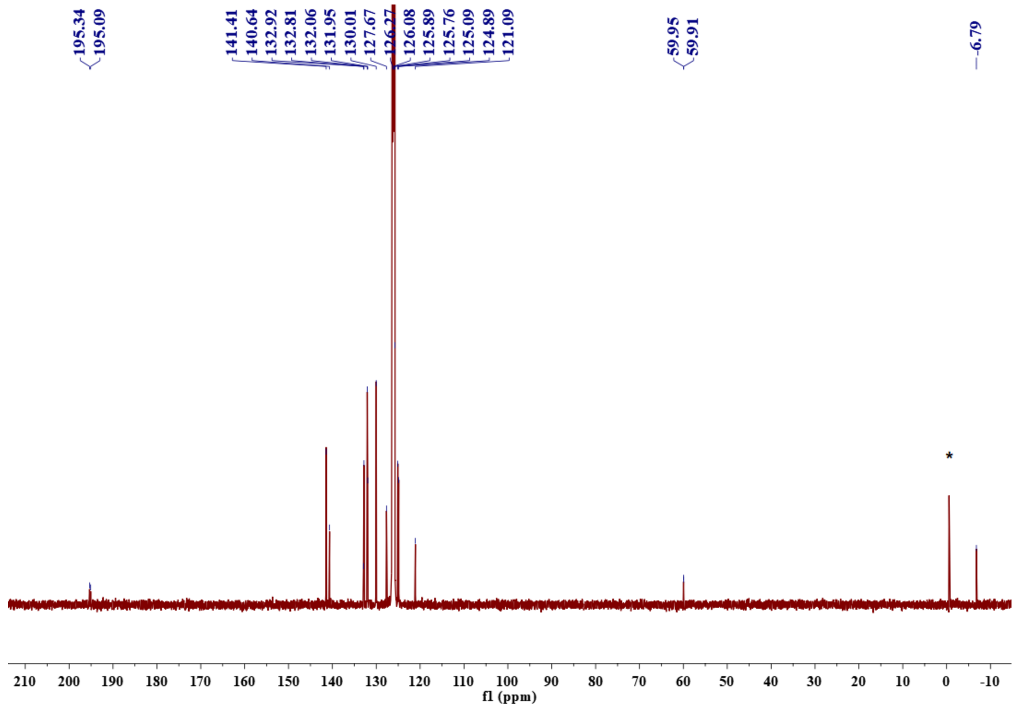

Supplementary Figure 75 ^{13}C NMR (126 MHz, C_6D_6, 25 °C) spectrum of **10e.** Signals labelled by "*" represent silicone grease.

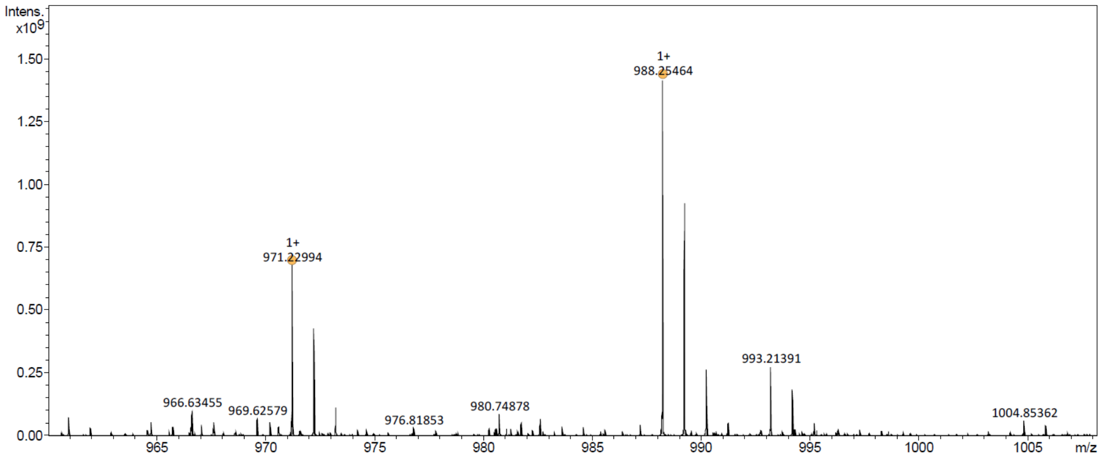

Meas. m/z	#	Ion Formula	Score	m/z	err [ppm]	Mean err [ppm]	mSigma	rdb	e⁻ Conf	N-Rule
971.229942	1	C59H44N4O2RhSi	100.00	971.228309	-1.7	-2.0	66.1	42.0	even	ok
988.254644	1	C59H47N5O2RhSi	100.00	988.254858	0.2	-2.4	55.8	41.0	even	ok

Supplementary Figure 76 HR-ESI-MS spectrum of **10e.**

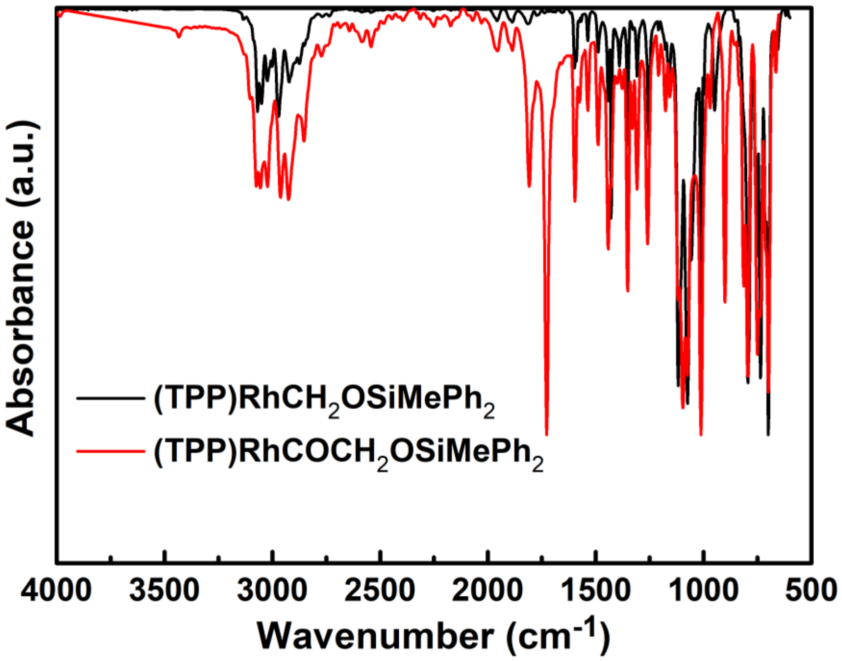

Supplementary Figure 77 FTIR spectrum of **10e**, compared against the spectrum of **6e.**

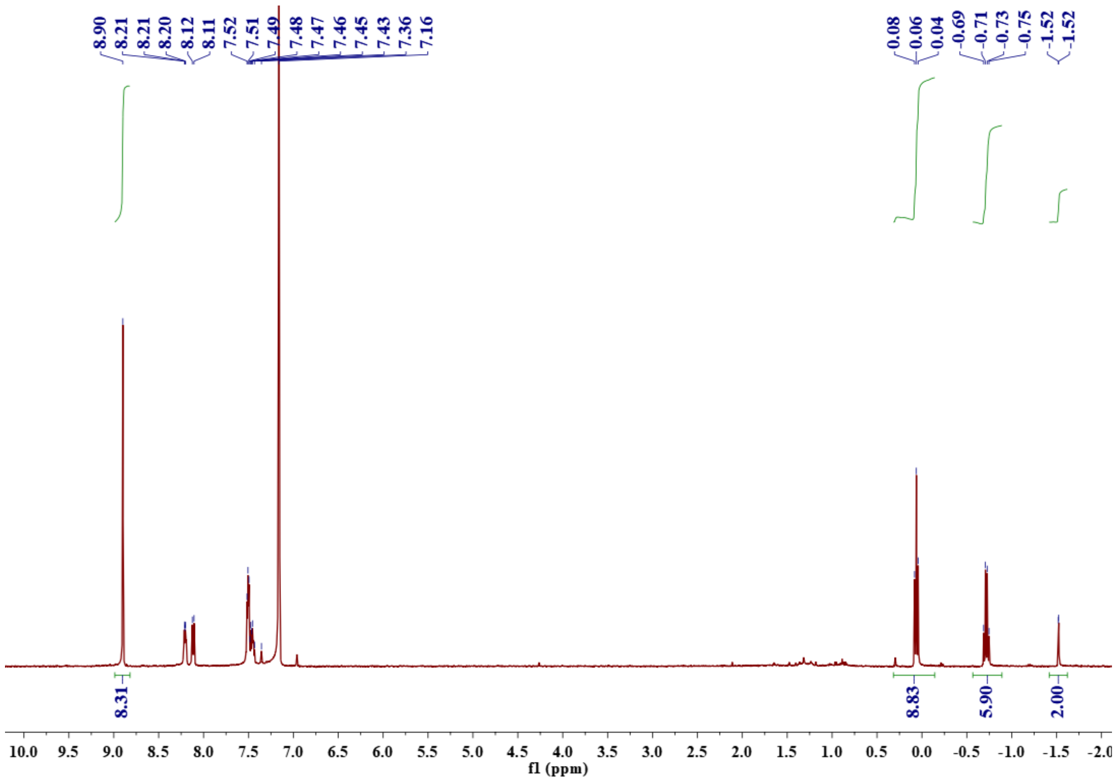

Supplementary Figure 78 ^{1}H NMR (400 MHz, C_6D_6, 25 °C) spectrum of **10f.**

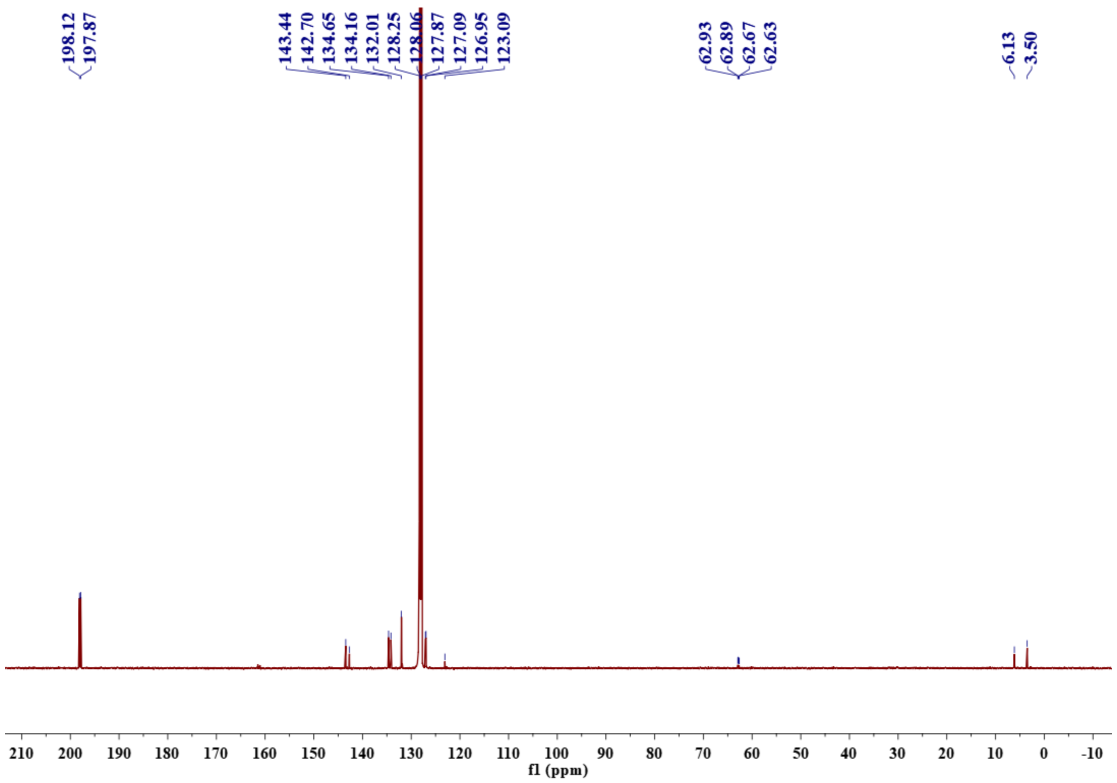

Supplementary Figure 79 ^{13}C NMR (126 MHz, C_6D_6, 25 °C) spectrum of **10f.**

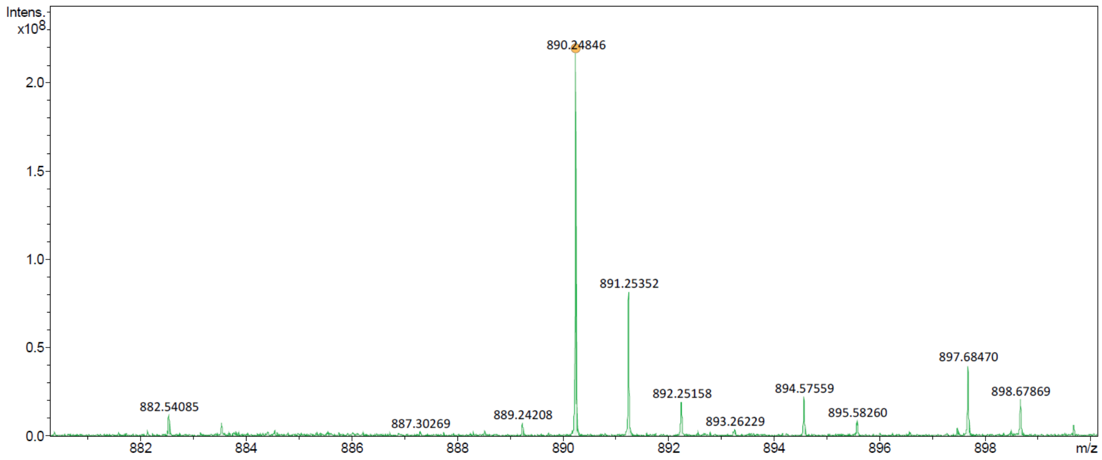

Meas. m/z	#	Ion Formula	Score	m/z	err [ppm]	Mean err [ppm]	mSigma	rdb	e⁻ Conf	N-Rule
890.248459	1	C51H46N4O2RhSi^13C	100.00	890.247314	-1.3	572.0	126.7	33.5	even	-

Supplementary Figure 80 HR-ESI-MS spectrum of **10f.**

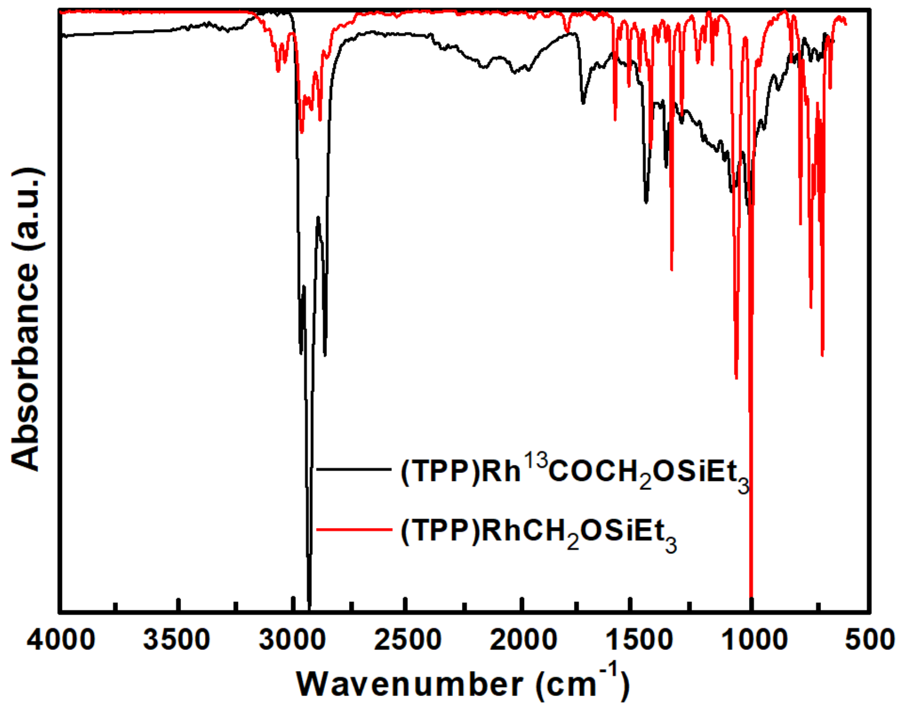

Supplementary Figure 81 FTIR spectrum of **10f**, compared against the spectrum of **6f.**

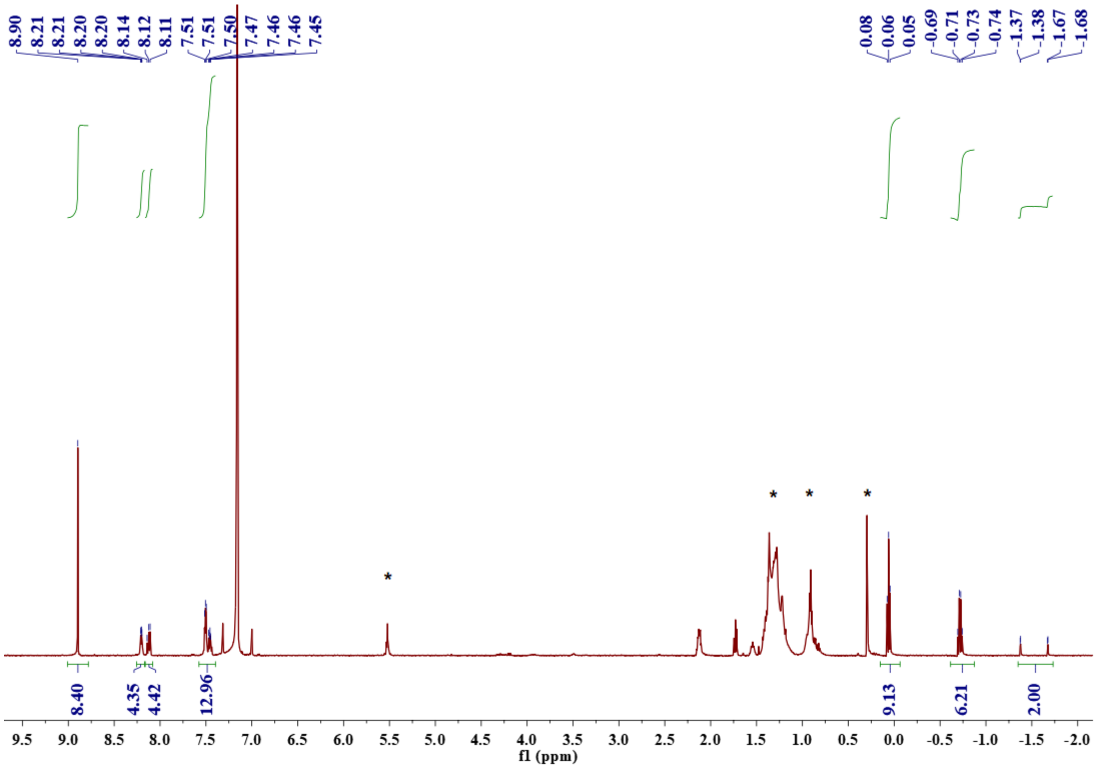

Supplementary Figure 82 ^{1}H NMR (500 MHz, C_6D_6, 25 °C) spectrum of **10g.** Signals labelled by "*" represent solvent.

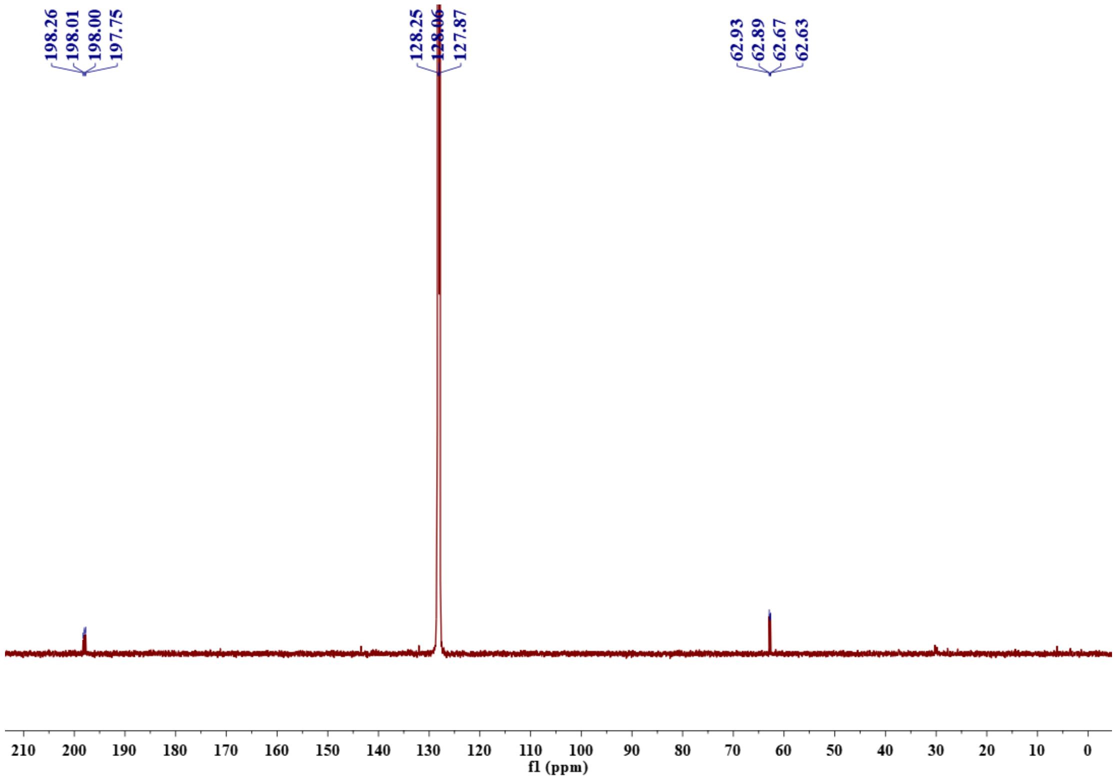

Supplementary Figure 83 ^{13}C NMR (126 MHz, C_6D_6, 25 °C) spectrum of **10g.**

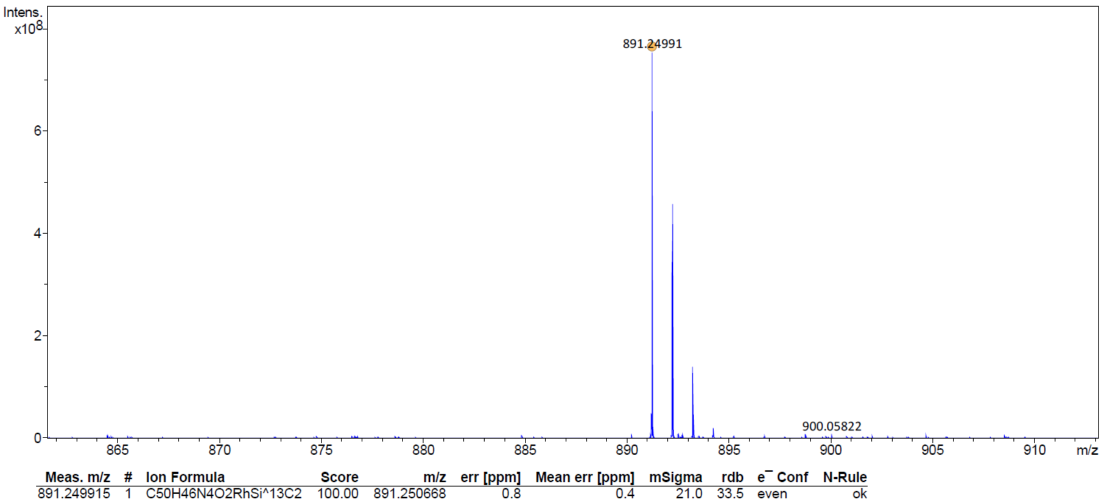

Supplementary Figure 84 HR-ESI-MS spectrum of **10g.**

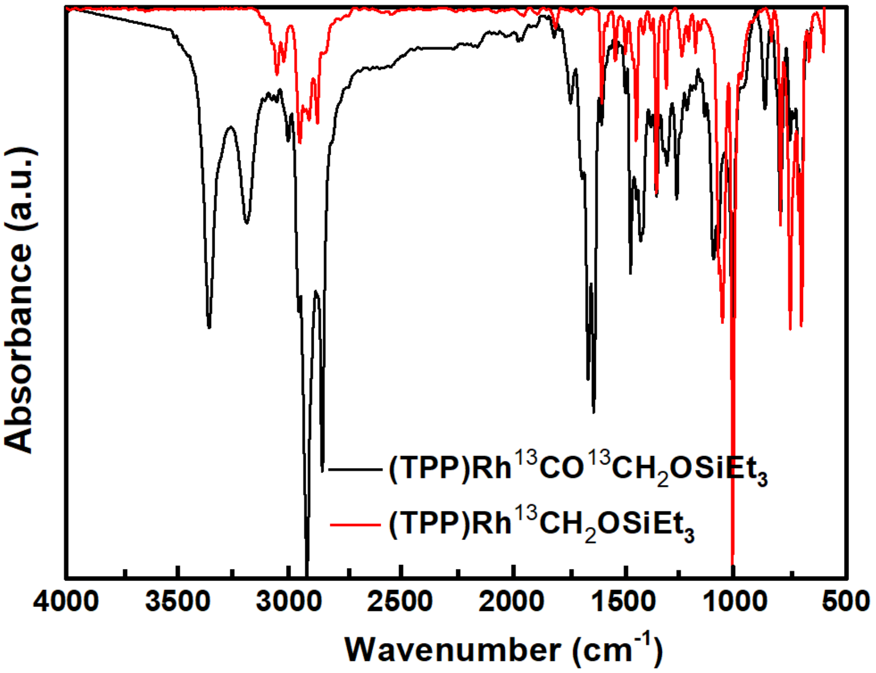

Supplementary Figure 85 FTIR spectrum of **10g**, compared against the spectrum of **6g.**

Product release and characterization of XCOCH$_2$OSiR^{1}R^{2}R^3 (X = ^{n}PrNH, TEMPO, Br)

We added 0.5 μL of *n*-propylamine to a 1 mg (TPP)RhCOCH$_2$OSi(CH$_2$CH$_3$)$_3$ solution in 300 μL of C$_6$D$_6$, followed by heating it at 60 °C for 3 h, giving ^{n}PrNHCOCH$_2$OSi(CH$_2$CH$_3$)$_3$ (**11a**) with a yield of 41 %. When the isotopically labelled reactant (TPP)Rh^{13}COCH$_2$OSi(CH$_2$CH$_3$)$_3$

was used, ^{n}PrNH^{13}COCH$_2$OSi(CH$_2$CH$_3$)$_3$ (**11b**) was produced, with a signature carbon peak at 169.98 ppm in ^{13}C NMR, and an ESI-MS spectrum consistent with the incorporation of one ^{13}C ([M+H]$^+$, 233.17601).

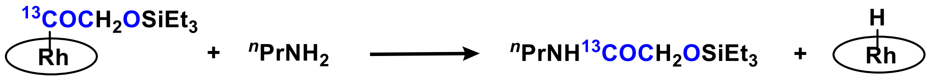

^{n}PrNHCOCH$_2$OSi(CH$_2$CH$_3$)$_3$ (11a): ^{1}H NMR (400 MHz, C$_6$D$_6$) δ (ppm): 4.08 (s, 2 H, COC$\underline{H}_2$O), 1.49-0.59 (m, 22 H, Si(CH_2CH_3)$_3$ and NHCH_2CH_2CH_3).

^{n}PrNH^{13}COCH$_2$OSi(CH$_2$CH$_3$)$_3$ (11b): ^{1}H NMR (400 MHz, C$_6$D$_6$) δ (ppm): 4.08 (d, 2 H, $^2J_{13C\text{-}H}$ = 4.9 Hz, ^{13}COC$\underline{H}_2$O), 1.10-0.40 (m, 22 H, Si(CH$_2$CH$_3$)$_3$ and NHCH$_2$CH$_2$CH$_3$). ^{13}C NMR (126 MHz, C$_6$D$_6$) δ (ppm): 169.98 (s, N^{13}COCH$_2$); HR-ESI-MS m/z calcd for C$_{10}$H$_{26}$NO$_2$Si^{13}C, [M+H]$^+$ 233.17608; found 233.17601.

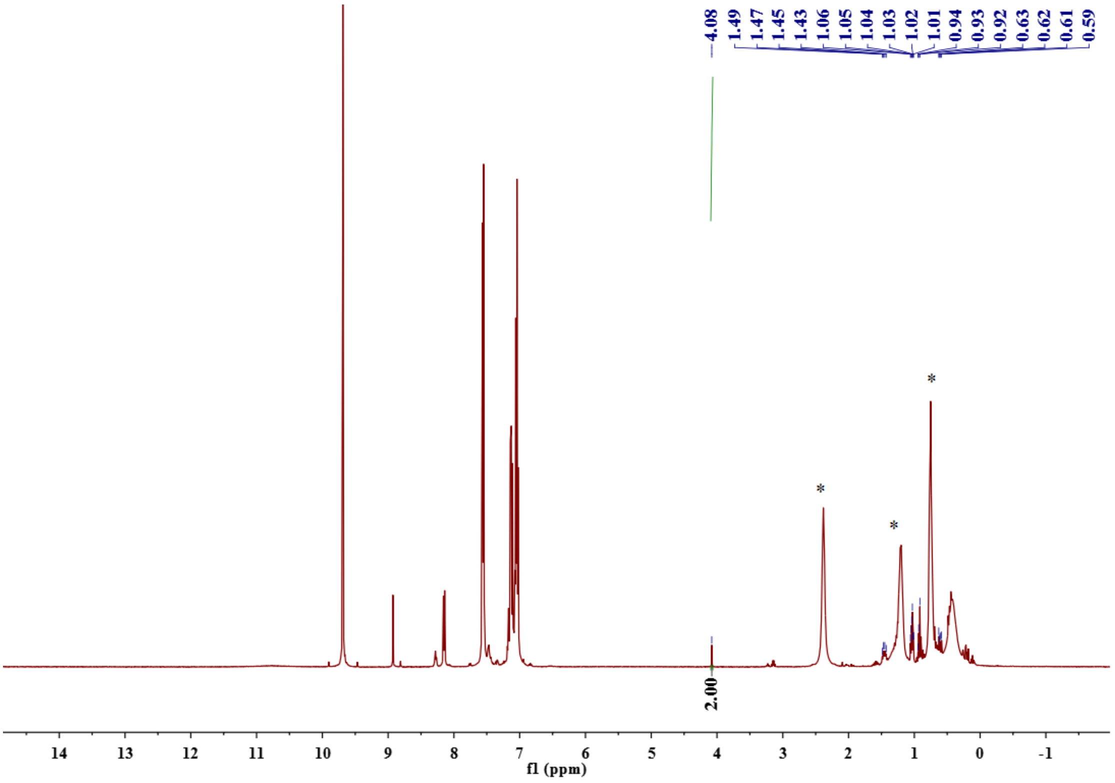

Supplementary Figure 86 ^{1}H NMR (400 MHz, C$_6$D$_6$, 25 °C) spectrum of **11a.** Signals labelled by “*” represent NH$_2$CH_2CH_2CH_3.

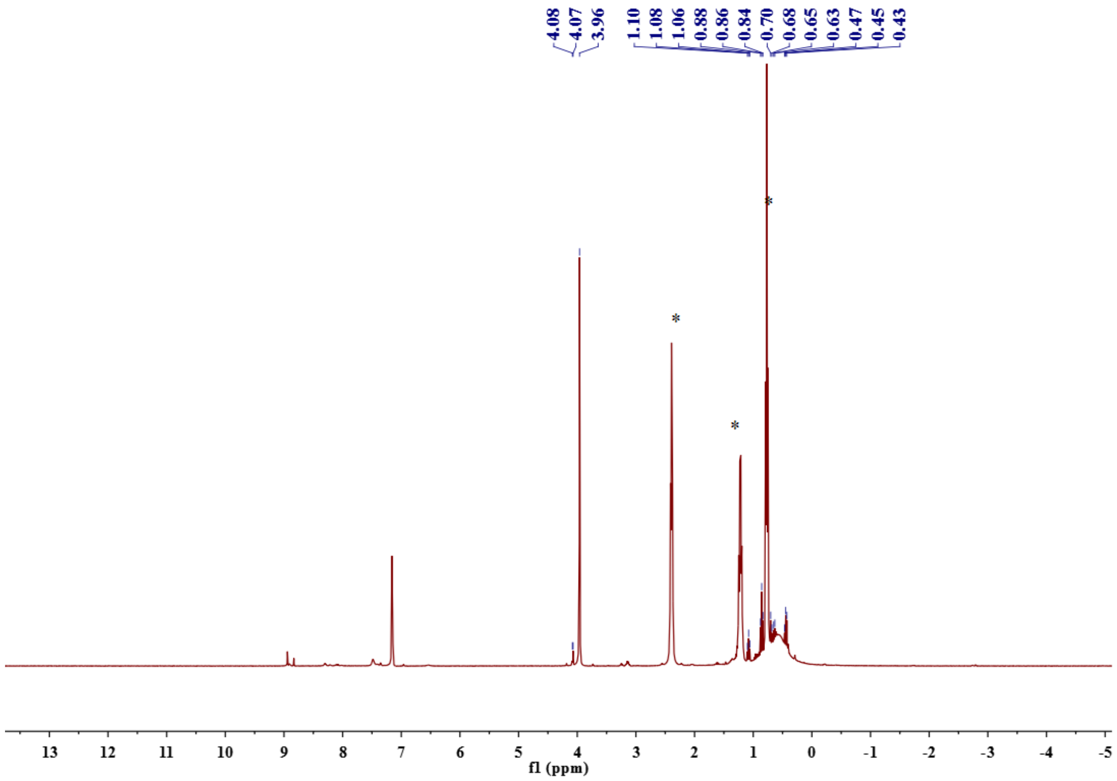

Supplementary Figure 87 ^{1}H NMR (400 MHz, C_6D_6, 25 ℃) spectrum of **11b.** Signals labelled by "*" represent $NH_2CH_2CH_2CH_3$.

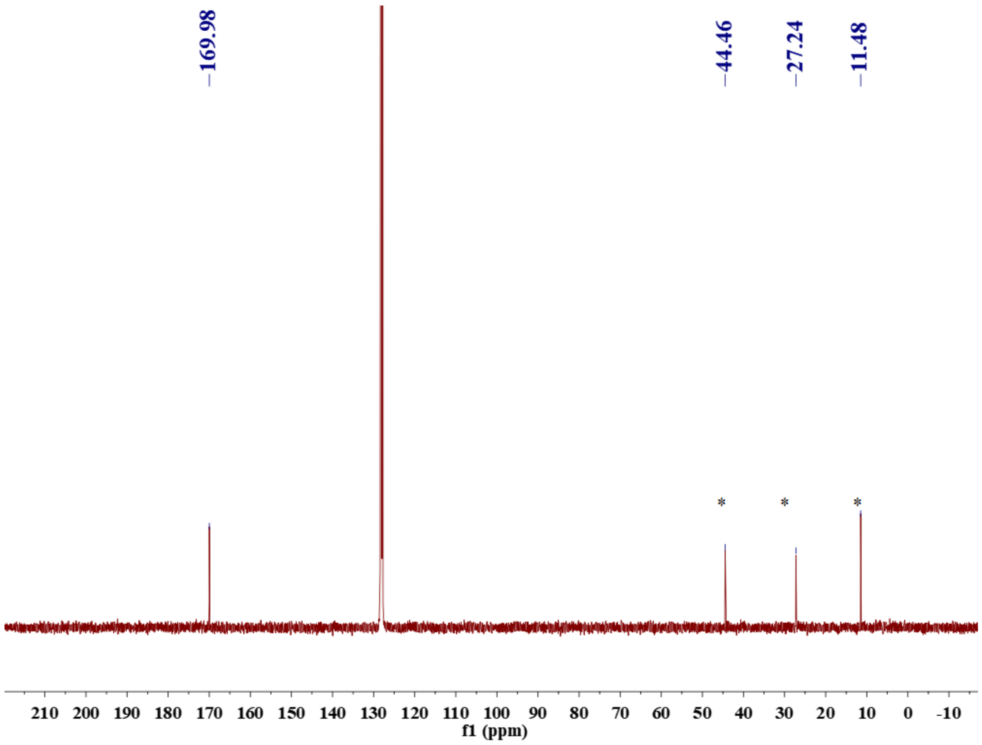

Supplementary Figure 88 ^{13}C NMR (126 MHz, C_6D_6, 25 ℃) spectrum of **11b.** Signals labelled by "*" represent $NH_2CH_2CH_2CH_3$.

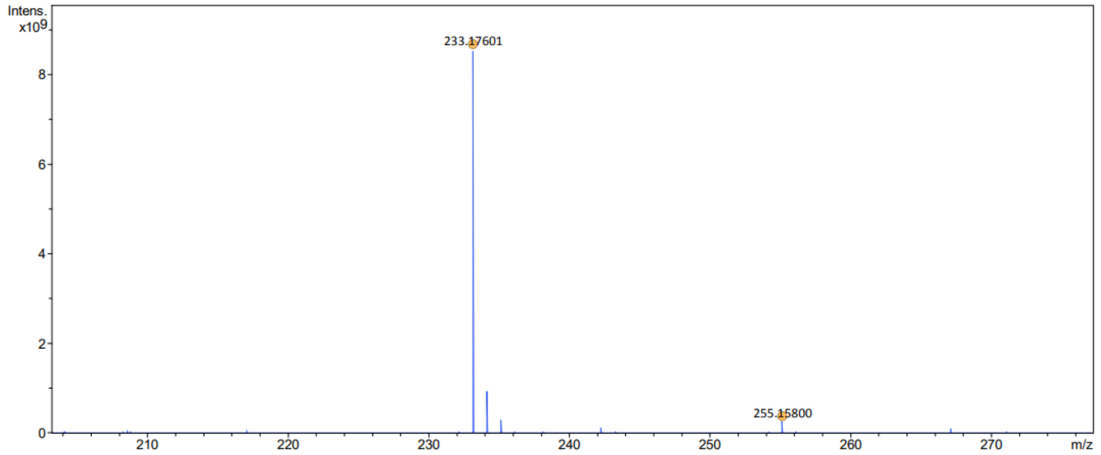

Meas. m/z	#	Ion Formula	Score	m/z	err [ppm]	Mean err [ppm]	mSigma	rdb	e⁻ Conf	N-Rule
233.176012	1	C10H26NO2Si^13C	100.00	233.176087	0.3	2.3	5.0	1.0	even	-
255.158005	1	C10H25NNaO2Si^13C	100.00	255.158031	0.1	4.2	6.2	1.0	even	-

Supplementary Figure 89 HR-ESI-MS spectrum of **11b.**

Treating 1 mg of (TPP)RhCOCH$_2$OSiR^{1}R^{2}R^3 with excess TEMPO (~ 10 eq.) in a vacuum NMR tube, then exposing it to visible light led to the formation of TEMPO-COCH$_2$OSiR^{1}R^{2}R^3 as the major release product, as well as TEMPO-Rh(TPP). When we used (TPP)RhCOCH$_2$OSiEt$_3$ (**10a**), the yield of TEMPO-COCH$_2$OSiEt$_3$ (**12**) was ~ 85 %.

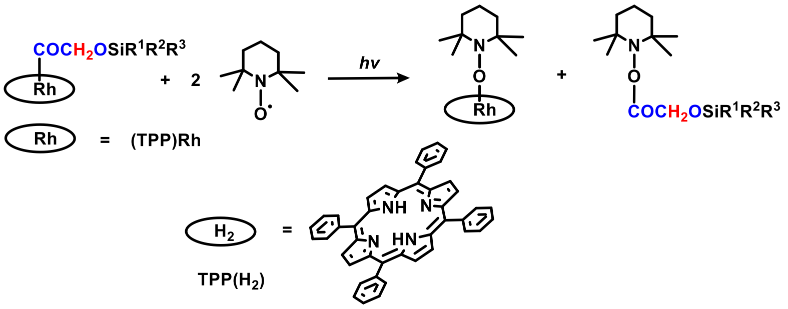

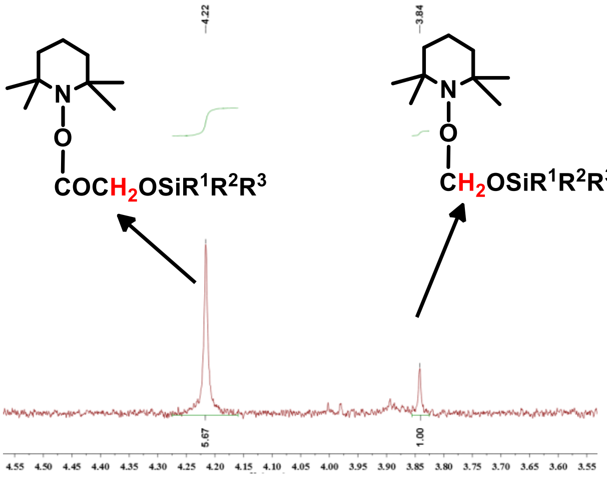

Supplementary Figure 90 ^{1}H NMR spectrum of **12** and **9.**

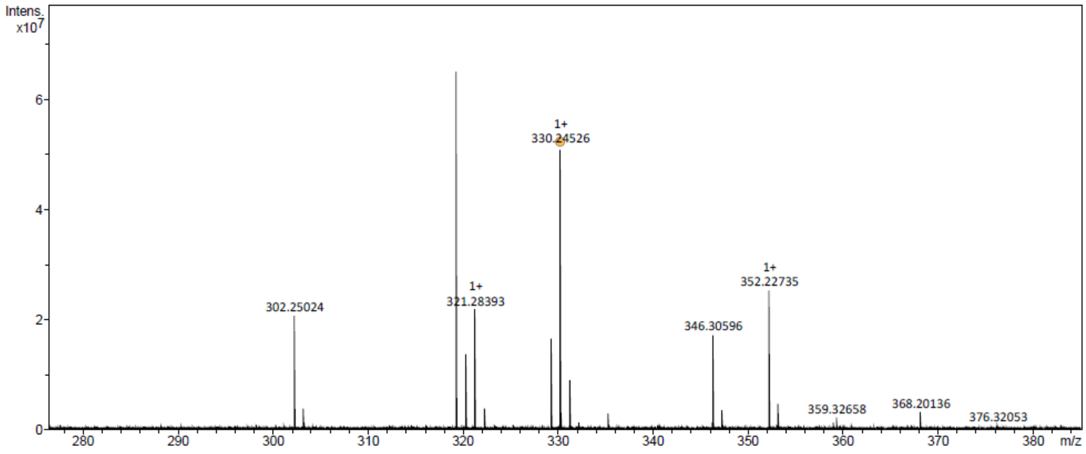

Meas. m/z	#	Ion Formula	Score	m/z	err [ppm]	Mean err [ppm]	mSigma	rdb	e⁻ Conf	N-Rule
330.245259	1	C17H36NO3Si	100.00	330.245897	1.9	1.2	5.9	2.0	even	ok

Supplementary Figure 91 HR-ESI-MS spectrum of **12**。

Adding 2 μL of $BrCCl_3$ to a 1 mg (TPP)RhCOCH$_2$OSi(CH$_2$CH$_3$)$_3$ (**10a**) solution in 300 μL of C_6D_6 and irradiation by light at 6 ^{o}C for 2 h resulted in the formation of (TPP)RhBr (**2**) and BrCOCH$_2$OSi(CH$_2$CH$_3$)$_3$ (**13**) with near quantitative NMR yield.

BrCOCH$_2$OSi(CH$_2$CH$_3$)$_3$ (13): ^{1}H NMR (400 MHz, C_6D_6) δ (ppm): 4.03 (s, BrCOC*H*$_2$OSi(CH$_2$CH$_3$)$_3$), 0.83 (t, BrCOCH$_2$OSi(CH$_2$C*H*$_3$)$_3$, $^{3}J_{H\text{-}H}$ = 8.0 Hz), 0.38 (BrCOCH$_2$OSi(C*H*$_2$CH$_3$)$_3$, $^{3}J_{H\text{-}H}$ = 8.0 Hz)). HR-ESI-MS m/z calcd for (C*H*$_3$CH$_2$)$_3$SiOCH$_2$COO$^-$, $C_8H_{17}O_3Si$, [M] 189.09524; found 189.09497. We were unable to obtain ESI-MS spectra of the unchanged compound (CH$_3$CH$_2$)$_3$SiOCH$_2$COBr, owing to its sensitivity towards water; however, the ESI-MS characterization of the corresponding carboxylate still lends support to the acyl bromide structure.

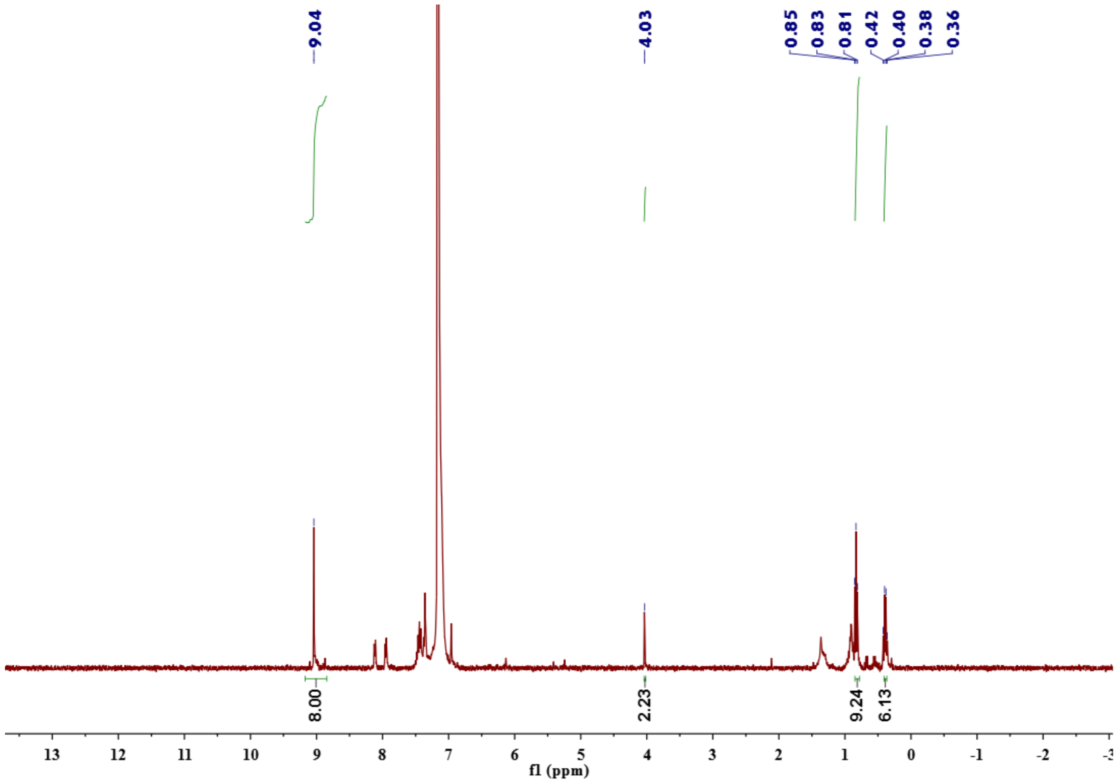

Supplementary Figure 92 ^{1}H NMR (400 MHz, C_6D_6, 25 ℃) spectrum of **13.**

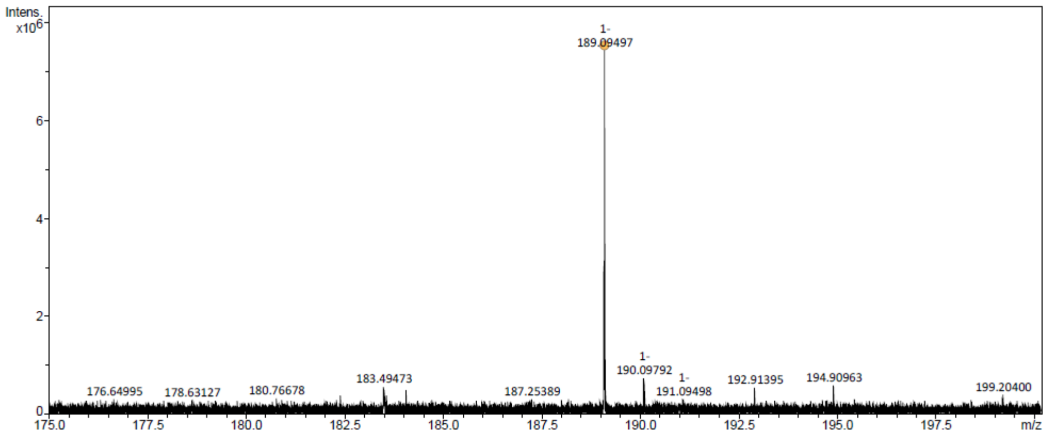

Meas. m/z	#	Ion Formula	Score	m/z	err [ppm]	Mean err [ppm]	mSigma	rdb	e⁻ Conf	N-Rule
189.094973	1	C8H17O3Si	100.00	189.095245	1.4	-2.7	21.7	1.0	even	ok

Supplementary Figure 93 HR-ESI-MS of $\mathbf{(CH_3CH_2)_3SiOCH_2COO^-}$**.**

Supplementary Discussion

In this section, we provide detailed results and discussions of the ground state and excited state potential energy surfaces of **6a**, **10a** and **6a-CO**, which shed light on their photolysis pathways.

Supplementary Table 8. Calculated equilibrium Rh-C bond lengths and Rh-C bond dissociation free energies (BDFEs) of **6a**, **10a** and **6a-CO** at the S_0, T_1 and S_1 states.

Bond	Bond length (Å)			BDFE (kcal/mol)		
	S_0	T_1	S_1	S_0	T_1	S_1
**6a**, Rh-C(alkyl)	2.020	2.015	2.013	38.6	2.5	-16.2
**10a**, Rh-C(alkyl)	1.961	1.951	1.948	50.2	12.5	-5.1
**6a-CO**, Rh-C(alkyl)	2.050	2.083	2.350	33.9	1.0	-12.3
**6a-CO**, Rh-C(carbonyl)	2.062	1.999	1.847	1.5	4.7	10.1

The ground state and excited state potential energy curves of **6a** are shown in **Supplementary Figure 94**. The ground state potential energy curve is purely uphill throughout the bond length range studied, with a cumulative energy increase of 1.96 eV (45.2 kcal/mol), which suggests that thermal Rh-C bond homolysis is not viable under the experimental conditions. The lack of a barrier proves that the recombination of [(TPP)Rh(II)]$^{\bullet}$ and $^{\bullet}CH_2OSi(CH_2CH_3)_3$ is barrierless.

Upon photoexcitation of the Q band (534 nm), the molecule enters a region with many triplet states, to which the molecule may undergo intersystem crossing (ISC). However, only three energetically accessible triplet states lead to Rh-C bond cleavage. The lowest one (labelled ^{3}(σ-σ*)) is purely downhill and leads to the ground state [(TPP)Rh(II)]$^{\bullet}$ and $^{\bullet}CH_2OSi(CH_2CH_3)_3$ radicals; it crosses the Q band energy curves at a Rh-C bond length of ~2.3 Å, and an energy input of 0.23 eV (5.3 kcal/mol) is required to reach the crossing point. The other two are almost degenerate and also lead to the [(TPP)Rh(II)]$^{\bullet}$ and $^{\bullet}CH_2OSi(CH_2CH_3)_3$ radicals, but where the [(TPP)Rh(II)]$^{\bullet}$

radical is in its (d_π-d_{z2}) excited state, i.e. its single electron resides on the d_{xz} or d_{yz} orbitals instead of the d_{z2} orbital; although they do not cross the Q band energy curves, they approach the Q band energy curves quite closely around a Rh-C bond length of 2.4 Å, so fast ISC can also be expected. The lowest dissociative singlet state, corresponding to the heterolysis pathway yielding $[(TPP)Rh(I)]^-$ and $^+CH_2OSi(CH_2CH_3)_3$, also crosses the energy curves of the Q band states, but at a longer bond length (~2.6 Å) and therefore requiring a much higher energy (0.63 eV, 14.6 kcal/mol). We therefore conclude that exciting the Q band leads to facile Rh-C bond homolysis (consistent with the experimental TEMPO trapping studies) instead of heterolysis.

The case of Soret band excitation (423 nm) is more complex. Here the aforementioned homolysis pathways are still accessible, since the Soret band is connected to the dissociative triplet states through a dense manifold of singlet and triplet states. The heterolysis pathway also becomes energetically accessible: the Soret band states can undergo internal conversion (IC) to the heterolysis state at a Rh-C bond length of around 2.1 Å, and crossing an energy barrier of 0.25 eV (5.8 kcal/mol) at 2.8 Å would lead to Rh-C bond heterolysis. However, before the Rh-C bond elongates beyond the barrier, the heterolysis energy curve would meet the Q band energy curves at 2.6 Å, at which point ultrafast IC to the Q band states can be expected. Thus, although Soret band excitation provides sufficient energy for Rh-C bond heterolysis, homolysis pathways are expected to be even faster and dominate the product distribution.

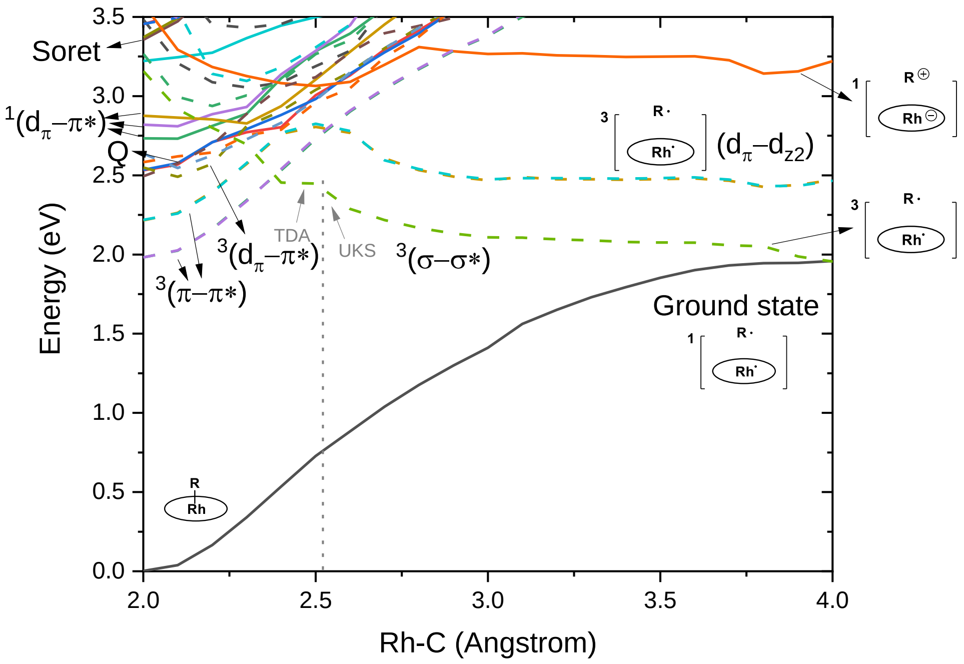

Supplementary Figure 94 Singlet (solid lines) and triplet (dashed lines) energies of structures along the ground state Rh-C bond relaxed scan trajectory of **6a** (R = $CH_2OSi(CH_2CH_3)_3$), plotted as a function of Rh-C bond distance. The compositions of selected states are given.

The ground state and excited state potential energy curves of **10a** are shown in **Supplementary Figure 95**. The ground state Rh-C bond homolysis is again purely uphill and takes more energy (2.36 eV, 54.4 kcal/mol) than **6a**, the latter of which attributed to the π back-donation of the Rh d orbitals to the carbonyl group of **10a**. The lack of a barrier proves that the recombination of $[(TPP)Rh(II)]^{\bullet}$ and $^{\bullet}COCH_2OSi(CH_2CH_3)_3$ is barrierless.

The photolysis pathways of **10a** are similar to those of **6a**, except that the heterolysis pathway is no longer energetically accessible even with Soret band excitation, since the $^{\bullet}COCH_2OSi(CH_2CH_3)_3$ radical is electron deficient and thus much less likely to form the corresponding cation than the $^{\bullet}CH_2OSi(CH_2CH_3)_3$ radical. Thus, photolysis of **10a** should lead to exclusively Rh-C bond homolysis. The lowest energy pathway for the Rh-C bond homolysis of Q band-excited **10a** is by an initial ISC to the $^3(d_\pi\text{-}\pi^*)$ state at a Rh-C bond length of ~2.2 Å, followed by internal conversion to the $^3(\sigma\text{-}\sigma^*)$ state at a bond length of ~2.3 Å, with a total energy

barrier of 0.37 eV (8.4 kcal/mol). As this is larger than the energy cost for the Q band-excited **6a** to cross into the dissociative triplet state (5.3 kcal/mol), photolysis of **10a** is probably more difficult than **6a** when irradiating the Q band, consistent with the larger Rh-C BDFE of **10a** compared to **6a** in both the S_0 and S_1 states (**Supplementary Table 8**). When irradiating the Soret band of **10a**, however, the dissociative state $^3(\sigma\text{-}\sigma^*)$ can be reached by a series of downhill IC and ISC processes due to the large number of singlet and triplet states available, which is expected to lead to very facile Rh-C bond homolysis.

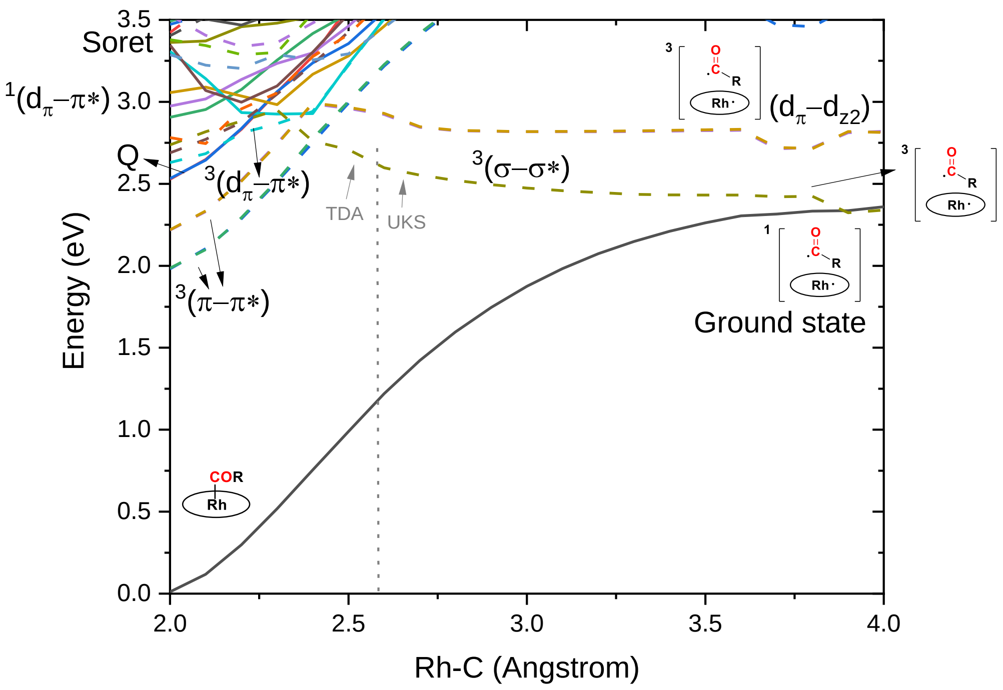

Supplementary Figure 95 Singlet (solid lines) and triplet (dashed lines) energies of structures along the ground state Rh-C bond relaxed scan trajectory of **10a** (R = $CH_2OSi(CH_2CH_3)_3$), plotted as a function of Rh-C bond distance. The compositions of selected states are given.

The ground state and excited state potential energy curves of **6a-CO** (where the Rh-C(alkyl) bond is stretched and the Rh-CO bond length is allowed to relax) are shown in **Supplementary Figure 96**. Again, the ground state potential energy curve is purely uphill, which shows that the recombination of $^{\bullet}CH_2OSi(CH_2CH_3)_3$ with $[(TPP)Rh(CO)]^{\bullet}$ to yield **6a-CO** is barrierless. While

the energy cost of Rh-C bond homolysis (1.72 eV, 39.7 kcal/mol) is smaller than those of **6a** and **10a**, it is still prohibitive for room-temperature reactions, suggesting that thermal homolysis of **6a-CO** is negligible, which is consistent with our BDFE results (**Supplementary Table 8**).

Due to the extensive mixing of the porphyrin a_{2u}-like π orbital with the σ(Rh-C(alkyl)) orbital (**Fig. 7**), the Q band energy curves acquire some σ(Rh-C(alkyl))-π* character and therefore appear flatter compared to those of **6a** and **10a**, i.e. they have much lower energies than the Q band energy curves of **6a** and **10a** for long Rh-C(alkyl) bond lengths. The crossing point with the ^{3}(σ-σ*) curve is now around 2.5 Å, requiring an energy cost of only 0.20 eV (4.5 kcal/mol) to reach. This is slightly smaller than the corresponding energy required by **6a** (5.3 kcal/mol). Our present results thus suggest that when the Q band is irradiated, the photolysis of **6a-CO** is probably easier than that of **6a**, and even more so than **10a**.

With Soret band irradiation, the situation is similar to **10a**, i.e. the ^{3}(σ-σ*) state can be reached by a series of barrierless or almost barrierless IC and ISC processes, while no energetically accessible heterolysis pathway is available. Therefore we expect that the Soret band-excited **6a-CO** undergoes very facile Rh-C homolysis, similar to **6a** and **10a**.

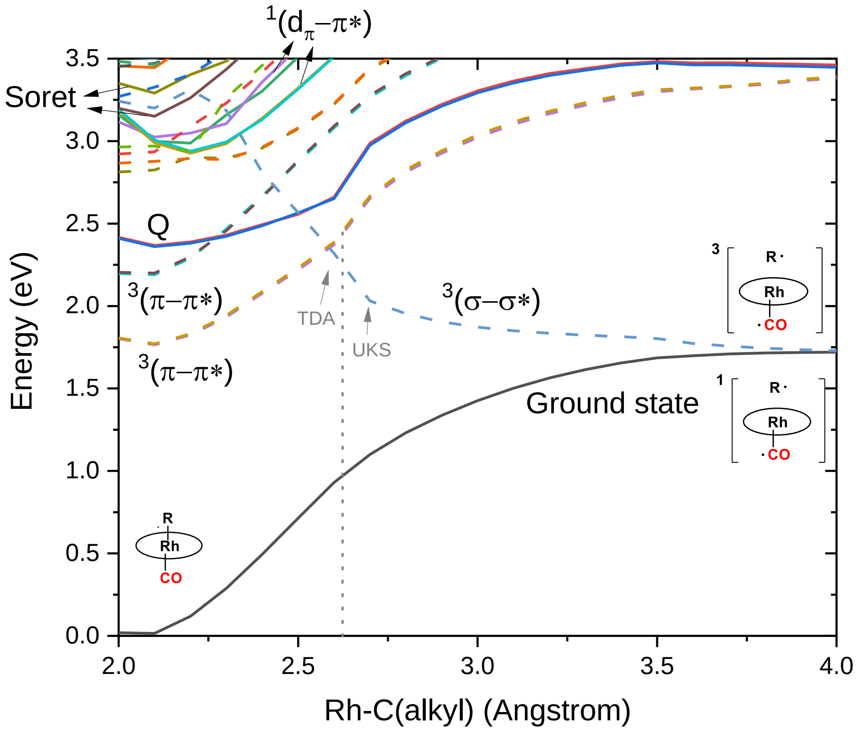

Supplementary Figure 96 Singlet (solid lines) and triplet (dashed lines) energies of structures along the ground state Rh-C(alkyl) bond relaxed scan trajectory of **6a-CO** (R = $CH_2OSi(CH_2CH_3)_3$), plotted as a function of Rh-C(alkyl) bond distance. The compositions of selected states are given.

Finally, the ground state and excited state potential energy curves of **6a-CO** (where the Rh-CO bond is stretched and the Rh-C(alkyl) bond length is allowed to relax) are shown in **Supplementary Figure 97**. The ground state potential energy curve is purely uphill, suggesting no barrier for the coordination of CO to **6a**; moreover, a very small energy increase (0.35 eV, 8.1 kcal/mol) is observed, in accord with the small BDFE (1.5 kcal/mol, **Supplementary Table 8**) and suggests that the CO ligand of **6a-CO** can easily dissociate at room temperature in a reversible fashion.

Consistent with the computed BDFEs (**Supplementary Table 8**), the Rh-CO bond becomes stronger in not only the S_1 and T_1 states, but all other π-π* states in the 0~3.5 eV window. As there is no state below the Q band for which the Rh-CO bond energy is lower than that of S_0, Q band excitation of **6a-CO** is not expected to lead to CO dissociation. However, the Soret band excited **6a-CO** possesses many readily accessible IC and ISC pathways to the singlet or triplet (d_π-π*) states, where the reduction of d_π occupation numbers reduces the π back-bonding of Rh to the CO ligand and therefore significantly weakens the Rh-CO bond. Thus, photolytic CO dissociation of **6a-CO** is possible with Soret band irradiation. Nevertheless, the CO dissociation leaves the product **6a** in its (d_π-π*) state, which is amenable to Rh-C(alkyl) bond homolysis, regardless of its spin multiplicity (**Supplementary Figure 94**). Therefore, even if Soret band excitation of **6a-CO** leads to a loss of the CO ligand, the Rh-C(alkyl) bond may still be able to break without needing to absorb a second photon.

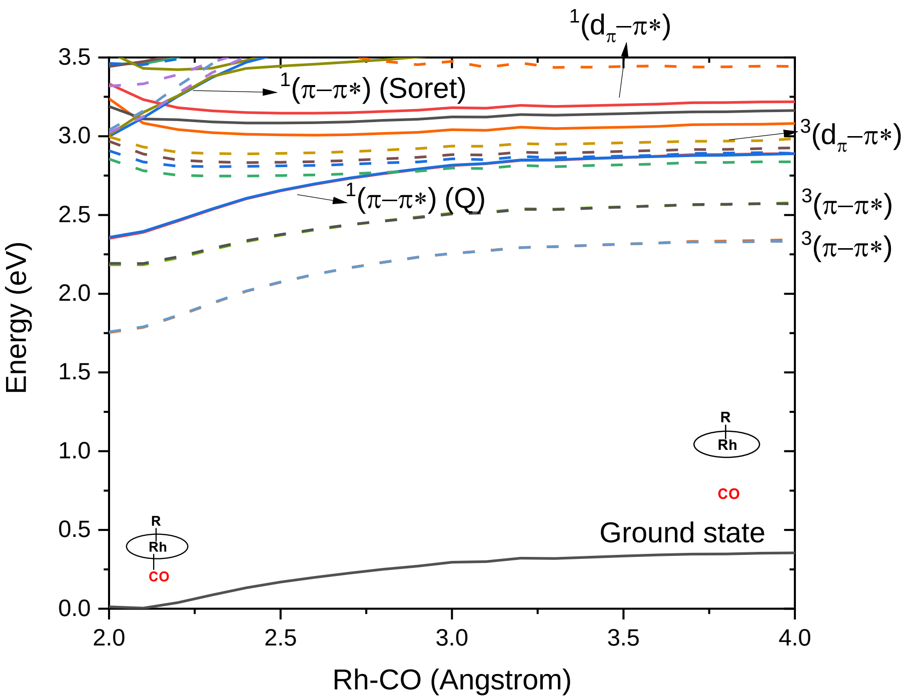

Supplementary Figure 97 Singlet (solid lines) and triplet (dashed lines) energies of structures along the ground state Rh-CO bond relaxed scan trajectory of **6a-CO** (R = $CH_2OSi(CH_2CH_3)_3$), plotted as a function of Rh-CO bond distance. The compositions of selected states are given.

Supplementary References

1. F. Neese, Software update: The ORCA program system—Version 5.0. *WIREs Comp. Mol. Sci.* **12**, e1606 (2022).
2. C. Adamo, V. Barone, Toward reliable density functional methods without adjustable parameters: The PBE0 model. *J. Chem. Phys.* **110**, 6158-6170 (1999).
3. (a) W. Kutzelnigg, W. Liu, Quasirelativistic theory equivalent to fully relativistic theory. *J. Chem. Phys.* **123**, 241102 (2005). (b) W. Liu, D. Peng, Exact two-component Hamiltonians revisited. *J. Chem. Phys.* **131**, 031104 (2009). (c) Z. Li, Y. Xiao, W. Liu, On the spin separation of algebraic two-component relativistic Hamiltonians. *J. Chem. Phys.* **137**, 154114 (2012). (d) Z. Li, Y. Xiao, W. Liu, On the spin separation of algebraic two-component relativistic Hamiltonians: Molecular properties. *J. Chem. Phys.* **141**, 054111 (2014).
4. P. Pollak, F. Weigend, Segmented Contracted Error-Consistent Basis Sets of Double- and Triple-ζ Valence Quality for One- and Two-Component Relativistic All-Electron Calculations. *J. Chem. Theory Comput.* **13**, 3696-3705 (2017).
5. (a) F. Neese, F. Wennmohs, A. Hansen, U. Becker, Efficient, approximate and parallel Hartree-Fock and hybrid DFT calculations. A 'chain-of-spheres' algorithm for the Hartree-Fock exchange. *Chem. Phys.* **356**, 98-109 (2009). (b) R. Izsák, F. Neese, An overlap fitted chain of spheres exchange method. *J. Chem. Phys.* **135**, 144105 (2011). (c) R. Izsák, F. Neese, W. Klopper, Robust fitting techniques in the chain of spheres approximation to the Fock exchange: The role of the complementary space. *J. Chem. Phys.* **139**, 094111 (2013). (d) B. Helmich-Paris, B. de Souza, F. Neese, R. Izsák, An improved chain of spheres for exchange algorithm. *J. Chem. Phys.* **155**, 104109 (2021).
6. (a) S. Grimme, S. Ehrlich, L. Goerigk, Effect of the damping function in dispersion corrected density functional theory. *J. Comput. Chem.* **32**, 1456-1465 (2011). (b) S. Grimme, Accurate description of van der Waals complexes by density functional theory including empirical corrections. *J. Comput. Chem.* **25**, 1463-1473

(2004). (c) S. Grimme, Semiempirical GGA-type density functional constructed with a long-range dispersion correction. *J. Comput. Chem*. **27**, 1787-1799 (2006). (d) S. Grimme, J. Antony, S. Ehrlich, H. Krieg, A consistent and accurate ab initio parametrization of density functional dispersion correction (DFT-D) for the 94 elements H-Pu. *J. Chem. Phys*. **132**, 154104 (2010).

7. A. V. Marenich, C. J. Cramer, D. G. Truhlar, Universal solvation model based on solute electron density and on a continuum model of the solvent defined by the bulk dielectric constant and atomic surface tensions. *J. Phys. Chem. B*. **113**, 6378-6396 (2009).

8. (a) V. Barone, M. Cossi, Quantum calculation of molecular energies and energy gradients in solution by a conductor solvent model. *J. Phys. Chem. A* **102**, 1995-2001 (1998). (b) M. Garcia-Ratés, F. Neese. "Effect of the Solute Cavity on the Solvation Energy and its Derivatives within the Framework of the Gaussian Charge Scheme." *J. Comput. Chem.* **41**, 922-939 (2020).

9. X. Wang, C. Wu, Z. Wang, W. Liu, When do tripdoublet states fluoresce? A theoretical study of copper(II) porphyrin. *Front. Chem.* **11**, 1259016 (2023).

10. J. Zhang, W. Zhang, M. Xu, Y. Zhang, X. Fu, H. Fang, Production of formamides from CO and amines induced by porphyrin rhodium(II) metallo-radical. *J. Am. Chem. Soc.* **140**, 6656-6660 (2018).
