# Supplementary material for: Photochemical conversion of CO to C1 and C2 products mediated by porphyrin rhodium(II) metallo-radical complexes
Source: Nat Commun. 2024 Sep 4;15:7724. doi: 10.1038/s41467-024-50253-9 (PMC11374781; doi:10.1038/s41467-024-50253-9)
Supplement: Supplementary file 3 — Description of Additional Supplementary Files [file 41467_2024_50253_MOESM3_ESM.docx]

File Name: Supplementary Data 1
Description: The optimized coordinates of all computationally studied species
