# Supplementary material for: Photochemical conversion of CO to C1 and C2 products mediated by porphyrin rhodium(II) metallo-radical complexes
Source: Nat Commun. 2024 Sep 4;15:7724. doi: 10.1038/s41467-024-50253-9 (PMC11374781; doi:10.1038/s41467-024-50253-9)
Supplement: Supplementary file 4 — Supplementary Data 1 [file 41467_2024_50253_MOESM4_ESM.pdf]

Supplementary Data 1 for

Photochemical conversion of CO to C1 and C2 products mediated by porphyrin rhodium(II) metallo-radical complexes

Hongsen Li[1†], Boao Han[1†], Rongyi Wang[1], Wentao Li[2], Wentao Zhang[2], Xuefeng Fu[2], Huayi Fang[3], Fuqiu Ma[4], Zikuan Wang[5*], Jiajing Zhang[1*]

[1]School of Pharmacy, Binzhou Medical University, Yantai 264003, China.
[2]Beijing National Laboratory for Molecular Sciences, State Key Lab of Rare Earth Materials Chemistry and Applications, College of Chemistry and Molecular Engineering, Peking University, Beijing 100871, China.
[3]School of Materials Science and Engineering, Tianjin Key Lab for Rare Earth Materials and Applications, Nankai University, Tianjin 300350, China.
[4]Yantai Research Institute of Harbin Engineering University, Yantai 264000, China.
[5]Max-Planck-Institut für Kohlenforschung, Kaiser-Wilhelm-Platz 1, Mülheim an der Ruhr 45470, Germany.
† H. Li and B. Han contributed equally to this work.
*Corresponding author. Email: (jiajing_z@bzmc.edu.cn (J.Z.); zwang@kofo.mpg.de (Z.W.))

Unless otherwise specified, all structures correspond to singlet (for even-electron species) or doublet (for odd-electron species) ground states.

(TPP)Rh$CH_2OSi(CH_2CH_3)_3$, **6a**, S_0 state
E = -7337.608316852486 a.u.

Rh	10.05427136852774	9.27857127578562	19.07577441493024
Si	11.93749888711660	7.96682567578606	15.51327708340808
O	10.78708943951889	8.83331701819502	16.35519059540202
N	10.56727447176878	7.61021128074897	20.10015508637459
N	8.09598852953600	8.85459045430300	19.37651076438595
N	9.54530804220432	11.00421548821922	18.15938007396232
N	12.01277173227848	9.78243062713592	18.93253781471801
C	11.83086787560689	7.21596592115019	20.44431180263104
C	11.76553753521204	5.99685736038358	21.20157348739420
H	12.61479067910015	5.48472662527028	21.62455230914183
C	10.46001133022412	5.65317228029645	21.28851854794765
H	10.03011861364360	4.80503375108467	21.79702358158946
C	9.71195719586799	6.66569782662705	20.59678077930331
C	8.32331808801857	6.68717291789579	20.50866998983487
C	7.58403619977215	7.72344270090343	19.94736979169639
C	6.14897814926108	7.76060860133464	19.88724761211607
H	5.49356884898554	6.99144175612149	20.26302185741709
C	5.80791851252746	8.92434484147971	19.28723151575384
H	4.81834684071502	9.30010038718910	19.08164331637038
C	7.03136918664209	9.59815734289430	18.95162413615222
C	7.09835002970319	10.81696289437792	18.28420085069677

C	8.27852691643577	11.45710069680520	17.92201397474570
C	8.33745177737687	12.69737895954137	17.19840959304479
H	7.48062584549820	13.27273611030789	16.88617422815609
C	9.64586217191049	12.97635340754496	16.99984932770651
H	10.07262924322274	13.83260680486009	16.50266252870129
C	10.39977373437380	11.90698394523443	17.59519825545071
C	11.78902961841567	11.83666600346162	17.60363598739945
C	12.52503025814033	10.84342292666302	18.24153368371794
C	13.96051906433305	10.78431438618854	18.26442113424137
H	14.61783622934008	11.48712673910356	17.77822759302567
C	14.30074486665378	9.69578572726358	18.99248018232047
H	15.29033050650838	9.33072276632115	19.21593083069785
C	13.07631864922751	9.06984622864353	19.40879003326377
C	13.01079914130526	7.88236676317335	20.13051190403995
C	7.58175266924674	5.53179848947534	21.07554867347108
C	7.64627483125996	4.28406608132303	20.46026227789168
H	8.23956210069710	4.16856260493483	19.56007370270263
C	6.95789166802233	3.20103853410674	20.98534467849417
H	7.01401714117517	2.23749799787269	20.49171340224425
C	6.19764947269152	3.35062036987872	22.13628097124954
H	5.66011363034551	2.50423840934197	22.54809204350107
C	6.12962401924723	4.58925511134023	22.75795205922461
H	5.54224135334667	4.71340832559389	23.66061416356499
C	6.81610174203806	5.67232058451661	22.23030559641720
H	6.76697751121039	6.63893187369634	22.71905682036541
C	5.81842199334882	11.48458451858665	17.93068744894636
C	5.39917613636052	12.61177630003431	18.63215635049259
H	6.01436847348661	12.99158544423978	19.44021518240793
C	4.20793368183332	13.24272321644472	18.30712382512851
H	3.89298528914853	14.11745751399366	18.86467218200407
C	3.42097234254645	12.75487731882995	17.27389008875826
H	2.49027800905927	13.24814053347557	17.01812244694779
C	3.83155150164953	11.63257940751136	16.56892824694247
H	3.22435315297042	11.24732894859951	15.75767932125553
C	5.02236609312537	11.00123635076543	16.89603762798560
H	5.34559247675698	10.12627621537478	16.34305601235440
C	12.53660476526750	12.90477499056434	16.89314252782671
C	13.30496936672616	13.82825956340883	17.59790731766940
H	13.34751394378907	13.76608149902940	18.67955037702437
C	14.00286879461304	14.82291437673896	16.92999751869141
H	14.59172937739198	15.53745073291321	17.49387534498191
C	13.94401844550043	14.90656788114519	15.54623255090052
H	14.49048695960018	15.68315287682106	15.02352872127635
C	13.18002571819655	13.99195603900533	14.83567481578402
H	13.13013966915379	14.04839233939556	13.75418418518877

C	12.47957141961340	12.99988924484481	15.50468019700690
H	11.88529234711153	12.28317745718061	14.94996712494814
C	14.29102772283694	7.27429603306375	20.57195073660117
C	15.07674260304785	7.90130815363239	21.53601499138432
H	14.73757706196570	8.83537039301170	21.96979115558417
C	16.27584733816458	7.33860768864253	21.94577609751546
H	16.87260505580653	7.83635695132518	22.70165735223072
C	16.70880114790749	6.14150425337237	21.39378329405878
H	17.64690064570068	5.70187750542387	21.71252857787674
C	15.93368223463179	5.50979120397678	20.43187796724679
H	16.26585320021962	4.57630740481805	19.99188566686541
C	14.73291061599873	6.07133625956490	20.02550424262873
H	14.12982576529376	5.57943952165141	19.27106454670255
C	9.92886803980840	8.32531501632086	17.29957665768730
H	10.11223064179954	7.27084113234287	17.53188270008051
H	8.89287095098474	8.48280739268988	16.99088954285494
C	12.99240606841446	6.94486896581807	16.67909046952266
H	13.71148991358197	7.62068438690219	17.15252078411810
H	12.35206489029287	6.58579185004188	17.49257508785253
C	13.71565960995727	5.76481142819382	16.03567541278946
H	13.01074299579388	5.04159282035523	15.61883954075160
H	14.33788085882594	5.23364091487803	16.76170689453793
H	14.37145924375696	6.08241887872659	15.22128305915298
C	11.04676119567162	6.83997653047849	14.30681293728832
H	11.79418002501846	6.28155740947166	13.73164619528147
H	10.50518205967314	6.08753750695101	14.89385141605439
C	10.08946180605600	7.56768064889936	13.36855954641359
H	10.62103642114522	8.28091811042977	12.73282234314400
H	9.33860349278653	8.13202594434914	13.92750740231539
H	9.55907350163665	6.87508767270993	12.70941198800895
C	12.91424394083193	9.29317172765990	14.63267094677105
H	12.20841065479180	9.88021401699632	14.03431232673905
H	13.29681284416036	9.97626295047052	15.39853311352358
C	14.05439634755879	8.78362533505617	13.75718037215389
H	14.55780700541278	9.60195926503503	13.23561330318965
H	13.69848326196642	8.08232328527716	12.99716276440679
H	14.81148516299605	8.26370979818361	14.34957409527770

(TPP)RhCH$_2$OSi(CH$_2$CH$_3$)$_3$, **6a**, S$_1$ state

E = -7337.516042234048 a.u.

Rh	10.04614018066650	9.28735940961262	19.08188156340637
Si	11.96312007552002	7.97470160313502	15.54745611217665
O	10.82916199188382	8.87026715830760	16.38224636069499
N	10.57185289098619	7.58999895936509	20.07296251751959
N	8.07918814193904	8.83986864710090	19.36405563601946

N	9.52570961419059	11.03328573838183	18.18632684670226
N	12.01582679122486	9.81185477164995	18.95480348921963
C	11.83919313111258	7.20214564828733	20.41539917272296
C	11.77848875002318	5.98914886890700	21.18317773447792
H	12.62770394397378	5.48551952992455	21.61543356234011
C	10.47052832822519	5.64365686994620	21.27930625210650
H	10.04564608327631	4.80339006938058	21.80412014347165
C	9.71901334279563	6.64659502456673	20.58086753210190
C	8.32316434190121	6.67011690654640	20.50866814301084
C	7.57084767998630	7.70894592168743	19.94506384170107
C	6.13945995020477	7.74197810498041	19.89316709331693
H	5.48627003561315	6.97313937579916	20.27204624701785
C	5.79145738240937	8.90915201057905	19.29239704810340
H	4.79940196396446	9.28481783332407	19.10239163376867
C	7.01035962918807	9.58243451751381	18.94490275666302
C	7.07391854492447	10.81547117038037	18.28337667899724
C	8.25621391103791	11.47196706960554	17.93571792694487
C	8.31166230594940	12.70251356609141	17.19331919048800
H	7.45481129421568	13.26344943368888	16.85717181948814
C	9.62243502759514	12.99226359241625	17.00270035572893
H	10.04393251227750	13.84726411373457	16.49959244892521
C	10.37891778986139	11.93671622697259	17.61320303150917
C	11.77439560944394	11.87805981619914	17.63082373485694
C	12.52407369195118	10.88509672107261	18.27364954701545
C	13.95589025816730	10.82409656331555	18.27547140293852
H	14.60862475199551	11.53084850410220	17.78979730922042
C	14.30396755760423	9.71469629112446	18.97719780327836
H	15.29527777844664	9.33846063642987	19.16971689236033
C	13.08385894551442	9.08725354356231	19.40156992342085
C	13.02141364536182	7.87628921319426	20.10347957965004
C	7.59011683471422	5.52813698797451	21.09780971800282
C	7.71099720688379	4.25086360155029	20.55146548330243
H	8.33829984262925	4.10611239695925	19.67914030692845
C	7.03152164498052	3.17762416892214	21.10732651442334
H	7.12973499375217	2.19248739585969	20.66566092150206
C	6.22652242242187	3.36392075778595	22.22255614691181
H	5.69821024862150	2.52438762679593	22.65950380911874
C	6.10220468711259	4.63099538332831	22.77615522857415
H	5.48164379580528	4.78366665405947	23.65182269562375
C	6.77550718265853	5.70536902741336	22.21577816969481
H	6.68429352847269	6.69326626563252	22.65276361777536
C	5.79521598334874	11.46145821846540	17.91185573694840
C	5.41755991315075	12.67049142305945	18.49520962270093
H	6.06826288075365	13.12744302222983	19.23221793400828
C	4.22054466132169	13.27893372476035	18.14931502471925

H	3.93836296223221	14.21463164997890	18.61848386115822
C	3.38473406491414	12.69033516946869	17.21035061123445
H	2.44995731570083	13.16674882935856	16.93818741067658
C	3.75277102919439	11.48811014356936	16.62135221812448
H	3.10926658283113	11.02522857693624	15.88181663484085
C	4.94697725259691	10.87715766951493	16.97158638092073
H	5.23697475878409	9.94165547642026	16.50663435998641
C	12.51446591062220	12.93900261033696	16.91340154388384
C	13.31878139198583	13.84395737240816	17.60499188254321
H	13.39177033062111	13.77023539546766	18.68414991571889
C	14.00702992624896	14.83784490704906	16.92637745178357
H	14.62009353579099	15.54034629832200	17.47943612511196
C	13.90777329704800	14.93669336496638	15.54517843873719
H	14.44820577427712	15.71211789997980	15.01456645281334
C	13.11241228879184	14.03824822941011	14.84734778477361
H	13.03385879567830	14.10506775725984	13.76818097752184
C	12.41811548706810	13.04897093199168	15.52657820442712
H	11.80077674505745	12.34315024661813	14.98312831342217
C	14.29952713414657	7.25643715857151	20.51061212326983
C	15.15942390966926	7.90782240409942	21.39554962339221
H	14.87302551087756	8.87365350338772	21.79615749456930
C	16.35915304329451	7.32561110654197	21.77342466327461
H	17.01014473735922	7.84093862862942	22.47049723602365
C	16.72368023704186	6.08534148761869	21.26659244671242
H	17.66332246682269	5.63159914320484	21.55991523298387
C	15.87787903048389	5.42997831735497	20.38213848074457
H	16.15776311536896	4.46466422445679	19.97568732128868
C	14.67483109374900	6.00925712282140	20.00964353759260
H	14.01842124695407	5.50161838049673	19.31251325316479
C	9.92195047961442	8.38008022156185	17.28977037791882
H	10.05124387542984	7.31278725454488	17.50003651111890
H	8.90354833865887	8.59251148470629	16.95516794374689
C	12.90812955920335	6.84848811629600	16.71353152322670
H	13.66461978691334	7.45554592195981	17.22126205571291
H	12.22190021451907	6.52127951733522	17.50270378407977
C	13.55704187330096	5.63197309987301	16.05752896404654
H	12.80966361190933	4.97406429567323	15.60763996121653
H	14.12036838426970	5.03934243409366	16.78409491340322
H	14.25323831249449	5.91906636496148	15.26542894823149
C	11.05969638900134	6.95727479193179	14.25609707286056
H	11.79623103596258	6.38455492716419	13.68096794715918
H	10.45157562997444	6.21189202035270	14.78362688062355
C	10.18460407188939	7.78800933352570	13.32167251260631
H	10.78208624469279	8.49918463887611	12.74487908924863
H	9.44603320151139	8.36720112686135	13.88186349621062

H	9.64213954173263	7.16227451022457	12.60773933807267
C	13.05117463036063	9.27639075597965	14.76495313577365
H	12.40995078674817	9.92720170210669	14.15997345842638
H	13.43665546163278	9.90459148473000	15.57522171470364
C	14.20147643311466	8.73386105547782	13.92171434243439
H	14.78384414185164	9.54116442499496	13.46973827352036
H	13.84212599667262	8.09657327614310	13.10878888060244
H	14.89029932326951	8.13548114870188	14.52359863275866

(TPP)RhCH$_2$OSi(CH$_2$CH$_3$)$_3$, **6a**, T$_1$ state
E = -7337.542868432573 a.u.

Rh	10.05315904606701	9.29424449732691	19.07326203688125
Si	11.94225769884474	7.94390380786434	15.55357023092837
O	10.79137998790183	8.84630965389737	16.35932710846608
N	10.57373780590811	7.58521070955795	20.06444377522195
N	8.07976208118598	8.85615063820111	19.36273065421341
N	9.54056711893768	11.04495207411589	18.16596420874379
N	12.02731066056859	9.80534199170937	18.92753669838448
C	11.83358695465002	7.21267996327688	20.44763683321417
C	11.76541276612988	5.98079725428007	21.18952438719795
H	12.60712473335650	5.48211094152415	21.64216615570923
C	10.46734009732081	5.60709667040006	21.22026062070326
H	10.03872562796637	4.74452590194944	21.70477335913201
C	9.72039994620651	6.61855905990581	20.51963165550531
C	8.33561809791514	6.63402521942958	20.41674909126456
C	7.56360268764829	7.70456627926258	19.89098783003572
C	6.15348967850948	7.74340127379443	19.85021363381069
H	5.49262789697496	6.96407001620145	20.19264463634628
C	5.80642717853446	8.95864577129693	19.30118351500812
H	4.81477685238944	9.34587779085788	19.13338812206987
C	7.01250051495369	9.62124275962837	18.98888254026891
C	7.09890817539114	10.89425931495139	18.35556029837065
C	8.27346592445729	11.52380832375229	17.97565015775400
C	8.33342135684474	12.76263738764436	17.24278196921058
H	7.47904138199788	13.35483384235466	16.95695256705242
C	9.63670843669576	13.00856164188193	16.98831720516555
H	10.06103924276581	13.85257653740185	16.46877350320548
C	10.39265302703125	11.92584473174772	17.56437113807930
C	11.77778238993230	11.83870825473620	17.54421307600444
C	12.54641142400220	10.84621167833483	18.21098434846468
C	13.95650216423033	10.79758670121373	18.23935948201541
H	14.62057381921836	11.48537730378379	17.74202159963867
C	14.30001408976859	9.71228074081844	19.01686128406346
H	15.29045228172205	9.35810656925897	19.25210927837764
C	13.09134962966036	9.11340969216736	19.43113539699803

C	13.00502186557178	7.90492584895254	20.17780944231288
C	7.60086042352156	5.45438422568789	20.92539839100616
C	7.74157996551930	4.21744566710482	20.29899343284972
H	8.38556304947576	4.13364783358340	19.43074768417608
C	7.06024762519155	3.10595293367351	20.77076511777111
H	7.17418017393595	2.15257174275009	20.26755541466552
C	6.23572320101963	3.21390117326923	21.88101143304860
H	5.70580084401987	2.34451500241308	22.25284054030120
C	6.09318703822623	4.44100135560351	22.51427099963680
H	5.45655628133980	4.53194923541829	23.38697263840870
C	6.76526941321575	5.55471033701742	22.03668923282207
H	6.65496864544934	6.51171543549063	22.53356617137033
C	5.82356330982554	11.58487355402620	18.05573269167441
C	5.48014469169637	12.75293159360202	18.73236687345649
H	6.15088317106522	13.14423774880157	19.48901555921239
C	4.29117866024624	13.40796107089991	18.44852844966517
H	4.03476899352427	14.31304118329990	18.98729365837848
C	3.43391562870264	12.90727410281194	17.47985536867532
H	2.50650948526718	13.42125110931154	17.25477496271582
C	3.76946179222021	11.74481856346280	16.79944741866597
H	3.10773877846028	11.35044450656158	16.03668141675818
C	4.95285953439393	11.08439119348436	17.08942295829907
H	5.21578248006447	10.17758101098397	16.55679464841950
C	12.51818201605161	12.87207940346975	16.78744092389075
C	13.35453617691761	13.77740357930719	17.43906475416120
H	13.45701627257954	13.72423514564537	18.51681023867068
C	14.03756206704758	14.74672466769441	16.72220044201236
H	14.67446197847023	15.45146384028520	17.24461676491244
C	13.90530054463512	14.81772187197623	15.34216492300497
H	14.44368851501380	15.57326251977153	14.78160184639917
C	13.07904848710902	13.91810395758603	14.68468592621649
H	12.97236864407573	13.96387675787434	13.60685750627229
C	12.38636912123246	12.95517646956261	15.40239188833811
H	11.74236691577224	12.25023421540447	14.88965851112929
C	14.28047511548221	7.32985765024512	20.65613610372211
C	15.05558127295130	8.01363446447820	21.59185994462726
H	14.70853495261259	8.96727279505749	21.97305425581310
C	16.25074414795467	7.47739074113946	22.04292907569072
H	16.83554823394371	8.01448321521526	22.78097057357194
C	16.69793653585090	6.25786383908085	21.55292705854655
H	17.63589683771504	5.84149197910996	21.90189328833708
C	15.93935612708891	5.57575783084154	20.61300187434832
H	16.28479860907595	4.62676004396766	20.21893414201719
C	14.73711560629801	6.10574921172081	20.17018541817017
H	14.14686764685086	5.57602839651569	19.43138634653567

C	9.90694859210225	8.36408437817171	17.29196035380604
H	10.04599371872400	7.30045658139360	17.51490595041852
H	8.88088283598606	8.56584331524336	16.97603054315589
C	12.97023950077450	6.94090154553514	16.75872001404870
H	13.67465030088091	7.62673445601676	17.24008615859163
H	12.30993372432856	6.59073105773103	17.56043694165240
C	13.71207467803658	5.75409519393246	16.14942653701887
H	13.02011447611471	5.02255741055800	15.72515880929212
H	14.31796668321923	5.23493069048068	16.89768210041942
H	14.38679024414725	6.06295610854962	15.34728418513057
C	11.04614775967708	6.79482856227215	14.37097011992938
H	11.78982465770207	6.20542560229626	13.82244162027929
H	10.48481079138579	6.07056925963527	14.97482139970644
C	10.11246433899152	7.50837409334484	13.39854698949074
H	10.66447875859115	8.18834221971690	12.74397168966536
H	9.36785813050802	8.10671961186200	13.92991939423088
H	9.57422286903671	6.80417129789596	12.75829803795406
C	12.93858878899446	9.23525920833291	14.64287774420048
H	12.23899567896659	9.81853103436594	14.03337852670447
H	13.33338279489731	9.92903114569153	15.39295935752749
C	14.06779347719478	8.69022899199638	13.77432957403742
H	14.57719037547962	9.48935025972670	13.22924715131828
H	13.69993678445455	7.97369626581315	13.03433909480171
H	14.82271838743456	8.17736569074812	14.37545499843247

(TPP)Rh(II)
E = -6695.428819546166 a.u.

Rh	6.00557429752966	8.49674912927521	14.97025642356976
N	6.49979258512803	8.35561228749070	13.00924595690548
N	7.88768495471508	7.94549179476323	15.48466708309534
N	5.51314721066896	8.64534448540056	16.93105149977167
N	4.12048685066670	9.03741450788349	14.45632747675775
C	5.68702819625948	8.62638422719914	11.94403965422947
C	6.43949108834828	8.51098262436982	10.72570478144409
H	6.04885961665580	8.69514068314469	9.73774529939535
C	7.69827128129390	8.15208192856671	11.06901241194808
H	8.54064981231363	7.98473782645518	10.41710577012167
C	7.72987437365452	8.04376257109769	12.50123596943530
C	8.85220637412238	7.67607239682681	13.23838206835098
C	8.90038956611925	7.61448999145753	14.62830965359679
C	10.04227996640473	7.17757323317301	15.38260120764505
H	10.97188730324107	6.83667459170853	14.95568854062466
C	9.71031829245330	7.26437529747649	16.69156014918816
H	10.31491534442877	7.00866454605387	17.54685615399217
C	8.36248749444933	7.75816992008138	16.75286316117127

C	7.66477991600710	8.00012881978580	17.93313639914135
C	6.34466798478489	8.43843524481452	17.99594611900754
C	5.64905087017396	8.74803434840581	19.21424901453716
H	6.07817129021050	8.69576477220348	20.20201374513650
C	4.39495478608251	9.12307587983807	18.87105721654622
H	3.59574384934253	9.43776110117305	19.52279545272090
C	4.30841019513734	9.04423566026718	17.43923106032023
C	3.16279763969922	9.33233755048623	16.70231100672887
C	3.08768514983986	9.29991272404623	15.31241946197458
C	1.88860772738312	9.53779150146832	14.55770544814497
H	0.91991690681131	9.74384357834862	14.98393217903525
C	2.21621711883532	9.43453313580565	13.24881554913540
H	1.56829533673124	9.54027695215696	12.39350102162276
C	3.61945537925179	9.13289596145063	13.18808372624046
C	4.33865099058803	8.96673857952504	12.00763045196760
C	10.08420534195523	7.32083667585398	12.48841866789844
C	11.20717423523812	8.14299879242098	12.53936354946797
H	11.17310869280704	9.05134268468787	13.13059919232902
C	12.35643019639623	7.81310450698478	11.83693185165592
H	13.21990451437294	8.46704333295564	11.88248870529958
C	12.39996111403276	6.65330323489093	11.07647732756282
H	13.29863414773832	6.39441239221451	10.52846010504483
C	11.28675857291343	5.82685478293217	11.02139089008371
H	11.31326516098885	4.91659792180743	10.43319140754611
C	10.13671031314661	6.15971559519884	11.72101742895859
H	9.26815063003427	5.51189631146655	11.67949389948597
C	8.37505750297354	7.76773155757507	19.21690955382226
C	9.43348818161396	8.58532944974288	19.60542710018987
H	9.73516725586996	9.40452113290863	18.96222340925647
C	10.09320321014946	8.36387304956876	20.80499818842286
H	10.91142084254048	9.01299030265681	21.09557437475219
C	9.70565187128348	7.31897473922102	21.63158188567079
H	10.22226514588295	7.14498578687746	22.56847229044035
C	8.65292172931804	6.49847130964298	21.25238649199127
H	8.34574149111291	5.67724668152428	21.88995500892256
C	7.99171979187100	6.72292989027836	20.05453902403168
H	7.17153456233575	6.07896695428962	19.75724903472634
C	1.93303345742247	9.69691255387303	17.45128810317020
C	1.41664996760742	10.98856546238961	17.38003425456895
H	1.92588366087921	11.73016711866815	16.77472873704299
C	0.26943981910284	11.33013337181985	18.08001728614417
H	-0.11609028852641	12.34150292713768	18.01873042195705
C	-0.38006635905682	10.38296079004556	18.85869312541891
H	-1.27757888339127	10.64933172729554	19.40501168623232
C	0.12564143515784	9.09303933568890	18.93428946356245

H	-0.37787101257512	8.34533286795394	19.53652535911769
C	1.27493120521456	8.75344871817790	18.23672031355797
H	1.66746653431689	7.74427644552280	18.29313812520928
C	3.61629124686496	9.16899906565600	10.72559513405742
C	3.17512526588337	10.43643372894859	10.35290106325526
H	3.36906525996329	11.27879669643643	11.00752011373936
C	2.50150035384526	10.62604813359244	9.15581009763667
H	2.16961307490212	11.61995037346568	8.87779449698375
C	2.25657187211179	9.54938215161255	8.31549926634395
H	1.72878496164834	9.69695923954520	7.38030047414860
C	2.69123488680680	8.28272542389825	8.67878567083592
H	2.50084295987097	7.43500940956826	8.03041695791914
C	3.36767058147663	8.09477212216433	9.87456545096974
H	3.70400843656773	7.10408373863968	10.15951406706489

$^{\bullet}CH_2OSi(CH_2CH_3)_3$

E = -642.086904837511 a.u.

Si	1.33035781718488	5.57406624626737	14.63320933619883
O	2.70225607545142	5.15753122509156	15.51487492970930
C	3.69820793978063	6.01197655487892	15.81581022952848
H	4.48735247450626	5.61455656769934	16.44031187667394
H	3.53496874894742	7.08103007995351	15.72436757409015
C	1.89869690535206	6.27144730256949	12.98746290547950
H	2.32370570482337	7.26688787226354	13.15900008801090
H	2.73543886401144	5.64578655116983	12.65541499607233
C	0.83081503373159	6.33182856590335	11.89708832917042
H	1.24188133729192	6.70683691142188	10.95614861413441
H	0.00245103983272	6.98779395722022	12.17336145169187
H	0.40749106684600	5.34420979942498	11.69663254057317
C	0.35534571787453	6.79977112958766	15.66203914510980
H	0.18589687445531	6.32512587418477	16.63588684349233
H	0.99745390033403	7.66574437650737	15.86055874521194
C	-0.97283597151887	7.25972113776018	15.06533861843755
H	-0.82542448155469	7.81701347153060	14.13754056031641
H	-1.51464183409173	7.91405661871437	15.75323830624177
H	-1.62716844292616	6.41423082658342	14.83717219566322
C	0.40718860757988	3.96568315594486	14.42233609211651
H	0.13089396831310	3.60792548328007	15.42080200662259
H	-0.53770504938702	4.18333585642596	13.91100594942926
C	1.18423310296195	2.89020549326866	13.66758674141050
H	1.43473585688561	3.21426479192125	12.65391888641218
H	0.61079771405047	1.96349926991959	13.58204956543455
H	2.12283344926387	2.65335703050711	14.17385568276787

CO

E = -113.300015514219 a.u.

C	0.00000000000000	0.00000000000000	0.00346731169750
O	0.00000000000000	0.00000000000000	1.12653268830250

•CO…$CH_2OSi(CH_2CH_3)_3$ transition state

E = -755.388373470319 a.u.

Si	11.10276483310598	7.11989300483217	15.98645198934084
O	9.92843820666873	8.19761480527219	16.54663714059285
C	8.75489530334495	8.51425270062045	15.99873998494379
H	8.36998522804894	7.90074538832692	15.18863782317843
H	8.05025280436937	8.99776518390487	16.66657943026944
C	11.12908631898903	5.77184797355965	17.28440581262599
H	11.29393061497568	6.25557447622147	18.25401441755859
H	10.11155305228926	5.36430376468466	17.32656143659729
C	12.13557723585368	4.64289423899769	17.07911252818571
H	12.00321021284694	4.15232786851462	16.11124352837616
H	12.03334490146895	3.87389487011430	17.84914333377503
H	13.16412920281766	5.00741965873977	17.12125348413987
C	10.62682421223771	6.41830857369792	14.31736609875819
H	11.33246874188042	5.59910426769641	14.12971914998528
H	9.64700056847395	5.93460197391740	14.40726251345179
C	10.64171018753904	7.39178589999442	13.14212950888358
H	11.61296439213454	7.88173244774035	13.03947521865196
H	9.89645952679512	8.18091401422810	13.26035965084175
H	10.42989497640613	6.88250650071050	12.19860656812551
C	12.67328381645303	8.12911198560060	15.89050020596342
H	12.42658948399345	9.03635247744581	15.32774894642123
H	12.91917766371571	8.46713425335104	16.90343270834882
C	13.87273851343670	7.42917463270576	15.25507923493324
H	14.73124094911507	8.10154368132611	15.18206652372658
H	13.64633334892854	7.08074069589761	14.24402084091037
H	14.19036470256287	6.55888967923349	15.83302405318648
C	9.10228048722607	10.43125654208121	14.75308767674800
O	10.18493950432188	10.76153011058409	14.88504728147889

•$COCH_2OSi(CH_2CH_3)_3$

E = -755.413077266164 a.u.

Si	1.36190393059332	5.49804859352535	14.57607810888850
O	4.98054128562311	4.60694550045247	13.73106839135007
O	2.72612009156894	5.03610957575510	15.43546366782053
C	4.97817718282024	5.42186273594818	14.57812928272173
C	3.88218547297353	5.79404852271273	15.55054133672995
H	4.32281779610344	5.66398892181780	16.54623636985355
H	3.72441509584413	6.87417385901988	15.42229912503297
C	1.90091980139261	6.17119232228346	12.90908321069572

H	2.42096744097630	7.12480153692293	13.05953547632287
H	2.65480138888351	5.47913781323577	12.51786613317456
C	0.78093437873342	6.34225547255095	11.88381399166168
H	1.17258631423224	6.67305961282752	10.91846110252097
H	0.04249517544959	7.07980455492245	12.20451559260035
H	0.24807632197561	5.40301158198292	11.71422842469823
C	0.46192169869332	6.76401764793343	15.62809682499739
H	0.33199136876817	6.30278219643518	16.61458721426433
H	1.13401860846893	7.61644412705360	15.78572464794445
C	-0.88497181848956	7.25323611542042	15.10084556638881
H	-0.77497601201411	7.80573659026026	14.16536421410763
H	-1.37598096619520	7.91989152288413	15.81462571293970
H	-1.56793397514330	6.42153453572756	14.90916790381250
C	0.37368814438947	3.92541645340429	14.39313803478748
H	0.14278026945302	3.56029328318370	15.40057331890782
H	-0.59131933493950	4.17461261775718	13.93710851620446
C	1.08217046721155	2.84245241507724	13.58310410926673
H	1.26818581504342	3.16939898122295	12.55669559473463
H	0.49085965106996	1.92452619975427	13.53218861367687
H	2.05011596651381	2.59075467992814	14.02245057389518

[(TPP)Rh(CO)]•

E = -6808.756839207006 a.u.

Rh	5.86931418410418	8.36789685383846	14.84194007686774
O	5.80376945040084	5.42434566025032	14.25845241067046
N	6.41195583120571	8.40405596078585	12.88380230305376
N	7.78426672939083	7.90823722487473	15.35995941978020
N	5.40785663608589	8.61622483846523	16.81460337060469
N	4.03279929716304	9.10924271045818	14.33495839527190
C	5.61526594143502	8.73832985408206	11.82589118535248
C	6.37983638544289	8.65865055423392	10.61236295423858
H	6.00687768802537	8.90205759311810	9.63038822853578
C	7.62496041371349	8.25151326788674	10.95230862583323
H	8.47207894594292	8.09615777046650	10.30359782947035
C	7.63804678761633	8.07991247501431	12.37839789639092
C	8.74516336818205	7.65825819471668	13.11110899451987
C	8.79140329379257	7.57426324676884	14.50050276534070
C	9.92732793202955	7.11895292316982	15.25211644371551
H	10.85641218376300	6.77879192285024	14.82362482518069
C	9.59376384794064	7.19528546929383	16.56172728883678
H	10.19658880676615	6.92999183727300	17.41533520885500
C	8.24972758342168	7.69790010276129	16.62694033107557
C	7.54975447976424	7.93072832073254	17.80862332744189
C	6.23687841958973	8.39233379171533	17.87485390680653

C	5.54948209494708	8.71567161637883	19.09467046100351
H	5.98119095078256	8.66080132540541	20.08122648014021
C	4.30196589498525	9.11372384956275	18.75289787933611
H	3.51135469933521	9.44923514359221	19.40480840593399
C	4.21250724692880	9.03717461350727	17.32049099794795
C	3.07343616361388	9.35867091896235	16.58491921367405
C	3.00542210130168	9.37350650609239	15.19331317719092
C	1.82174126391101	9.68181497710084	14.43907353568561
H	0.85838136057005	9.91197048395853	14.86505239213665
C	2.15677950225552	9.61491041047847	13.12936896221817
H	1.52136736975569	9.77962760025449	12.27397592803939
C	3.54871847901088	9.26392512903704	13.06863961421694
C	4.27423087582094	9.10971741540636	11.88922675644035
C	9.96600003427196	7.28182306177239	12.35374555471576
C	11.11538022677663	8.06553554674381	12.42168520771346
H	11.11149783696923	8.96102810609372	13.03319964931583
C	12.25199537170969	7.71436010909463	11.70923371011833
H	13.13671986934223	8.33813313384701	11.76770538652612
C	12.25582159459922	6.57201496304842	10.92158643221122
H	13.14464140112116	6.29663763888360	10.36560257406973
C	11.11588515218901	5.78421644798196	10.84928373529415
H	11.11176567307091	4.88776445621857	10.23970354582549
C	9.97832372629057	6.13813315605140	11.55877556515476
H	9.08835332928693	5.52098920115184	11.50487801828452
C	8.25378176156841	7.67550147184102	19.09142588272560
C	9.32360731418444	8.47417529106932	19.48860157366149
H	9.63819661623584	9.29414313769600	18.85264264592213
C	9.97734308724315	8.23302147444074	20.68748806349258
H	10.80458956460815	8.86718254410883	20.98537144121141
C	9.57221260605854	7.18724534943813	21.50449218062807
H	10.08425638111060	6.99759370049911	22.44086167477272
C	8.50774117643082	6.38607304491426	21.11690725760523
H	8.18693392472379	5.56460387059150	21.74737745457950
C	7.85211830125179	6.63028935356049	19.91982903197814
H	7.02268947405869	6.00167649831712	19.61547347046049
C	1.84913391937245	9.72649894136377	17.34107287643003
C	1.35293943097438	11.02752016905002	17.30056835421060
H	1.87361316775854	11.77503547873769	16.71250727253876
C	0.21230961516483	11.37043917874187	18.01042437062254
H	-0.15747002870055	12.38884437819940	17.97379881471553
C	-0.45059544386460	10.41559850538190	18.76822527414800
H	-1.34299650055898	10.68320350011651	19.32226054039930
C	0.03524564118733	9.11669540004776	18.81357837933573
H	-0.47885790085230	8.36351233629572	19.39982115573767
C	1.17822932358540	8.77551285657773	18.10662311194504

H	1.55623464546781	7.75978055984625	18.13953376166993
C	3.57125829322444	9.37045309423719	10.60708154765081
C	3.16208501544501	10.65895493809214	10.27141383453861
H	3.36741442371086	11.47507157279517	10.95515993804786
C	2.50684649728893	10.90201967856404	9.07387051960575
H	2.19989897564583	11.91140527440928	8.82451493842132
C	2.24872415143682	9.85867576245123	8.19626464635598
H	1.73528443846304	10.04809152262261	7.26061028241452
C	2.65190423993876	8.57164312313960	8.52230634676970
H	2.45104450786923	7.75004214573518	7.84419651222085
C	3.31035610955263	8.32999008513814	9.71839771616529
H	3.62238107848907	7.32368474008235	9.97482896776594
C	5.46427011730422	6.43270502851706	14.67110476021562

(TPP)Rh(CO)($CH_2OSi(CH_2CH_3)_3$), **6a-CO**, S_0 state

E = -7450.926979228627 a.u.

Rh	10.06002078029037	9.39399247156678	19.24927215996957
Si	11.96404404050808	8.01225119939734	15.60892703578621
O	10.93084747789780	8.93768633759437	16.51293583769279
N	10.57941206198141	7.65107106952967	20.17807481233684
N	8.09004157922359	8.88949407069579	19.41307265348711
N	9.54895084039301	11.07676861641852	18.22328349055781
N	12.03512386227313	9.85294088967877	19.02116190215867
C	11.84265422785861	7.25573980017858	20.50101246343504
C	11.77850785437779	6.02501867705356	21.24147938346281
H	12.62956386369789	5.49885459280737	21.64288741784416
C	10.47155229443703	5.68735101744938	21.33813326725646
H	10.04359413546909	4.83128599742670	21.83463200158265
C	9.72086189783923	6.71179137956016	20.66456150825273
C	8.33056176077681	6.73352830566670	20.56859345081434
C	7.58985568180121	7.75569692683677	19.97874394155784
C	6.15617903906404	7.76744764929733	19.87221960873708
H	5.50322059225533	6.98606514348184	20.22641324222402
C	5.81315729901618	8.92187137411424	19.25454234960539
H	4.82386630590428	9.27547679303935	19.01214880691039
C	7.03423778160071	9.61875744108834	18.95526766132375
C	7.10277792505816	10.84357221893075	18.29497361841204
C	8.28355708898832	11.50080165560074	17.95538721448649
C	8.34372840246365	12.73399311031257	17.21830269587635
H	7.48737708153467	13.28925541684376	16.87056865876452
C	9.65321245351158	13.03201415226815	17.05243851163560
H	10.07948762277857	13.88640708500421	16.55180233864816
C	10.40821736225680	11.97964395265672	17.67770284410911
C	11.79937447306273	11.91908668812949	17.71057886810779
C	12.53632672693339	10.91332722783526	18.33089560573584

C	13.97218673704099	10.83251423613132	18.31735476696709
H	14.62670084642797	11.52947749271494	17.81913276958606
C	14.31380999949070	9.72657258760832	19.01804614348464
H	15.30238272046304	9.33834294759023	19.20351872367578
C	13.08983374977620	9.11473896574664	19.46092425656533
C	13.02311162121578	7.92087454832625	20.17667142415847
C	7.58542599343280	5.58043756766659	21.13501736062483
C	7.66866809762959	4.32518868407784	20.53705320992902
H	8.28051790164317	4.20147229009441	19.65056892897204
C	6.97412216737960	3.24573089031196	21.06091360592043
H	7.04516634418038	2.27653872076036	20.58059314780519
C	6.18798102236590	3.40615552131781	22.19278190771234
H	5.64543424413627	2.56245221793023	22.60333194505876
C	6.09995566182299	4.65230006592076	22.79640687561987
H	5.49158517696440	4.78519975356890	23.68371486302651
C	6.79304532102113	5.73177654253673	22.27025101568952
H	6.72774055344880	6.70434435421787	22.74497728274949
C	5.82356425341155	11.49266274914564	17.90723360501520
C	5.38337575789797	12.63173630712796	18.57656912479665
H	5.98172179264787	13.03396129424683	19.38651577666061
C	4.19262179005909	13.24542054897583	18.21844737073797
H	3.86145165828205	14.12928567493142	18.75182942271355
C	3.42677176050678	12.72849347858349	17.18338738956439
H	2.49646329601159	13.20828876491607	16.90174387140640
C	3.85836569977938	11.59478529311679	16.50963498697059
H	3.26826460600237	11.18750487306382	15.69646293323894
C	5.04863933564857	10.98079846466261	16.86969131045527
H	5.38896120332601	10.09776947305213	16.34019054339413
C	12.55281708816019	12.99267813492129	17.01510658038463
C	13.31670874014912	13.90831393112733	17.73480106686185
H	13.34785840751988	13.83766016060740	18.81638160718760
C	14.02532274774422	14.90527718963325	17.08165940792781
H	14.61060878037666	15.61368633755279	17.65692868498089
C	13.98220443821793	14.99844518614608	15.69794302196535
H	14.53714993264891	15.77665662657288	15.18666413414546
C	13.22433785650369	14.09036376029066	14.97235944306195
H	13.18862151076069	14.15315009310572	13.89063819194534
C	12.51336125880080	13.09584715898861	15.62637203706072
H	11.92594498096787	12.38247144856249	15.05985027245635
C	14.30563873665112	7.29451754012495	20.58389008563092
C	15.12544199446705	7.90706460506662	21.52879899281981
H	14.81047496747538	8.84374368371502	21.97480810946150
C	16.32742152432807	7.32680200628056	21.90393039136948
H	16.95074301061634	7.81342082827540	22.64538494219032
C	16.72940768009328	6.12656402909362	21.33584876807739

H	17.66981629123361	5.67345603713905	21.62751886637784
C	15.92066212426779	5.50952097777024	20.39241179914227
H	16.22898770364130	4.57424727901100	19.93923069789713
C	14.71716061267880	6.08841377928166	20.02060466587573
H	14.08944663318811	5.60932703674953	19.27836062806714
C	10.02316860202133	8.42485698093650	17.44361109516423
H	10.20019032223343	7.36175282675858	17.64976547578244
H	9.00491177590889	8.54983023216582	17.06092426887660
C	13.01969587885022	6.92301936089732	16.71733385670258
H	13.78644473279345	7.55774931197437	17.17341750382549
H	12.38814302815831	6.58869512251346	17.54839916742535
C	13.66277782737830	5.71444761461825	16.04350678522706
H	12.90979524973114	5.03695771996745	15.63370985123017
H	14.27131752429832	5.13882415994082	16.74743265397197
H	14.31615948406188	6.00736107883961	15.21801101184556
C	10.96493805878708	6.93637025140655	14.43828139726048
H	11.65691151481108	6.36160511499582	13.81194299947236
H	10.42680952529383	6.19439209862432	15.04122737160484
C	9.98635753024728	7.72036838708802	13.56970594760264
H	10.50960167359669	8.42596096770093	12.91837372648826
H	9.29282175218274	8.30245477891109	14.18222119840904
H	9.39095582965921	7.06437238401641	12.92860164454469
C	12.97967393408710	9.27720412145728	14.67634161525763
H	12.27952702485599	9.92925524368275	14.14184260813263
H	13.46817548184467	9.91398867558096	15.42233797808025
C	14.01112494495143	8.70443856802371	13.71012345961067
H	14.54101145357113	9.49363746476726	13.16978366750528
H	13.54738038157939	8.05360919094202	12.96349146384062
H	14.76406698994599	8.10999399073482	14.23376701379188
C	9.97331468889649	10.40453818301063	21.04434273719246
O	9.84275592449039	10.98915783161621	22.00000860298479

(TPP)Rh(CO)($CH_2OSi(CH_2CH_3)_3$), **6a-CO**, S_1 state
E = -7450.854029838219 a.u.

Rh	10.04093949843845	9.42626495455344	19.16877921373395
Si	11.86685553491006	7.66936170698815	15.61764052317359
O	10.60605112148584	8.68024703071248	16.19090632898581
N	10.60966625731069	7.63814791757874	19.98045295920893
N	8.07510493405994	8.91972619631430	19.41903266347479
N	9.48165381810257	11.12094067641800	18.18567556587145
N	12.00695712278093	9.87361004682468	18.82068440578490
C	11.89323926779769	7.27814149379282	20.31577783306626
C	11.84745271414834	6.02098256736331	21.01821581578000
H	12.71042740909318	5.50552676874013	21.40841838815353
C	10.55408308832328	5.64307545124233	21.09365791277174

H	10.14912198417616	4.75993326197305	21.56164244008768
C	9.76308638940607	6.66999060567989	20.46378392458029
C	8.38494439291089	6.68533805215626	20.44327535974816
C	7.59909037659059	7.75729420664078	19.96596322780476
C	6.18514085067559	7.80053576179275	19.98233271153030
H	5.54524357147973	7.01224421507895	20.34420834088426
C	5.80912822539614	9.01421428908495	19.45984233338160
H	4.80896825239516	9.39350917287853	19.32719067909011
C	6.99331342198883	9.69821301705840	19.09769625877690
C	7.04183272595838	10.95768182959140	18.46097524510708
C	8.19667331824901	11.57538947136571	18.02977187302881
C	8.23276402658202	12.79535947097685	17.26195040633218
H	7.36252244787838	13.36678481108441	16.98054237097554
C	9.52428602660952	13.05047258428727	16.96517328254669
H	9.92433921100631	13.87855792464977	16.40159241028985
C	10.32053127144540	12.00210739734739	17.55225455773120
C	11.69546833968303	11.94770333461472	17.50518189302352
C	12.48215820294891	10.96852462004199	18.14746558953827
C	13.89537467245259	10.95986826732198	18.18725329269537
H	14.53255703422655	11.68859022684891	17.71290036141892
C	14.27413250629118	9.85414783320710	18.91124258386992
H	15.27535764520532	9.51985786906235	19.13022239155617
C	13.09055137624809	9.18709480919601	19.30500399979730
C	13.04677415969799	7.97691251396811	20.03250088306287
C	7.66900335305348	5.51503728397516	21.00820208652754
C	7.70635580508410	4.27996437156736	20.36467375078158
H	8.25619061664967	4.18604001918894	19.43468659856634
C	7.05080050019944	3.18052092066707	20.90047947261703
H	7.08992387675848	2.22710528468734	20.38571019284543
C	6.34431575079289	3.30180753234714	22.08831865854676
H	5.83225017954036	2.44372184867834	22.50842362873026
C	6.29732756890552	4.52907234906619	22.73630878974439
H	5.75227844826679	4.63149283248184	23.66792610619342
C	6.95306480882753	5.62610762016524	22.19912450336090
H	6.92107216488752	6.58263641561553	22.70863491387201
C	5.75103490833992	11.64626249851754	18.21117071364989
C	5.42420145324722	12.80502811268485	18.91198431940904
H	6.12138538652912	13.19058044226199	19.64757158170879
C	4.22418985995692	13.46093397628781	18.67793289910421
H	3.98576054023370	14.36082435730728	19.23364129383380
C	3.33116648094170	12.96459720224270	17.73939706358306
H	2.39325126191379	13.47594499869256	17.55536162252763
C	3.64592281670786	11.80914853158987	17.03668897544219
H	2.95596851218721	11.41764608888520	16.29764423551502
C	4.84574919074447	11.15517122574032	17.27236314257183

H	5.09278484179683	10.25550458136640	16.71983628595857
C	12.41187089434756	12.99937026774756	16.74075354511977
C	13.10249987901474	14.01729230698042	17.39433078470323
H	13.11036014791410	14.03920316197063	18.47841456707659
C	13.76760930993532	14.99718393060700	16.67227292344872
H	14.29509585969865	15.78660150533213	17.19579342471235
C	13.75330538896765	14.97148412405876	15.28421011143104
H	14.27237738204153	15.73771738561870	14.71985635373752
C	13.07005586473186	13.96053797425585	14.62348010032521
H	13.05493270184000	13.93214922900574	13.53974134797345
C	12.40563776662371	12.98116887975362	15.34787662951002
H	11.87146856044392	12.18958275245838	14.83377217646244
C	14.34394435520838	7.42190881411867	20.49018649845664
C	15.08618181984124	8.08112723882819	21.46896193003568
H	14.69876867272110	8.99979779283831	21.89517808835839
C	16.30114349057008	7.57183531732687	21.90065714287970
H	16.86083179725000	8.09412588923615	22.66840875036618
C	16.79734340742509	6.39467493803009	21.35644431806111
H	17.74757989573610	5.99637188241311	21.69321170322498
C	16.06959733701389	5.73230709388273	20.37849368351803
H	16.45118224754008	4.81525002420135	19.94369825133322
C	14.85353911668023	6.24376471514586	19.94852028135321
H	14.28938170565777	5.72847313663349	19.17997831838839
C	9.68993558201608	8.39137100895851	17.08820358186892
H	9.66589285909130	7.37783540160826	17.48684456349230
H	8.74512794586685	8.90488771818028	16.93912369083823
C	12.08146000746711	6.26509167769878	16.82587748684072
H	12.19284954244401	6.68658081323737	17.82959130270028
H	11.15639452143945	5.67728748182487	16.84130636736982
C	13.26706853618362	5.35429135081450	16.50877961997901
H	13.20805468790102	4.94178049683041	15.49812668307869
H	13.31051474054462	4.51074327151490	17.20236022039354
H	14.21410272549537	5.89229068191721	16.58979630549740
C	11.23965266039204	7.06651453054160	13.96351191968737
H	11.99358511222898	6.38431968136739	13.55297896344256
H	10.34935999153041	6.45403387439825	14.14676655333855
C	10.92855345519357	8.17978931710604	12.96648753716025
H	11.81515295491161	8.78075098699974	12.74889237677687
H	10.16434255623202	8.85725516424079	13.35480639333851
H	10.56363831158854	7.77837412710339	12.01784304605742
C	13.32530708745395	8.80742028705936	15.43668964927948
H	12.93846092594280	9.76000384776442	15.05756723151866
H	13.71703769998362	9.02839352835895	16.43374591570265
C	14.43664901236362	8.29269399317347	14.52418393001033
H	15.24472948980791	9.02376026532217	14.44331686503017

H	14.07224767746279	8.09462627189142	13.51291232000266
H	14.87463314781019	7.36501877939409	14.89896106977901
C	10.10696583684921	10.26586490435828	20.81299419813202
O	10.13013578906787	10.77807838743016	21.81977782623865

(TPP)Rh(CO)($CH_2OSi(CH_2CH_3)_3$), **6a-CO**, T_1 state
E = -7450.870042062329 a.u.

Rh	10.05235512507492	9.40594034891611	19.25571408003221
Si	11.96624764386242	8.01217091180260	15.62689922259907
O	10.89069917877953	8.95284460091998	16.47152203744441
N	10.57850181809156	7.62504423820662	20.13343897960818
N	8.07155576866981	8.89203342668084	19.40404725074744
N	9.54058044026190	11.09606765093991	18.22523175969132
N	12.03856574529575	9.85727617643871	19.01055836837038
C	11.83856958328560	7.25180455586576	20.51181616102468
C	11.76681700175594	6.00313313605850	21.22727025002749
H	12.60940024861149	5.49107145448901	21.66305585254935
C	10.46980787248139	5.62922847359105	21.24849139547164
H	10.04111559568273	4.75304580819818	21.70774853222248
C	9.72004300989284	6.65422736649471	20.56694586736473
C	8.33865027901828	6.66595343927533	20.44961562224188
C	7.56489414344840	7.73161952546053	19.91255611813400
C	6.15511854375369	7.75363670896206	19.83524220548729
H	5.49760860855248	6.96012777160874	20.15053112938244
C	5.80512740283224	8.96783071172522	19.28787415866872
H	4.81298053807996	9.33992667374281	19.09128413202562
C	7.00839323505424	9.65248790903227	19.01218819105949
C	7.09828277529946	10.92935837836966	18.39125681803439
C	8.27234378073043	11.56153883713207	18.01837987673085
C	8.33764865628517	12.78970415479556	17.26649496808764
H	7.48310979576777	13.37062730595254	16.95858228625193
C	9.64160138264318	13.03634320985368	17.02045887641017
H	10.06730136725237	13.86975046717291	16.48531898425497
C	10.39967709116460	11.96733781955794	17.62243120640696
C	11.78272916370848	11.87928641974374	17.61112456696450
C	12.55080243607214	10.88723824351721	18.28192755545276
C	13.96247836937161	10.82473794151682	18.29419305929716
H	14.62479196147785	11.50175735332084	17.78013880486342
C	14.30571629651840	9.74135807891749	19.07047156142049
H	15.29518923831943	9.37460889269330	19.29018735598965
C	13.09754610562659	9.15242007139445	19.50514303203724
C	13.00852162537654	7.94591004311665	20.24806956689114
C	7.60008473572932	5.48189572833962	20.94422213365801
C	7.73863905626461	4.25043982196035	20.30717172247540
H	8.38483273262912	4.17220430702473	19.43998540384529

C	7.05232846469687	3.13698539570923	20.76735496598558
H	7.16521170305379	2.18750932122574	20.25657077105681
C	6.22365742267558	3.23821341874199	21.87509866140142
H	5.68943480824155	2.36741545453547	22.23735625391311
C	6.08180934352037	4.46050215224086	22.51772809994321
H	5.44100930495942	4.54637545037095	23.38789073980710
C	6.76002987919572	5.57577698234907	22.05250087410279
H	6.64940953161417	6.52940080413617	22.55596919163717
C	5.82429680971368	11.62077652033968	18.08475461275919
C	5.47122369745425	12.77731430337927	18.77576999202273
H	6.13245106803337	13.15783721598322	19.54624123722837
C	4.28489389009958	13.43531345475109	18.48794939098415
H	4.02070718603069	14.33160469155085	19.03752565680639
C	3.44024759908707	12.94865222630032	17.50121138942669
H	2.51473645424950	13.46470626045286	17.27310088448267
C	3.78616017777074	11.79801246327950	16.80617701951259
H	3.13451349488683	11.41504348210778	16.02909648662033
C	4.96751644672603	11.13500999584467	17.09928546387089
H	5.23904644438648	10.23808763606824	16.55428319198852
C	12.52801389431427	12.90263615157435	16.84499537598961
C	13.36305096488256	13.81399791654060	17.48941362657787
H	13.46189399683252	13.77312205671538	18.56810862402811
C	14.05122684931836	14.77314662111912	16.76373223267219
H	14.68756865925990	15.48280405146590	17.28015883278833
C	13.92506438867411	14.82732711808488	15.38235305591392
H	14.46743046456607	15.57489144742785	14.81494130027204
C	13.10062643004304	13.92073269571985	14.73218091220612
H	12.99938618547639	13.95297869302131	13.65333481684879
C	12.40342257060489	12.96756065002287	15.45852934071377
H	11.76170892538848	12.25610076492706	14.95202143644060
C	14.28561362450932	7.36398485496828	20.71746616201028
C	15.05755346386800	8.03162018174702	21.66666257059773
H	14.70759479052023	8.97674796673628	22.06616721501554
C	16.25523662920780	7.49108269695700	22.10646129742605
H	16.83876277630441	8.01577870656041	22.85435850598327
C	16.70645967899443	6.28316792673264	21.59226813920314
H	17.64598959606802	5.86317853344611	21.93264273355111
C	15.95020339501063	5.61736689245413	20.63881354880611
H	16.29905461870329	4.67780248589843	20.22558199700545
C	14.74634487207174	6.15218446586988	20.20609050114998
H	14.15874858387036	5.63605517634753	19.45558002486604
C	9.98253519735822	8.45820402632151	17.40265037544890
H	10.12302909540088	7.38898091356740	17.59981630832702
H	8.96402356509759	8.63047284113797	17.04367063836101
C	13.01329393821011	6.98020354321648	16.79507556146137

H	13.74089254999117	7.64845437983698	17.26692032463274
H	12.36235442375443	6.63710398571659	17.60759783859983
C	13.71735101895753	5.78195095248756	16.16431478848105
H	13.00125569285859	5.07169421248880	15.74378862546764
H	14.32158255401295	5.24020179685920	16.89816995315885
H	14.38715872324189	6.08181072978646	15.35477090836340
C	11.00352542270424	6.87387097705568	14.48472648577301
H	11.71637546980489	6.29461168880032	13.88645455697706
H	10.47803277354910	6.14020493178295	15.10865603421438
C	10.01496083025882	7.60116036826106	13.57913623114565
H	10.52690713077957	8.29756402772297	12.90927436504455
H	9.29930844507061	8.18485186859085	14.16398766342170
H	9.44438475077724	6.90782627310136	12.95494550300028
C	12.97157199543330	9.26106494448118	14.66338717370819
H	12.26803516178597	9.85762975012272	14.07142181407669
H	13.41156181671613	9.95271678952833	15.39031960255338
C	14.05429788712780	8.67694120298477	13.76184201368170
H	14.56615387216560	9.45585506765287	13.19008546593955
H	13.64131024087570	7.96364585084146	13.04285215444505
H	14.81569229479518	8.14914790270285	14.34138531019460
C	10.01801823048002	10.35215983169461	21.01571839527173
O	9.97476946117781	10.91336295675770	21.99469109139644

(TPP)$RhCOCH_2OSi(CH_2CH_3)_3$, **10a**, S_0 state
E = -7450.950372709138 a.u.

Rh	5.78207378327315	8.52965676058793	14.94142082350071
Si	1.96040803742727	5.21189748831755	14.63391365537163
O	5.62642349673647	5.87426362030357	14.10287793440558
O	3.36359589257424	5.17851856576161	15.54591020401159
N	6.29080566391652	8.50634939869571	12.98281015236093
N	7.63667243999929	7.89591554110010	15.44104435883662
N	5.34956697833964	8.78731052943427	16.90627448070710
N	3.97596229809304	9.31897205623181	14.44694652554750
C	5.49988871184526	8.85593617916025	11.92732693913823
C	6.24318024338495	8.72088761723714	10.70455965158842
H	5.86917043001231	8.96588668508356	9.72350492783027
C	7.46863481575617	8.25558646408611	11.03681355996687
H	8.29832215631021	8.04343285960092	10.38181515186988
C	7.48557514426494	8.09961295896309	12.46545982990255
C	8.55930111008457	7.58589076094535	13.18556187649202
C	8.60312331048443	7.47824372720961	14.57175413716303
C	9.69729717053348	6.91028922131954	15.30888887270510
H	10.58562974065883	6.48676046919283	14.86864785670804
C	9.38405738406793	7.00629770136603	16.62149948629525
H	9.96493765634876	6.67633696741122	17.46772899184973

C	8.09434014506476	7.63326375083642	16.70157709453801
C	7.43342969381185	7.92980785583870	17.88949243839674
C	6.16805823960690	8.50431094136735	17.96430206920463
C	5.52437445211547	8.89545546902248	19.18636808774200
H	5.96197058409880	8.81418600447894	20.16843938833702
C	4.30933436063841	9.39102037736292	18.85459241393261
H	3.55582179872102	9.79500195393871	19.51154675327910
C	4.19554123121208	9.30694734172057	17.42620933955702
C	3.06507556306923	9.67716027816465	16.70392070272131
C	2.97175251211980	9.65088048103635	15.31476511872950
C	1.78572735882857	9.97385305633971	14.57335270244526
H	0.83881254588112	10.24882792289244	15.00939644344093
C	2.09330936029080	9.85610771494112	13.26038892059775
H	1.44658650739434	10.01396305546421	12.41241881400971
C	3.47141392802519	9.46174386985377	13.18353809336656
C	4.17170135181000	9.26470651498002	11.99752750448626
C	9.74080967837736	7.11531386919356	12.41950702913728
C	10.95263833182467	7.79750852364523	12.49324947457516
H	11.02517138842399	8.68384524235147	13.11363391706380
C	12.05461008243660	7.35659844967394	11.77647435590185
H	12.98973928667845	7.90138876066854	11.83913645503832
C	11.95995497558300	6.22451565992076	10.97995333668251
H	12.82178897202418	5.87825121755914	10.42115832788931
C	10.75680709138824	5.53760565806518	10.90233949196931
H	10.67614283862495	4.64936255768815	10.28617064055051
C	9.65381614515843	5.98133524081198	11.61567712432034
H	8.71433909957021	5.44292495814192	11.56020322924865
C	8.13144028247941	7.62906278565667	19.16467148385408
C	9.26972153621786	8.34199504487631	19.53312433323793
H	9.63948231044662	9.12464092684588	18.88019209713111
C	9.92160498161645	8.06338745066194	20.72460760227538
H	10.80305583126010	8.63085685141254	21.00057076559566
C	9.44607177941744	7.06456061173604	21.56202574932716
H	9.95676153434701	6.84485942816942	22.49249553422429
C	8.31355734192719	6.34818889540774	21.20255111666473
H	7.93809045467991	5.56311702433516	21.84891272594277
C	7.65939875287979	6.63059850164880	20.01303150150351
H	6.77679415773901	6.06784441336281	19.73013779647603
C	1.86730317298025	10.11010158721034	17.46622872265539
C	1.41692351906858	11.42614275467329	17.39833508219806
H	1.95871886623763	12.14101358778346	16.78916781500349
C	0.29357268820443	11.82441052995683	18.10669938675851
H	-0.04093559680782	12.85393484524373	18.04927024290282
C	-0.39796394074086	10.91000625682243	18.88826480232641
H	-1.27761933193155	11.22072986106038	19.44006866000930

C	0.04227124681581	9.59616934314991	18.96035063330376
H	-0.49488309688469	8.87454302867010	19.56520740289091
C	1.16894729484334	9.19995356934060	18.25635176994134
H	1.51082752024373	8.17243808775766	18.30920126201753
C	3.44955679260992	9.49091993994278	10.72127937001398
C	3.02654600901437	10.76579652650456	10.35354550134693
H	3.24040853999701	11.60439060101034	11.00670330346964
C	2.34669648361038	10.96671608031396	9.16198780740088
H	2.02949844861877	11.96595566809038	8.88614996151735
C	2.07639297247774	9.89425088198027	8.32411975715880
H	1.54269166526694	10.05091078842361	7.39380130865944
C	2.49439069178927	8.62057158416905	8.68221458737641
H	2.28497249905892	7.77647253271491	8.03505667099042
C	3.17892775733636	8.42122177623456	9.87105003255634
H	3.50480511002651	7.42659244946618	10.15329963145301
C	5.16167022289250	6.67152796920823	14.85207096784329
C	4.05946187739430	6.34220416274598	15.85211918145993
H	4.57405763916963	6.22789969122923	16.81439114074829
H	3.40493868332903	7.21026214201032	15.94886740574286
C	2.34002803601453	5.88169226699575	12.92299068244631
H	2.50888952040444	6.96133416165976	12.99852600422416
H	3.30187540058488	5.45408744276456	12.61995618845903
C	1.28110411505673	5.58954261033967	11.86265332215237
H	1.56916042037134	5.99359745035729	10.88858356982167
H	0.31350799672546	6.02606056330825	12.11858089768658
H	1.12741378920138	4.51461760872141	11.73725099451716
C	0.71578357784061	6.27265009901384	15.55835985681603
H	0.49879395254410	5.75621871096039	16.50134035385768
H	1.20758577358588	7.20999577258370	15.84112044119347
C	-0.58147167452677	6.59432890631186	14.82333360857239
H	-0.39375877200583	7.20902258187865	13.94004885713637
H	-1.27445425354506	7.14896030535294	15.46200058726044
H	-1.09780062949863	5.69030855746337	14.48912396813309
C	1.41514388374184	3.42752226645057	14.55771866115762
H	1.21867923763184	3.09637984825034	15.58380430097573
H	0.44925888042300	3.38894871159746	14.04040516389229
C	2.41856561019731	2.49680074820124	13.88242964831574
H	2.59900022631785	2.78782341912519	12.84424230037897
H	2.06883432770551	1.46093665618613	13.87568509033285
H	3.38287414670728	2.51897512468297	14.39512496527652

(TPP)RhCOCH$_2$OSi(CH$_2$CH$_3$)$_3$, **10a**, S$_1$ state

E = -7450.859060778788 a.u.

Rh	5.78287839794810	8.53924116800362	14.94250775613327
Si	1.95759021475916	5.26220407845150	14.63618071731560

O	5.59720933277752	5.88585891693308	14.14560834549192
O	3.32921114955326	5.23517079348350	15.59721257283814
N	6.27809854291094	8.48200816602681	12.96869197535652
N	7.65392264026094	7.91626831829174	15.43683981785753
N	5.36579919408819	8.82419527650615	16.91875189818211
N	3.97519869931858	9.35408572196782	14.45000529916640
C	5.47819360685710	8.82160300779103	11.91301637771182
C	6.21806954890452	8.70283345346975	10.69133734544052
H	5.84209283658711	8.95105656120237	9.71234947608146
C	7.45668074107322	8.25527318580046	11.02080952803903
H	8.28894972816571	8.06625184936784	10.36287943188051
C	7.47804032100427	8.09165013495728	12.44606590928876
C	8.56625189892006	7.58880418811365	13.17111766701638
C	8.61269752859715	7.48501064442170	14.56225564376652
C	9.69228933142940	6.88518991946455	15.29480026799142
H	10.56724674168449	6.43673962580151	14.85326943801390
C	9.37932749792995	6.97454374972592	16.61098612433251
H	9.94960590967575	6.61260112373323	17.45084994234450
C	8.10758510570706	7.63459502149243	16.69817498663304
C	7.45428490440242	7.94062968380700	17.89295185477409
C	6.19221537625076	8.54918019678169	17.97464170976236
C	5.57744075356372	8.98582607278697	19.19091373068034
H	6.02842461359783	8.92971671149107	20.16813564619834
C	4.36071045271470	9.49667166793935	18.86070708382979
H	3.62672980761496	9.93569452810821	19.51661235700504
C	4.22632529608718	9.37368217556330	17.44082706337513
C	3.08067335284306	9.73583341825103	16.71597298644315
C	2.97724965037167	9.69595305875835	15.32417603492944
C	1.77934023973618	9.98959628732541	14.58875748702430
H	0.83147918334413	10.25615180295143	15.02719411035874
C	2.07254765992055	9.84189988105510	13.27216284648141
H	1.40953591561293	9.96592003454678	12.43164218419705
C	3.45218438097874	9.46082907171285	13.18752361042219
C	4.13956740949101	9.23053215802470	11.99323009046892
C	9.74152436131243	7.12313622163907	12.40510781884730
C	10.98393728805059	7.73774840322986	12.56122324894744
H	11.07859141326804	8.57305554557070	13.24573498438564
C	12.08386691735740	7.29967737931362	11.83994667830736
H	13.04022468717374	7.79456751531427	11.96508083587300
C	11.96040721942553	6.23505094218274	10.95793641530387
H	12.82113836421101	5.89027883695617	10.39655474278991
C	10.72890361777979	5.61364665381379	10.79846485820368
H	10.62627834103435	4.77612064366175	10.11790384238479
C	9.62695667687874	6.05700797541598	11.51249391351809
H	8.66675081372259	5.56778905265759	11.39490463180236

C	8.13920683863115	7.61898627252873	19.16204238465196
C	9.36330208368356	8.20768533942598	19.48084122103085
H	9.80523194602992	8.91603867096124	18.78953766562956
C	10.00261504258798	7.90683031431438	20.67339115018716
H	10.94845674074470	8.38066437750062	20.91002002431405
C	9.43210838537275	7.00703682304117	21.56309470793526
H	9.93388729762288	6.76891779927193	22.49385135836559
C	8.21494211443970	6.41403477412888	21.25504412216891
H	7.76630233432700	5.70481272958004	21.94120258560288
C	7.57104723527931	6.72156834163605	20.06699076522855
H	6.62373122438742	6.25374717329906	19.82408922401048
C	1.88756118356564	10.14704966755472	17.48545001795839
C	1.34913832098093	11.42660561487406	17.35209099239357
H	1.82800226048687	12.13545453286861	16.68615155451928
C	0.22301382392152	11.79683565826577	18.07127115951954
H	-0.17764187178580	12.79837113675203	17.96384013438296
C	-0.38643653874961	10.89137638654684	18.92890695836267
H	-1.26887486395053	11.18002729794408	19.48816509420755
C	0.14034136959083	9.61408772146247	19.06734901449026
H	-0.33369136136007	8.89846015555741	19.72939699994066
C	1.27111296344110	9.24691137367086	18.35559912991226
H	1.67842240679125	8.24741784032777	18.45790213001501
C	3.40777131560420	9.41918430090584	10.72417010324463
C	2.88743872087639	10.66414026208967	10.37060746763579
H	3.03815318698119	11.50864722369075	11.03328486575940
C	2.19969937964331	10.82833881998953	9.17821123123288
H	1.81047864595830	11.80466320102504	8.91281562930517
C	2.01464108335736	9.74917109810260	8.32500694050044
H	1.47327302325954	9.87713622097247	7.39476775310287
C	2.52803594169561	8.50548165999813	8.66805395876478
H	2.38258854294029	7.65618704225052	8.01026901577899
C	3.22460863111132	8.34324500551401	9.85473373922534
H	3.62280014987087	7.37234341061289	10.12609376026500
C	5.14526203933326	6.69969070887000	14.88573895309454
C	4.03798382888135	6.39230863782326	15.89422966710859
H	4.55295604941595	6.27754212649836	16.85590639119004
H	3.39264317158735	7.26813009428589	15.98291370103641
C	2.41167110239504	5.83509457621955	12.90780023943176
H	2.60920108834432	6.91256967656811	12.93406016215011
H	3.36945347892113	5.36551632383744	12.65992290434692
C	1.37799004889836	5.51677095885985	11.82905822403043
H	1.71422613343172	5.85108052613005	10.84399530095793
H	0.41968661028459	6.00230688112597	12.02442920342074
H	1.18875934833195	4.44220092137021	11.76217696053402
C	0.71332393365555	6.39643669180204	15.46933416629943

H	0.43555999612855	5.91972031424275	16.41716392203476
H	1.22529424988951	7.32562795041540	15.74334024638689
C	-0.53630997654598	6.73785663802244	14.66269352591364
H	-0.28374428368450	7.32090225983048	13.77412239869138
H	-1.24019751342004	7.33270352175945	15.25128965582252
H	-1.06572291321947	5.84151569293888	14.32793345793981
C	1.37151738517748	3.48887492606053	14.62723125391355
H	1.14572244513092	3.20998565644997	15.66277408794067
H	0.41704736292507	3.44580840392804	14.08948765438683
C	2.37135487272448	2.50816061052586	14.01909218080765
H	2.58045216157855	2.74776699041400	12.97312208712662
H	2.00125622974816	1.47989687649111	14.05200975550105
H	3.32398217785194	2.53667725499933	14.55292101309143

(TPP)RhCOCH$_2$OSi(CH$_2$CH$_3$)$_3$, **10a**, T$_1$ state
E = -7450.885472172144 a.u.

Rh	5.78706062569520	8.53051301960477	14.93986394463670
Si	1.94311297675684	5.23032979904542	14.63443908844467
O	5.57784385481559	5.90803491021940	14.05670674806668
O	3.35412230765108	5.19186952908817	15.53611807815122
N	6.30155296955212	8.50518859501427	12.96146293771620
N	7.65468502562565	7.88229613868283	15.44111225438535
N	5.35003728865615	8.77896131424038	16.92422029216612
N	3.96726978383721	9.32912390095661	14.44231039603860
C	5.50267073934095	8.83734931528590	11.90547851267016
C	6.25314141558141	8.72264023295286	10.68021077373352
H	5.87715934192280	8.96624824474299	9.69968189202081
C	7.48765058677560	8.28974901244265	11.01154606643971
H	8.32357302604450	8.10756743356453	10.35572050724292
C	7.50770588095800	8.12291630040062	12.44260463588261
C	8.58049194170412	7.62134199487636	13.16012715828692
C	8.62589148214344	7.47108513237362	14.57520750379358
C	9.68804017087635	6.87504965291076	15.28597965596458
H	10.57574884742627	6.44871720516408	14.84816899257598
C	9.35717597494789	6.94023465614256	16.62196096142412
H	9.92955309170725	6.57685447376928	17.45971710642416
C	8.10183350907867	7.57887297454858	16.69851114999793
C	7.41947294935800	7.89100556967427	17.90411048736489
C	6.16663464268350	8.48533194661515	17.98151012432972
C	5.53591833435238	8.90280850729225	19.20462933057392
H	5.97755461396145	8.82843897505724	20.18521471394072
C	4.33076785364988	9.41884373820359	18.87468524708345
H	3.59194293378056	9.84811170215098	19.53208928801195
C	4.20585547819188	9.32079415758607	17.44548319683712

C	3.07972210581680	9.69725362073195	16.72679088683853
C	2.95932011367353	9.65761892233192	15.31023924290281
C	1.78268479401356	9.95026814843208	14.59256382499628
H	0.83334961757965	10.22086993991334	15.02516020532809
C	2.08925763676143	9.81457453331168	13.25403583793721
H	1.43109220336820	9.95399420518052	12.41231679892627
C	3.44684630213521	9.44411290491164	13.18441283570709
C	4.17349465105918	9.22649458601837	11.97698836539271
C	9.77422442715925	7.18263054196136	12.40485359364139
C	10.99561571448190	7.83493531549024	12.56754797264999
H	11.06327460997052	8.67342373923478	13.25128607964964
C	12.11044624986038	7.42813097679794	11.85256976184703
H	13.05018711244585	7.95357829952107	11.97869538994197
C	12.02514654103038	6.35301243507522	10.97843900062522
H	12.89941364981419	6.03154467044852	10.42415838805405
C	10.81666779941122	5.69078814379517	10.81935786230290
H	10.74349773117921	4.84520065080611	10.14505448319806
C	9.69702171048436	6.10523475670432	11.52423540118272
H	8.75329591611538	5.58515665867979	11.40547336794454
C	8.11137769960102	7.57579047852239	19.17231646567407
C	9.31176262639000	8.20542189475045	19.49949205225324
H	9.73410792109947	8.93134746530554	18.81437408163542
C	9.95271646366371	7.92037352951677	20.69406867066142
H	10.87819067272523	8.42785150890013	20.94116356406039
C	9.41268553328815	6.99009954668323	21.57168898693027
H	9.91818044502945	6.76306701464187	22.50324259930084
C	8.22387496646420	6.35159333417622	21.25051933318795
H	7.79904992375806	5.61863881023251	21.92676534407291
C	7.57442766263810	6.64632938329723	20.06156692356829
H	6.64695160578451	6.14531972442212	19.80850645083664
C	1.89081924059858	10.14347501801617	17.48451844748871
C	1.40672866912738	11.44407937028823	17.35100617136061
H	1.91871677528770	12.13562329782531	16.69177003407892
C	0.29142927890610	11.85543350257715	18.06257380149640
H	-0.06538821329261	12.87384336951161	17.95974123519575
C	-0.36668773525414	10.96837347675173	18.90359516504800
H	-1.24300304379610	11.28953380079360	19.45477246120855
C	0.10135955935862	9.66909150466151	19.03430722417889
H	-0.41120859723719	8.96815284434678	19.68317206025738
C	1.22548512623704	9.26039997028417	18.33349829430388
H	1.58923971176653	8.24388997451250	18.43157985431699
C	3.44239549786429	9.42100154863690	10.70764005415271
C	2.93845946028463	10.67156672461003	10.35224868722247
H	3.09390036887833	11.51488177528973	11.01525446504162
C	2.25962790127287	10.84206100809053	9.15662598825454

H	1.88581172486092	11.82321926971323	8.88697430185847
C	2.05978859901188	9.76288115519132	8.30642495928634
H	1.52307850978520	9.89654119742611	7.37424490701775
C	2.54996630004361	8.51307316885634	8.65569987562822
H	2.39320889774163	7.66355237769654	8.00085084350819
C	3.24116423083342	8.34421248787606	9.84528481184094
H	3.62232203981705	7.36781130928460	10.12173602067257
C	5.14184510192034	6.69258999835748	14.83568011112219
C	4.05302147511476	6.34998259601654	15.85073441061188
H	4.58026066946918	6.22085997810305	16.80398205185803
H	3.40061631753319	7.21747891030667	15.96523318609426
C	2.30914160253777	5.90937412019451	12.92400020462750
H	2.46666709519701	6.99035486070975	13.00695155760996
H	3.27333730265300	5.49251321419370	12.61380081324084
C	1.24762078398466	5.61627174317910	11.86648846105242
H	1.52786051139151	6.03227132015545	10.89496629205361
H	0.27747286020257	6.04211530994031	12.13082041157966
H	1.10258775552239	4.54120644317709	11.73171408238453
C	0.70693235206420	6.28817584935917	15.57363874375518
H	0.49615744555511	5.77030772777577	16.51727574026860
H	1.20132133194316	7.22576028161905	15.85190141272746
C	-0.59471639574380	6.61236204628065	14.84694855698748
H	-0.41076052933807	7.22918184696413	13.96435671863340
H	-1.28349266474544	7.16643216891244	15.49064200246814
H	-1.11392326955895	5.70961997691499	14.51347056621825
C	1.39935071304062	3.44505187173812	14.55572900053436
H	1.20819825516133	3.10938480969275	15.58137949836199
H	0.43097051717020	3.40761339140375	14.04286764668011
C	2.40039415067684	2.51785538129993	13.87185631222161
H	2.57608032474640	2.81323373008120	12.83406856931402
H	2.05137760689954	1.48173560081275	13.86236376319687
H	3.36697658062859	2.53861878816890	14.38035666752416

Non-covalent complex of [(TPP)Rh(II)]$^{\bullet}$ and $^{\bullet}CH_2OSi(CH_2CH_3)_3$, T_1 state

E = -7450.846716318104 a.u.

Rh	10.25252004989113	9.60935324680123	19.00932991998796
Si	11.18923250366293	7.07243363759755	15.96244124050108
O	9.89275998924614	7.99711561562602	16.53374162833332
N	10.84323761744204	7.92375482818364	19.96578834599781
N	8.31235812543272	9.09515204574981	19.29250708683460
N	9.66558724978586	11.32179322532003	18.09769643325988
N	12.19541158208541	10.15269963927916	18.77975550494780
C	12.12737680585195	7.55225285647383	20.24890662962946

C	12.11811824913325	6.32229932444069	20.99254659874971
H	12.99490358788232	5.82047413091822	21.36949255728391
C	10.82419032308992	5.94922478674452	21.12457428963515
H	10.43253489966851	5.08232766241682	21.63238574955479
C	10.02841777772514	6.95022756060590	20.46883341287635
C	8.64008614581877	6.91243958912070	20.37349549919718
C	7.85680662032990	7.91823830201333	19.81457659049397
C	6.42652123473088	7.85961390281528	19.69018404013650
H	5.81154884054728	7.03064068983178	20.00185241561761
C	6.03150258122961	9.01495782200733	19.10706353864713
H	5.02983058499563	9.31734818087873	18.84660146160018
C	7.21735167284424	9.78939348985532	18.86213725455701
C	7.22780929763345	11.03568888679533	18.24103307239221
C	8.37995287479014	11.74567299510240	17.91264466900292
C	8.38712476073175	13.04976510925210	17.30824740990597
H	7.50778889838541	13.62874208895845	17.07555722909297
C	9.68245882348876	13.38972041178090	17.11468748730153
H	10.07291855033434	14.30202642054612	16.69284742974004
C	10.48125397412173	12.29945990975025	17.60250154555204
C	11.87299613699547	12.26891516753138	17.56961649859543
C	12.65561862977664	11.25318087003614	18.11145107692431
C	14.08945136754537	11.20521860671653	18.03915168280393
H	14.70914038978355	11.93740089663331	17.54679682619108
C	14.48209137864765	10.08085068730242	18.68227475481156
H	15.48596803664716	9.71153265328259	18.81780608984403
C	13.29298150776638	9.42777708852541	19.15336471512638
C	13.28059255144544	8.22418523220424	19.85340707252943
C	7.95018901010410	5.70304447345099	20.88999660472448
C	8.10161278077136	4.48067190704106	20.23883532312466
H	8.72326098270889	4.42789381850870	19.35170537243077
C	7.45933154792119	3.34573225311195	20.70972545214007
H	7.58243016317544	2.40300541210712	20.18867941313862
C	6.65886665764774	3.41682572686956	21.84098681850192
H	6.15750839267632	2.52958202690005	22.21037935541586
C	6.50346156060658	4.62963821426210	22.49693677359501
H	5.88335452610486	4.69281133955420	23.38394466954664
C	7.14356364243631	5.76506986136482	22.02341414958922
H	7.02477135950865	6.71178772236412	22.53860148725838
C	5.91882768941673	11.64023159480067	17.88482497370298
C	5.03937991366736	12.06597771826362	18.87721501258104
H	5.32243786843166	11.95857389456908	19.91844770860031
C	3.81741691434031	12.62977528027728	18.54243839790414
H	3.14617620598705	12.96235167782123	19.32611483535549
C	3.45685571965406	12.77121867600101	17.21003839886100
H	2.50111145623344	13.21059627779949	16.94821873610922

C	4.32563045692360	12.34696023197593	16.21470932164528
H	4.04920507514572	12.44904264867806	15.17134194930061
C	5.54905993152295	11.78668561000942	16.54937518569872
H	6.22723907614480	11.44844291307651	15.77409509245076
C	12.57173183207298	13.40243061590551	16.91133175223719
C	13.32928128265896	14.30092133766155	17.65868712855929
H	13.40087170327000	14.16846050629850	18.73250899783964
C	13.97959300562846	15.35934901030461	17.04252361532269
H	14.56056923311611	16.05371908838968	17.63886360347729
C	13.88378206579454	15.53159913370847	15.66902069659657
H	14.39290481866343	16.35817927397976	15.18674153466139
C	13.13276977078037	14.64030849607367	14.91611932642358
H	13.05633756937217	14.76519545299397	13.84193224996342
C	12.47984672121698	13.58400166320794	15.53327147857541
H	11.89667010335184	12.88458180724106	14.94504196148535
C	14.58830486164257	7.59519839893512	20.17086163656649
C	15.45640865143579	8.17608147591653	21.09179600944297
H	15.16461171792503	9.09540250419650	21.58718501542638
C	16.67764138671181	7.58579177813304	21.38101671947575
H	17.33945789669876	8.04742822599991	22.10501432753328
C	17.04970110308136	6.40786937445981	20.74928508985625
H	18.00479928706970	5.94723907949225	20.97408270475149
C	16.19210773703258	5.82223838026009	19.82899280816236
H	16.47626583949868	4.90368967017310	19.32809214357898
C	14.96953639160898	6.41075586463947	19.54371285344188
H	14.30135108451052	5.95325967388788	18.82272394168443
C	8.61309864810323	7.58384688868961	16.41775846000639
H	8.37227009995318	6.81201945235633	15.69562173325425
H	7.86016584977504	8.24664175460760	16.81754302008414
C	11.47775949124368	5.70484606026638	17.20667984133040
H	11.87837913806920	6.15864214519743	18.11763305937947
H	10.48471419036510	5.32901230418665	17.48309609211298
C	12.36327570544304	4.55001069429453	16.74957427459796
H	11.94948291296128	4.04977551298719	15.87041700613339
H	12.46966935567531	3.79607432874785	17.53442002172790
H	13.36828108031308	4.88779305748697	16.48505647561802
C	10.74808301477021	6.33681993390964	14.29519844333851
H	11.64944967575742	5.81755454603662	13.94542347455326
H	9.99965546489123	5.54835262859590	14.43524339935812
C	10.28065590312587	7.33740694140447	13.24320692862996
H	11.02334282730615	8.12177708896703	13.07707281874372
H	9.35369862995646	7.82987922040076	13.54685337550461
H	10.09500732988146	6.85317115159603	12.28085580176537
C	12.57907420802732	8.30525832801088	15.81308163607502
H	12.33470639990127	8.97283067948859	14.97957053690351

H	12.55658439873301	8.93104059526671	16.70807459051654
C	13.97091525833866	7.71016244472477	15.62837813147426
H	14.72048799653437	8.49416415688003	15.49476505563792
H	14.02551421054880	7.05266386337254	14.75589875737711
H	14.27200081884418	7.12460091915398	16.50011414365336
C	8.89304611534695	10.63287857826468	14.83614120638293
O	9.99007608237353	10.77208556334118	14.63619974144273

Non-covalent complex of [(TPP)Rh(II)]$^{\bullet}$ and the $^{\bullet}$CO…$CH_2OSi(CH_2CH_3)_3$ transition state, T_1 state

E = -7450.845403690033 a.u.

Rh	10.22023302405968	9.54265719465574	19.03569675580640
Si	11.17685594627864	7.19713255491268	15.95910441702312
O	9.97999407217683	8.24176270836248	16.56662500089092
N	10.83067853220116	7.87399871127196	20.00904846780900
N	8.28542180119767	9.00999169124288	19.33313929911301
N	9.61070169422544	11.25021186614462	18.13265382899410
N	12.15514013914227	10.09633804525954	18.78046810756759
C	12.12001283742008	7.51863562666710	20.28903628034161
C	12.12700877242707	6.30419007511049	21.05710933744849
H	13.01048220544303	5.81833044667776	21.43909587954595
C	10.83749812123859	5.92208766016738	21.20517670401277
H	10.45766893808833	5.06282916051107	21.73432796628977
C	10.02836421702079	6.90178442884885	20.53381887229007
C	8.64020109603223	6.84425429600781	20.43968248135781
C	7.84407848879119	7.83186448822435	19.86568768365870
C	6.41343212116087	7.75990458125756	19.75070900361287
H	5.80733297071048	6.92753272963294	20.07050044807774
C	6.00361448355568	8.91047019793544	19.16824399857224
H	4.99721562466212	9.20305961274624	18.91526141004731
C	7.18011278284532	9.69558503313954	18.91372384005895
C	7.17687581367752	10.94156539299088	18.29107765348391
C	8.32059809973087	11.66257521376264	17.95739147975856
C	8.31117803675428	12.96763968536540	17.35426003494677
H	7.42505848068583	13.53886814180515	17.12875294080584
C	9.60121251420846	13.31876799901442	17.15111089213706
H	9.98023590426388	14.23424504115933	16.72614745907955
C	10.41320924929287	12.23305221663466	17.62811563322532
C	11.80363419989875	12.20640609248167	17.56826126195289
C	12.59927594985361	11.19307659751995	18.09655306865297
C	14.03151148826922	11.14630395650967	17.99433344187521
H	14.63963416769548	11.87427305300764	17.48194600584661
C	14.43932159335036	10.02761240514021	18.63783464722241
H	15.44641059018202	9.66079580856313	18.75423458335258
C	13.26094812782925	9.37761755139856	19.14011049031767

C	13.26416327419252	8.18641276797571	19.86138357787177
C	7.96555377045203	5.64093770147405	20.98846167334591
C	8.14827838393978	4.39725060106590	20.38766996331191
H	8.78423415082863	4.32027246012772	19.51259671829544
C	7.52085822033161	3.26922475937491	20.89366860431977
H	7.66860125855125	2.30975188145544	20.41111712710104
C	6.70429418838212	3.36835338945485	22.01109552473464
H	6.21466198983577	2.48656037132823	22.40811806832521
C	6.51826288239412	4.60211528204163	22.61799104417110
H	5.88618245657334	4.68741677706270	23.49458859954160
C	7.14310180906434	5.73038605351972	22.10883732727667
H	7.00096282230753	6.69348042511886	22.58617617413950
C	5.86262432358637	11.52415177106570	17.92148043181097
C	4.96275916727716	11.93636580680189	18.90087992539900
H	5.23465361551374	11.83814291172167	19.94594204857246
C	3.73489573879489	12.47544879097271	18.54794992536779
H	3.04721734812244	12.79837701740971	19.32131402609318
C	3.38924173411668	12.60491088105166	17.21045225945038
H	2.42877916003211	13.02484964836793	16.93449188147479
C	4.27922363743319	12.19485544255463	16.22791856504538
H	4.01507749464920	12.28914607091014	15.18071358978918
C	5.50870517128933	11.65981912532907	16.58060166770947
H	6.20537307012570	11.33624547928965	15.81475969474548
C	12.48529476313750	13.33653976884810	16.88841492045180
C	13.27894469063912	14.22699321936147	17.60728794474872
H	13.39077385352140	14.09201622204836	18.67732137645609
C	13.91309478880682	15.28145793560993	16.96812708695576
H	14.52222789174912	15.97018811926791	17.54249140894316
C	13.76494023439875	15.45712459453690	15.59975767217060
H	14.26148054172929	16.28059940549983	15.09940347800913
C	12.97735756009378	14.57378598417013	14.87533471976396
H	12.85954412499334	14.70179241794498	13.80527269502983
C	12.33996400737158	13.52178243673155	15.51523775400881
H	11.72701281881115	12.82864713056753	14.95050832547854
C	14.57849880795315	7.56739269168587	20.16665900523377
C	15.47197449030907	8.18051163181148	21.04127494375025
H	15.19379731225309	9.11732121934802	21.51091664565108
C	16.70089109561341	7.60036554791197	21.31711369109541
H	17.38271802051053	8.08754128338281	22.00484740991334
C	17.05533571151467	6.40012327711555	20.71818826721968
H	18.01678140177259	5.94762522589417	20.93202383680796
C	16.17215147845342	5.78174295989500	19.84472885442078
H	16.44261253150606	4.84549385783915	19.36972753287268
C	14.94181652516231	6.36013831806455	19.57309749918632
H	14.25324187741723	5.87721413479432	18.88853410616607

C	8.76558635718860	8.44599343558312	16.04183747583911
H	8.44409761778948	7.81382751095901	15.21998785221143
H	8.02622921276040	8.84739090762596	16.72079867536447
C	11.35008727568497	5.85694745864839	17.25137691031178
H	11.82318011194255	6.30617899995741	18.12841996917561
H	10.33505291055543	5.59484358415173	17.57243783632305
C	12.10891572449315	4.60190268516078	16.83122517993838
H	11.61244614302832	4.08514864643989	16.00639607189949
H	12.18599801210161	3.89350458018356	17.66049227699306
H	13.12594124482181	4.83070868910607	16.50388529039412
C	10.62873273186987	6.44209377590911	14.33460022585088
H	11.36168087121331	5.64958744182365	14.13604506119167
H	9.68041834436123	5.91465438849178	14.49210809887889
C	10.54134007589569	7.36754507351439	13.12594100879779
H	11.48889927507029	7.88140408154044	12.94830704018821
H	9.77905712641848	8.13829418727688	13.25498335457835
H	10.29372508505356	6.81337685620857	12.21693496717467
C	12.68707069778003	8.27064887363611	15.75328489112401
H	12.50932193878244	8.92351122938645	14.89178366078813
H	12.73229632343615	8.93447640555170	16.61961346300618
C	14.00973661182928	7.52784419445977	15.59830514021536
H	14.83369501507003	8.22277439264901	15.41781654682359
H	13.99072876887245	6.81962174679698	14.76497035496264
H	14.25588794336139	6.96582619110334	16.50228256911937
C	8.92186523036543	10.43148290178449	14.81145155141324
O	10.02234553036367	10.70940249011001	14.72318594001885

Non-covalent complex of [(TPP)Rh(II)]$^•$ and $^•COCH_2OSi(CH_2CH_3)_3$, T_1 state

E = -7450.870115548707 a.u.

Rh	10.21287456140092	9.52753938342769	19.02709815760531
Si	11.17773362663400	7.20876873195681	16.01487690754223
O	10.04172049435386	8.29509030200072	16.63348436408741
N	10.82891793577953	7.86973126810793	20.01425443000786
N	8.27947764974074	8.98083162795972	19.31201468797872
N	9.59372961600263	11.23840954716696	18.13267476673953
N	12.14374590704059	10.09540505727566	18.78109760114697
C	12.11818500836298	7.53139140696988	20.31375899136121
C	12.12925336351489	6.32006375619509	21.08698041973591
H	13.01367072570989	5.84604876880075	21.48195279769306
C	10.84226940074527	5.92327146436964	21.21913292815767
H	10.46529799059359	5.06135564329320	21.74613600060266
C	10.03069147080051	6.89107565469673	20.53268438647123
C	8.64433743523278	6.81695600039685	20.41889819306953

C	7.84391609522086	7.79683300057991	19.83675171084304
C	6.41404898904778	7.71639451641430	19.71479180612564
H	5.81172965599412	6.87826692130668	20.02682334692548
C	5.99859300345900	8.86982402557094	19.14131340010268
H	4.99069982911497	9.15890837304248	18.88982811376354
C	7.17094919203875	9.66513838806316	18.89774692817301
C	7.16076586695248	10.92391184341411	18.29884083371631
C	8.30126562283729	11.65364925222894	17.97111799115478
C	8.28946222486149	12.96402717045139	17.37956632396180
H	7.40297263605890	13.53989035239984	17.16738115870413
C	9.57853873962753	13.31293423662774	17.16475234871375
H	9.95531913627908	14.23019335737060	16.74144111510715
C	10.39312411578338	12.22323786290667	17.62722743186658
C	11.78298382599947	12.19425743477836	17.55377104356728
C	12.58333504310200	11.18700970724021	18.08696671853241
C	14.01734691172439	11.15229511622222	17.99774778447522
H	14.62332937123091	11.87904916010169	17.48107387587431
C	14.42991118052273	10.04902876397461	18.66447805405017
H	15.43940585928561	9.69516466656649	18.80000709982467
C	13.25213296683424	9.39354519158837	19.16249373874752
C	13.25953394069890	8.21040951230409	19.89679186713762
C	7.97662225724798	5.60557017977767	20.95956846194938
C	8.18005851033758	4.36456747551591	20.35951516429411
H	8.82690171291193	4.29577242796381	19.49167296214632
C	7.56000413061321	3.22873436549731	20.85735172419491
H	7.72448373127786	2.27157408322668	20.37555199984049
C	6.72982795441305	3.31700722386544	21.96569116510413
H	6.24599715762787	2.42914189206822	22.35632022328926
C	6.52333432865326	4.54785576414258	22.57205302570748
H	5.88089068363585	4.62484762013361	23.44190812095014
C	7.14118317242653	5.68380435808630	22.07130148990983
H	6.98327610011892	6.64435683111843	22.54891585851865
C	5.84199627170770	11.51193677750448	17.95286145908122
C	4.94999703328858	11.89752207096924	18.95047728477470
H	5.23154174240787	11.77303703942321	19.99016938306639
C	3.71802151177713	12.44335910742307	18.62299145759483
H	3.03723121970748	12.74483030664038	19.41097172229634
C	3.35957112294707	12.60720582663531	17.29260511722172
H	2.39606244612642	13.03275893153157	17.03652812878455
C	4.24091772147522	12.22396763642895	16.29165669737752
H	3.96723994390275	12.34515372855615	15.24965937719641
C	5.47417450897669	11.68194662057689	16.61967787371986
H	6.16340741162671	11.38092455418426	15.83798357296099
C	12.45613674010680	13.31729759556142	16.85346319305315
C	13.25280495342030	14.22209769736802	17.55074769556701

H	13.37549838475789	14.10501846288127	18.62175481918626
C	13.87656673009702	15.26878044185084	16.88867845849488
H	14.48837912751131	15.96904475556029	17.44609379479343
C	13.71446148900231	15.42198915998654	15.51909681185603
H	14.20302065987291	16.23928352246754	15.00093613565506
C	12.92301779123724	14.52435265713674	14.81652233629825
H	12.79414453513673	14.63500265855598	13.74575639281944
C	12.29583410967682	13.48004709913825	15.47891814768184
H	11.67940913325345	12.77491404377122	14.93289457600355
C	14.57809982767762	7.61016785750204	20.22387385846788
C	15.43670838032364	8.22129326383760	21.13380925983516
H	15.12937328203342	9.14323040744533	21.61473520169595
C	16.66866078506557	7.65724758731320	21.42996200591281
H	17.32352491911575	8.14186511655443	22.14520128295922
C	17.06068517182712	6.47652561039043	20.81571831389358
H	18.02431460033852	6.03668999612411	21.04575956574154
C	16.21277216436167	5.86155514993665	19.90553701267974
H	16.51321284624014	4.94115645680941	19.41770528370548
C	14.97944877618457	6.42374552786372	19.61342017474064
H	14.31817765571544	5.94557444089581	18.89911006620589
C	8.92906862291958	8.78107554106571	15.95137009460118
H	8.49872372757955	8.04792663009505	15.25655945794749
H	8.13963970868596	9.03296236643309	16.66381068544173
C	11.32302516189639	5.86382683755885	17.30866846146056
H	11.88313607409920	6.27568905441263	18.15232741354878
H	10.31273775417596	5.68305111446886	17.69289004245215
C	11.95172916747195	4.54852767986776	16.85709829811426
H	11.36019535175686	4.06481115892998	16.07620146563467
H	12.02945174092579	3.84432426904691	17.69005000355058
H	12.95903964134335	4.69079908898679	16.45814401327898
C	10.56343269146326	6.46202778044816	14.40460809840639
H	11.20743651205648	5.58906566027162	14.24020124923646
H	9.56229889811468	6.04369689920847	14.56620140360489
C	10.58900007640077	7.34311766853331	13.15947183707620
H	11.58622981023649	7.75272754395226	12.98222861154821
H	9.90688482700382	8.19195395332677	13.23712293251932
H	10.30241175991278	6.78037138334817	12.26719161771600
C	12.75631142611161	8.17014790926436	15.75339033539899
H	12.55916747268017	8.91738719710304	14.97693905136397
H	12.94723882752246	8.74187475173160	16.66552253625976
C	13.98166962053593	7.33581227207790	15.39526803259659
H	14.85306975712990	7.96899374296020	15.20935206776882
H	13.81917295306442	6.73228717649505	14.49752209114338
H	14.24802273371892	6.65122683766115	16.20436169366969
C	9.17224761660762	10.01727696397019	15.12505031157720

O	10.17969048783491	10.56423183279041	14.86368773328933
